# Supplementary material for: Cyclin-Dependent Kinase 18 Controls Trafficking of Aquaporin-2 and Its Abundance through Ubiquitin Ligase STUB1, Which Functions as an AKAP
Source: Cells. 2020 Mar 10;9(3):673. doi: 10.3390/cells9030673 (PMC7140648; doi:10.3390/cells9030673)
Supplement: Supplementary file 1 [file cells-09-00673-s001.pdf]

Supplementary Materials

Cyclin-dependent kinase 18 controls trafficking of aquaporin-2 and its abundance through ubiquitin ligase STUB1, which functions as an AKAP

Dema Alessandro[1,2¶], Dörte Faust[1¶], Katina Lazarow[3], Marc Wippich[3], Martin Neuenschwander[3], Kerstin Zühlke[1], Andrea Geelhaar[1], Tamara Pallien[1], Eileen Hallscheidt[1], Jenny Eichhorst[3], Burkhard Wiesner[3], Hana Černecká[1], Oliver Popp[1], Philipp Mertins[1], Gunnar Dittmar[1], Jens Peter von Kries[3], Enno Klussmann[1,4*]

¶These authors contributed equally to this work

[1]Max Delbrück Center for Molecular Medicine in the Helmholtz Association (MDC), Robert-Rössle-Strasse 10, 13125 Berlin, Germany

[2]current address: University of California, San Francisco, 513 Parnassus Avenue, CA 94122 USA

[3]Leibniz-Forschungsinstitut für Molekulare Pharmakologie (FMP), Robert-Rössle-Strasse 10, 13125 Berlin, Germany

[4]DZHK (German Centre for Cardiovascular Research), Partner Site Berlin, Oudenarder Strasse 16, 13347 Berlin, Germany

***Corresponding author**

Enno Klussmann

Max Delbrück Center for Molecular Medicine Berlin

in the Helmholtz Association (MDC)

Robert-Rössle-Str. 10, 13125 Berlin

Germany

Tel. +49-30-9406 2596

FAX +49-30-9406 2593

E-mail: enno.klussmann@mdc-berlin.de

Content

1. CELL-BASED SCREENING BY AUTOMATED IMMUNOFLUORESCENCE MICROSCOPY 3

1.1 Screening plates 3

1.2 Image analysis using CellProfiler 17

1.4 Identification of siRNA affecting cell viability 18

1.7 Hits 18

2. SUPPLEMENTARY TABLE S4, FIGURES S2-S4 20

1. Cell-based screening by automated immunofluorescence microscopy

1.1 Screening plates

Table S1. Genes targeted with the Mouse Protein Kinases siRNA sub-library. Genes are sorted by plate and well. Accessions refer to National Center for Biotechnology Information (NCBI, BLA) entries. The siRNAs were arranged on three 384-well microtitre platres. The screening was carried out in triplicates). The table shows viability upon silencing of each candidate. NT#2-transfected cells were considered as 100 % viable. MCD4 cell viability of less than 60 % and more than 100 % is highlighted.

Plate	Well	Viability (%)	Gene name	Accession	Cat no.	Pool no.
1	A03	85	Cytidine monophosphate (UMP-CMP) kinase 1	NM_025647	D-063389-01	M-063389-00
1	A04	60	Integrin-linked kinase-associated serine/threonine phosphatase 2C	NM_023343	D-048470-01	M-048470-00
1	A05	78	PDZ and LIM domain 5	NM_019808	D-047511-01	M-047511-00
1	A06	71	Adenylate kinase 8	XM_130050	D-045649-01	M-045649-00
1	A07	82	NUAK family SNF1-like kinase 2	NM_028778	D-051199-01	M-051199-00
1	A08	86	SCY1-like 3 (S. Cerevisiae)	NM_028776	D-058290-01	M-058290-00
1	A09	87	Phosphoribosyl pyrophosphate synthetase 1-like 1	NM_029294	D-056856-01	M-056856-00
1	A10	103	Serine/threonine kinase 35	NM_183262	D-042007-01	M-042007-00
1	A11	43	Serine/threonine kinase 36 (fused homolog Drosophila)	NM_175031	D-056017-01	M-056017-00
1	A12	68	Phosphoenolpyruvate carboxykinase 2 (mitochondrial)	NM_028994	D-062380-01	M-062380-00
1	A13	69	Cyclin-dependent kinase 5 regulatory subunit 2 (p39)	NM_009872	D-049225-01	M-049225-00
1	A14	80	CDK5 regulatory subunit associated protein 1	NM_025876	D-056922-01	M-056922-00
1	A15	99	CDK5 regulatory subunit associated protein 3	NM_030248	D-046463-01	M-046463-00
1	A16	88	Cyclin-dependent kinase 6	NM_009873	D-043101-01	M-043101-00
1	A17	93	Cyclin-dependent kinase 7	NM_009874	D-040601-01	M-040601-00
1	A18	90	Cyclin-dependent kinase 8	NM_153599	D-053848-01	M-053848-00
1	A19	86	Cyclin-dependent kinase 9 (CDC2-related kinase)	NM_130860	D-040602-01	M-040602-00
1	A20	95	Cyclin-dependent kinase-like 1 (CDC2-related kinase)	NM_183294	D-042035-01	M-042035-00
1	A21	82	Cyclin-dependent kinase-like 2 (CDC2-related kinase)	NM_016912	D-040603-01	M-040603-00
1	A22	55	Cyclin-dependent kinase-like 3	NM_153785	D-054266-01	M-054266-00
1	B03	80	Dual-specificity tyrosine-(Y)-phosphorylation regulated kinase 2	NM_001014390	D-065611-01	M-065611-00
1	B04	90	Inositol 13456-pentakisphosphate 2-kinase	NM_199056	D-050560-01	M-050560-00
1	B05	88	WNK lysine deficient protein kinase 2	XM_127323	D-045498-01	M-045498-00
1	B06	91	Serine/threonine kinase 40	NM_028800	D-060998-01	M-060998-00
1	B07	87	Acyl-Coenzyme A dehydrogenase family member 10	NM_028037	D-047158-01	M-047158-00
1	B08	78	RIKEN cdna 2610018G03 gene	NM_133729	D-051436-01	M-051436-00
1	B09	91	STE20-related kinase adaptor alpha	NM_028126	D-044725-01	M-044725-00
1	B10	102	Acylglycerol kinase	NM_023538	D-046451-01	M-046451-00
1	B11	89	Cyclin-dependent kinase 19	NM_198164	D-059630-01	M-059630-00
1	B12	90	CDC42 small effector 2	NM_178626	D-057779-01	M-057779-00
1	B13	92	Cyclin-dependent kinase inhibitor 1A (P21)	NM_007669	D-058636-01	M-058636-00
1	B14	83	Cyclin-dependent kinase inhibitor 1B	NM_009875	D-040178-01	M-040178-00
1	B15	99	Cyclin-dependent kinase inhibitor 1C (P57)	NM_009876	D-062494-01	M-062494-00
1	B16	89	Cyclin-dependent kinase inhibitor 2B (p15 inhibits CDK4)	NM_007670	D-060290-01	M-060290-00
1	B17	95	Cyclin-dependent kinase inhibitor 2C (p18 inhibits CDK4)	NM_007671	D-061423-01	M-061423-00
1	B18	71	Cyclin-dependent kinase inhibitor 2D (p19 inhibits CDK4)	NM_009878	D-062496-01	M-062496-00
1	B19	93	Cyclin-dependent kinase inhibitor 3	XM_354809	D-047568-01	M-047568-00
1	B20	84	Ceramide kinase	NM_145475	D-040107-01	M-040107-00
1	B21	85	Checkpoint kinase 1	NM_007691	D-058640-05	M-058640-01
1	B22	83	Checkpoint kinase 2	NM_016681	D-040604-01	M-040604-00
1	C03	70	Cyclin-dependent kinase 2 interacting protein	NM_026048	D-045958-01	M-045958-00

1	C04	67	Adaptor protein phosphotyrosine interaction PH domain and leucine zipper containing 1	NM_145221	D-053641-01	M-053641-00
1	C05	58	Membrane associated guanylate kinase WW and PDZ domain containing 3	NM_133853	D-051390-01	M-051390-00
1	C06	70	IQ motif containing H	XM_134960	D-061985-01	M-061985-00
1	C07	89	RIKEN cdna 4930444A02 gene	NM_029037	D-059713-01	M-059713-00
1	C08	74	Tumor protein D52-like 3	NM_025741	D-049852-01	M-049852-00
1	C09	91	Testis-specific serine kinase 4	NM_027673	D-057012-01	M-057012-00
1	C10	90	Phosphoribosyl pyrophosphate synthetase-associated protein 1	XM_181343	D-064396-01	M-064396-00
1	C11	73	Doublecortin-like kinase 2	NM_027539	D-041657-01	M-041657-00
1	C12	68	Mixed lineage kinase domain-like	XM_356104	D-061420-01	M-061420-00
1	C13	64	Choline kinase alpha	NM_013490	D-065301-01	M-065301-00
1	C14	81	Choline kinase beta	NM_007692	D-065344-01	M-065344-00
1	C15	71	Cholinergic receptor muscarinic 1 CNS	NM_007698	D-058643-01	M-058643-00
1	C16	103	Conserved helix-loop-helix ubiquitous kinase	NM_007700	D-041014-01	M-041014-00
1	C17	92	Citron	NM_007708	D-044420-01	M-044420-00
1	C18	85	Creatine kinase brain	NM_021273	D-062038-01	M-062038-00
1	C19	82	Creatine kinase muscle	NM_007710	D-044437-01	M-044437-00
1	C20	84	Creatine kinase mitochondrial 1 ubiquitous	NM_009897	D-049314-01	M-049314-00
1	C21	84	Creatine kinase mitochondrial 2	NM_198415	D-042830-01	M-042830-00
1	C22	83	CDC28 protein kinase 1b	NM_016904	D-043790-01	M-043790-00
1	D03	77	Ankyrin repeat and kinase domain containing 1	NM_172922	D-052432-01	M-052432-00
1	D04	76	PITPNM family member 3	XM_354620	D-063825-01	M-063825-00
1	D05	75	Eph receptor A10	NM_177671	D-053504-01	M-053504-00
1	D06	85	Lemur tyrosine kinase 2	XM_132499	D-053835-01	M-053835-00
1	D07	82	TBC1 domain containing kinase	NM_173032	D-055508-01	M-055508-00
1	D08	90	Uridine-cytidine kinase 2	NM_030724	D-047637-01	M-047637-00
1	D09	70	DBF4 homolog (S. Cerevisiae)	NM_013726	D-046785-01	M-046785-00
1	D10	96	Abl-interactor 1	NM_007380	D-056816-01	M-056816-00
1	D11	70	C-abl oncogene 1 non-receptor tyrosine kinase	NM_009594	D-040285-01	M-040285-00
1	D12	75	V-abl Abelson murine leukemia viral oncogene homolog 2 (arg Abelson-related gene)	XM_136360	D-044204-01	M-044204-00
1	D13	76	CDC28 protein kinase regulatory subunit 2	NM_025415	D-045245-01	M-045245-00
1	D14	75	CDC-like kinase 2	NM_007712	D-040830-01	M-040830-00
1	D15	79	CDC-like kinase 3	NM_007713	D-040838-01	M-040838-00
1	D16	91	CDC like kinase 4	NM_007714	D-040839-01	M-040839-00
1	D17	77	Connector enhancer of kinase suppressor of Ras 1	XM_110525	D-050791-01	M-050791-00
1	D18	80	Coenzyme A synthase	NM_027896	D-062384-01	M-062384-00
1	D19	67	Collagen type IV alpha 3 (Goodpasture antigen) binding protein	NM_023420	D-054738-01	M-054738-00
1	D20	25	Coatomer protein complex subunit beta 2 (beta prime)	NM_015827	D-045228-01	M-045228-00
1	D21	84	Cyclin-dependent kinase 12	NM_026952	D-064510-01	M-064510-00
1	D22	80	V-crk sarcoma virus CT10 oncogene homolog (avian)-like	NM_007764	D-058646-01	M-058646-00
1	E03	80	Activin A receptor type 1	NM_007394	D-042047-01	M-042047-00
1	E04	83	Activin A receptor type 1B	NM_007395	D-043507-01	M-043507-00
1	E05	69	Activin receptor IIA	NM_007396	D-040676-01	M-040676-00
1	E06	64	Activin receptor IIB	NM_007397	D-040629-01	M-040629-00
1	E07	72	Activin A receptor type II-like 1	NM_009612	D-043004-01	M-043004-00
1	E08	81	A disintegrin and metallopeptidase domain 9 (meltrin gamma)	NM_007404	D-041144-01	M-041144-00
1	E09	78	Aarf domain containing kinase 2	NM_178873	D-057241-01	M-057241-00
1	E10	79	Aarf domain containing kinase 4	NM_133770	D-057610-01	M-057610-00
1	E11	84	Aarf domain containing kinase 5	NM_172960	D-055456-01	M-055456-00
1	E12	82	Adenosine kinase	NM_134079	D-062728-01	M-062728-00
1	E13	74	Colony stimulating factor 1 receptor	NM_007779	D-044650-01	M-044650-00
1	E14	80	C-src tyrosine kinase	NM_007783	D-060300-01	M-060300-00
1	E15	60	Casein kinase 1 alpha 1	NM_146087	D-062865-01	M-062865-00
1	E16	69	Casein kinase 1 delta	NM_027874	D-044377-01	M-044377-00
1	E17	83	Casein kinase 1 epsilon	NM_013767	D-040108-01	M-040108-00

1	E18	72	Casein kinase 1 gamma 1	NM_173185	D-055555-01	M-055555-00
1	E19	77	Casein kinase 1 gamma 2	NM_134002	D-040465-01	M-040465-00
1	E20	108	Casein kinase 1 gamma 3	NM_152809	D-064761-01	M-064761-00
1	E21	88	Casein kinase 2 alpha 1 polypeptide	NM_007788	D-058653-01	M-058653-00
1	E22	69	Casein kinase 2 alpha prime polypeptide	NM_009974	D-051582-01	M-051582-00
1	F03	78	Adrenergic receptor alpha 1a	NM_013461	D-059194-01	M-059194-00
1	F04	98	Adrenergic receptor alpha 1b	NM_007416	D-043437-01	M-043437-00
1	F05	87	Adrenergic receptor beta 2	NM_007420	D-042042-01	M-042042-00
1	F06	75	Adrenergic receptor kinase beta 1	NM_130863	D-040967-01	M-040967-00
1	F07	81	Adrenergic receptor kinase beta 2	NM_177078	D-051179-01	M-051179-00
1	F08	73	Angiopoietin 4	NM_009641	D-043390-01	M-043390-00
1	F09	85	Angiotensin II receptor type 2	NM_007429	D-061951-01	M-061951-00
1	F10	57	Xylulokinase homolog (H. Influenzae)	NM_001033209	D-062361-01	M-062361-00
1	F11	75	Protein kinase D2	NM_178900	D-040693-01	M-040693-00
1	F12	75	Serine/threonine/tyrosine kinase 1	NM_172891	D-055398-01	M-055398-00
1	F13	60	Casein kinase 2 beta polypeptide	NM_009975	D-049417-01	M-049417-00
1	F14	83	Chemokine (C-X-C motif) ligand 10	NM_021274	D-042605-01	M-042605-00
1	F15	78	SCY1-like 2 (S. Cerevisiae)	NM_198021	D-063381-01	M-063381-00
1	F16	67	Pyruvate dehydrogenase kinase isoenzyme 1	NM_172665	D-054066-01	M-054066-00
1	F17	67	Inositol hexaphosphate kinase 3	NM_173027	D-052483-01	M-052483-00
1	F18	65	Myosin light chain kinase 3	NM_175441	D-052765-01	M-052765-00
1	F19	66	DNA segment Chr 8 ERATO Doi 82 expressed	NM_172911	D-055423-01	M-055423-00
1	F20	81	Death associated protein kinase 1	NM_029653	D-040260-01	M-040260-00
1	F21	81	Death-associated protein kinase 2	NM_010019	D-042162-01	M-042162-00
1	F22	82	Death-associated protein kinase 3	NM_007828	D-044800-01	M-044800-00
1	G03	90	Mitogen-activated protein kinase-activated protein kinase 3	NM_178907	D-058176-01	M-058176-00
1	G04	90	Adenylate kinase 1	NM_021515	D-040896-01	M-040896-00
1	G05	84	Adenylate kinase 2	NM_016895	D-040897-01	M-040897-00
1	G06	69	Adenylate kinase 3	NM_021299	D-040898-01	M-040898-00
1	G07	80	Adenylate kinase 4	NM_009647	D-065422-01	M-065422-00
1	G08	83	A kinase (PRKA) anchor protein 1	NM_009648	D-042815-01	M-042815-00
1	G09	102	A kinase (PRKA) anchor protein 11	XM_127854	D-048015-01	M-048015-00
1	G10	58	A kinase (PRKA) anchor protein 13	XM_133543	D-059232-01	M-059232-00
1	G11	76	A kinase (PRKA) anchor protein 3	NM_009650	D-043482-01	M-043482-00
1	G12	86	A kinase (PRKA) anchor protein 4	NM_009651	D-043498-01	M-043498-00
1	G13	72	Doublecortin-like kinase 1	NM_019978	D-062033-01	M-062033-00
1	G14	81	Deoxycytidine kinase	NM_007832	D-055649-01	M-055649-00
1	G15	78	Discoidin domain receptor family member 1	NM_007584	D-040109-01	M-040109-00
1	G16	63	Discoidin domain receptor family member 2	NM_022563	D-047913-01	M-047913-00
1	G17	102	Diacylglycerol kinase alpha	NM_016811	D-041106-01	M-041106-00
1	G18	106	Diacylglycerol kinase beta	NM_178681	D-059281-01	M-059281-00
1	G19	77	Diacylglycerol kinase epsilon	NM_019505	D-049658-01	M-049658-00
1	G20	93	Diacylglycerol kinase gamma	NM_138650	D-042616-01	M-042616-00
1	G21	76	Diacylglycerol kinase theta	NM_199011	D-042776-01	M-042776-00
1	G22	86	Diacylglycerol kinase zeta	NM_138306	D-040327-01	M-040327-00
1	H03	97	A kinase (PRKA) anchor protein 8	NM_019774	D-060714-01	M-060714-00
1	H04	76	Thymoma viral proto-oncogene 1	NM_009652	D-040709-01	M-040709-00
1	H05	72	Thymoma viral proto-oncogene 2	NM_007434	D-040782-01	M-040782-00
1	H06	85	Thymoma viral proto-oncogene 3	NM_011785	D-040891-01	M-040891-00
1	H07	88	Anaplastic lymphoma kinase	NM_007439	D-040104-01	M-040104-00
1	H08	93	STE20-related kinase adaptor beta	NM_172656	D-042808-01	M-042808-00
1	H09	88	Anti-Mullerian hormone type 2 receptor	NM_144547	D-053605-01	M-053605-00
1	H10	74	Receptor-interacting serine-threonine kinase 4	NM_023663	D-048568-01	M-048568-00
1	H11	59	V-raf murine sarcoma 3611 viral oncogene homolog	NM_009703	D-042948-01	M-042948-00
1	H12	86	ADP-ribosylation factor-like 6 interacting protein 1	XM_133792	D-063637-01	M-063637-00
1	H13	86	Deoxyguanosine kinase	NM_013764	D-042284-01	M-042284-00
1	H14	91	Discs large homolog 1 (Drosophila)	NM_007862	D-042037-01	M-042037-00
1	H15	96	Discs large homolog 2 (Drosophila)	NM_011807	D-043520-01	M-043520-00

1	H16	90	Discs large homolog 3 (Drosophila)	NM_016747	D-062296-01	M-062296-00
1	H17	97	Discs large homolog 4 (Drosophila)	NM_007864	D-040099-01	M-040099-00
1	H18	81	Dystrophia myotonica-protein kinase	NM_032418	D-050244-01	M-050244-00
1	H19	98	Dnaj (Hsp40) homolog subfamily C member 3	NM_008929	D-058434-01	M-058434-00
1	H20	67	Docking protein 1	NM_010070	D-062538-01	M-062538-00
1	H21	70	Deoxythymidylate kinase	NM_023136	D-045682-01	M-045682-00
1	H22	67	Dual specificity phosphatase 1	NM_013642	D-040753-01	M-040753-00
1	I03	63	Ataxia telangiectasia mutated homolog (human)	NM_007499	D-042892-01	M-042892-00
1	I04	33	Aurora kinase B	NM_011496	D-063793-01	M-063793-00
1	I05	65	Aurora kinase C	NM_020572	D-050161-01	M-050161-00
1	I06	66	RIKEN cdna B230120H23 gene	NM_023057	D-045169-01	M-045169-00
1	I07	65	Ropporin 1-like	NM_145852	D-052427-01	M-052427-00
1	I08	94	Arginine vasopressin receptor 1A	NM_016847	D-044411-01	M-044411-00
1	I09	61	Arginine vasopressin receptor 1B	NM_011924	D-043452-01	M-043452-00
1	I10	71	Protein kinase membrane associated tyrosine/threonine 1	NM_023058	D-045252-01	M-045252-00
1	I11	77	Alpha-kinase 3	NM_054085	D-047437-01	M-047437-00
1	I12	57	Threonine synthase-like 1 (bacterial)	NM_001001297	D-057298-01	M-057298-00
1	I13	74	Dual specificity phosphatase 10	NM_022019	D-060807-01	M-060807-00
1	I14	65	Dual specificity phosphatase 2	NM_010090	D-040331-01	M-040331-00
1	I15	77	Dual specificity phosphatase 4	NM_176933	D-061306-01	M-061306-00
1	I16	66	Dual specificity phosphatase 6	NM_026268	D-040050-01	M-040050-00
1	I17	64	Dual specificity phosphatase 7	NM_153459	D-040051-01	M-040051-00
1	I18	74	Dual specificity phosphatase 8	NM_008748	D-048028-01	M-048028-00
1	I19	95	Dual-specificity tyrosine-(Y)-phosphorylation regulated kinase 1a	NM_007890	D-040110-01	M-040110-00
1	I20	74	Dual-specificity tyrosine-(Y)-phosphorylation regulated kinase 1b	NM_010092	D-041083-01	M-041083-00
1	I21	81	Dual-specificity tyrosine-(Y)-phosphorylation regulated kinase 3	NM_145508	D-053268-01	M-053268-00
1	I22	91	Dual-specificity tyrosine-(Y)-phosphorylation regulated kinase 4	NM_207210	D-057308-01	M-057308-00
1	J03	72	Protein serine kinase H1	NM_173432	D-058302-01	M-058302-00
1	J04	77	Protein kinase domain containing cytoplasmic	NM_134117	D-055034-01	M-055034-00
1	J05	99	AXL receptor tyrosine kinase	NM_009465	D-040941-01	M-040941-00
1	J06	50	Ecotropic viral integration site 5 like	NM_153536	D-054740-01	M-054740-00
1	J07	80	NUAK family SNF1-like kinase 1	XM_196007	D-063024-01	M-063024-00
1	J08	80	TAF1 RNA polymerase II TATA box binding protein (TBP)-associated factor	XM_194622	D-058598-01	M-058598-00
1	J09	74	NIMA (never in mitosis gene a)-related expressed kinase 5	NM_177898	D-061319-01	M-061319-00
1	J10	71	NAD kinase	NM_138671	D-053672-01	M-053672-00
1	J11	65	Nuclear receptor binding protein 2	NM_144847	D-051705-01	M-051705-00
1	J12	64	Cam kinase-like vesicle-associated	NM_145621	D-052770-01	M-052770-00
1	J13	85	RIKEN cdna E130304F04 gene	NM_175538	D-056574-01	M-056574-00
1	J14	63	Endothelin 2	NM_007902	D-056620-01	M-056620-00
1	J15	73	Eukaryotic elongation factor-2 kinase	NM_007908	D-060315-01	M-060315-00
1	J16	71	Epidermal growth factor receptor	NM_007912	D-040411-01	M-040411-00
1	J17	79	Eukaryotic translation initiation factor 2 alpha kinase 1	NM_013557	D-045523-01	M-045523-00
1	J18	64	Eukaryotic translation initiation factor 2 alpha kinase 3	NM_010121	D-044901-01	M-044901-00
1	J19	79	Eph receptor A1	NM_023580	D-063644-01	M-063644-00
1	J20	83	Eph receptor A2	NM_010139	D-040412-01	M-040412-00
1	J21	98	Eph receptor A3	NM_010140	D-043335-01	M-043335-00
1	J22	78	Eph receptor A4	NM_007936	D-055030-01	M-055030-00
1	K03	66	Regulation of nuclear pre-mrna domain containing 1A	NM_144861	D-051920-01	M-051920-00
1	K04	69	Cdna sequence BC021891	NM_145608	D-052680-01	M-052680-00
1	K05	63	Dihydroxyacetone kinase 2 homolog (yeast)	NM_145496	D-051984-01	M-051984-00
1	K06	56	PDLIM1 interacting kinase 1 like	NM_146156	D-054631-01	M-054631-00
1	K07	73	Fructosamine 3 kinase related protein	NM_181420	D-053981-01	M-053981-00
1	K08	76	CDC42 binding protein kinase gamma (DMPK-like)	XM_140553	D-056686-01	M-056686-00

1	K09	70	Obscurin cytoskeletal calmodulin and titin-interacting rhogef	NM_001003914	D-063726-01	M-063726-00
1	K10	63	Doublecortin-like kinase 3	NM_172928	D-052448-01	M-052448-00
1	K11	76	Branched chain ketoacid dehydrogenase kinase	NM_009739	D-061516-01	M-061516-00
1	K12	71	Breakpoint cluster region	XM_125706	D-040584-01	M-040584-00
1	K13	93	Eph receptor A7	NM_010141	D-040413-01	M-040413-00
1	K14	68	Eph receptor A8	NM_007939	D-045134-01	M-045134-00
1	K15	79	Eph receptor B1	NM_173447	D-057003-01	M-057003-00
1	K16	77	Eph receptor B2	NM_010142	D-050820-01	M-050820-00
1	K17	78	Eph receptor B3	NM_010143	D-043340-01	M-043340-00
1	K18	81	Eph receptor B4	NM_010144	D-060469-01	M-060469-00
1	K19	108	Eph receptor B6	NM_007680	D-040414-01	M-040414-00
1	K20	75	V-erb-b2 erythroblastic leukemia viral oncogene homolog 2 neuro/glioblastoma derived oncogene homolog (avian)	XM_109715	D-064147-01	M-064147-00
1	K21	71	V-erb-b2 erythroblastic leukemia viral oncogene homolog 3 (avian)	XM_125954	D-040415-01	M-040415-00
1	K22	87	V-erb-a erythroblastic leukemia viral oncogene homolog 4 (avian)	XM_136682	D-043436-01	M-043436-00
1	L03	84	Bradykinin receptor beta 2	NM_009747	D-060441-01	M-060441-00
1	L04	83	B lymphoid kinase	NM_007549	D-061983-01	M-061983-00
1	L05	61	B cell linker	NM_008528	D-040345-01	M-040345-00
1	L06	73	BMP2 inducible kinase	NM_080708	D-040720-01	M-040720-00
1	L07	86	Bone morphogenetic protein receptor type 1A	NM_009758	D-040598-01	M-040598-00
1	L08	75	Bone morphogenetic protein receptor type 1B	NM_007560	D-051071-01	M-051071-00
1	L09	68	Bone morphogenic protein receptor type II (serine/threonine kinase)	NM_007561	D-040599-01	M-040599-00
1	L10	90	BMX non-receptor tyrosine kinase	NM_009759	D-062471-01	M-062471-00
1	L11	94	Braf transforming gene	XM_355754	D-040325-01	M-040325-00
1	L12	87	Bromodomain containing 2	NM_010238	D-043404-01	M-043404-00
1	L13	81	Endoplasmic reticulum (ER) to nucleus signalling 1	NM_023913	D-041030-01	M-041030-00
1	L14	78	Ethanolamine kinase 1	XM_284250	D-057481-01	M-057481-00
1	L15	84	Ethanolamine kinase 2	NM_175443	D-063930-01	M-063930-00
1	L16	83	MDS1 and EVI1 complex locus	NM_007963	D-045212-01	M-045212-00
1	L17	74	Exosome component 10	NM_016699	D-049286-01	M-049286-00
1	L18	62	Fas-activated serine/threonine kinase	NM_023229	D-048817-01	M-048817-00
1	L19	81	Feline sarcoma oncogene	NM_010194	D-043381-01	M-043381-00
1	L20	79	Fibroblast growth factor receptor 1	NM_010206	D-040832-01	M-040832-00
1	L21	78	Fibroblast growth factor receptor 2	NM_201601	D-040288-01	M-040288-00
1	L22	76	Fibroblast growth factor receptor 3	NM_008010	D-065558-01	M-065558-00
1	M03	76	Bromodomain testis-specific	NM_054054	D-045525-01	M-045525-00
1	M04	80	Bruton agammaglobulinemia tyrosine kinase	NM_013482	D-040985-01	M-040985-00
1	M05	66	Budding uninhibited by benzimidazoles 1 homolog (S. Cerevisiae)	NM_009772	D-042998-01	M-042998-00
1	M06	84	Budding uninhibited by benzimidazoles 1 homolog beta (S. Cerevisiae)	NM_009773	D-044095-01	M-044095-00
1	M07	63	T cell receptor associated transmembrane adaptor 1	NM_198297	D-041911-01	M-041911-00
1	M08	76	Diacylglycerol kinase iota	XM_284192	D-055942-01	M-055942-00
1	M09	88	Protein kinase AMP-activated alpha 1 catalytic subunit	XM_139298	D-041035-01	M-041035-00
1	M10	59	RIKEN cdna C230081A13 gene	NM_172924	D-055433-01	M-055433-00
1	M11	68	6-phosphofructo-2-kinase/fructose-26-biphosphatase 4	NM_173019	D-054640-01	M-054640-00
1	M12	59	Phosphoinositide-3-kinase class 2 beta polypeptide	XM_136225	D-044033-01	M-044033-00
1	M13	85	Fibroblast growth factor receptor 4	NM_008011	D-045345-01	M-045345-00
1	M14	82	Gardner-Rasheed feline sarcoma viral (Fgr) oncogene homolog	NM_010208	D-041086-01	M-041086-00
1	M15	72	FMS-like tyrosine kinase 1	NM_010228	D-040636-01	M-040636-00
1	M16	84	FMS-like tyrosine kinase 3	NM_010229	D-040111-01	M-040111-00
1	M17	70	FMS-like tyrosine kinase 4	NM_008029	D-045433-01	M-045433-00
1	M18	79	Fructosamine 3 kinase	NM_022014	D-050096-01	M-050096-00

1	M19	77	Mechanistic target of rapamycin (serine/threonine kinase)	NM_020009	D-065427-01	M-065427-00
1	M20	92	Fyn-related kinase	NM_010237	D-059001-01	M-059001-00
1	M21	87	Fucokinase	NM_172283	D-064057-01	M-064057-00
1	M22	87	FYN binding protein	NM_011815	D-043701-01	M-043701-00
1	N03	69	Calmodulin 3	NM_007590	D-065395-01	M-065395-00
1	N04	87	Calcium/calmodulin-dependent protein kinase I	NM_133926	D-040624-01	M-040624-00
1	N05	79	Calcium/calmodulin-dependent protein kinase ID	NM_177343	D-063690-01	M-063690-00
1	N06	77	Calcium/calmodulin-dependent protein kinase I gamma	NM_144817	D-053402-01	M-053402-00
1	N07	82	Calcium/calmodulin-dependent protein kinase II alpha	NM_009792	D-059173-01	M-059173-00
1	N08	87	Calcium/calmodulin-dependent protein kinase II beta	NM_007595	D-062002-01	M-062002-00
1	N09	83	Calcium/calmodulin-dependent protein kinase II delta	NM_023813	D-040821-01	M-040821-00
1	N10	75	Calcium/calmodulin-dependent protein kinase IV	NM_009793	D-043023-01	M-043023-00
1	N11	83	Calcium/calmodulin-dependent protein kinase kinase 1 alpha	NM_018883	D-049735-01	M-049735-00
1	N12	65	Calcium/calmodulin-dependent protein kinase kinase 2 beta	NM_145358	D-040625-01	M-040625-00
1	N13	91	Fyn proto-oncogene	NM_008054	D-040112-01	M-040112-00
1	N14	71	Cyclin G associated kinase	NM_153569	D-052494-01	M-052494-00
1	N15	92	Galactokinase 1	NM_016905	D-044069-01	M-044069-00
1	N16	81	Galactokinase 2	NM_175154	D-056130-01	M-056130-00
1	N17	85	Growth associated protein 43	NM_008083	D-042499-01	M-042499-00
1	N18	66	Glucokinase	NM_010292	D-040844-01	M-040844-00
1	N19	75	Glial cell line derived neurotrophic factor family receptor alpha 2	NM_008115	D-045746-01	M-045746-00
1	N20	87	Glycerol kinase 2	NM_010294	D-065424-01	M-065424-00
1	N21	75	Predicted gene 711	XM_205054	D-058364-01	M-058364-00
1	N22	91	Glia maturation factor beta	NM_022023	D-050170-01	M-050170-00
1	O03	60	Caspase recruitment domain family member 10	NM_130859	D-050635-01	M-050635-00
1	O04	58	Caspase recruitment domain family member 14	NM_130886	D-063872-01	M-063872-00
1	O05	65	Sedoheptulokinase	NM_029031	D-042391-01	M-042391-00
1	O06	84	Calcium/calmodulin-dependent serine protein kinase (MAGUK family)	NM_009806	D-048887-01	M-048887-00
1	O07	63	Chemokine (C-C motif) ligand 12	NM_011331	D-043193-01	M-043193-00
1	O08	89	Chemokine (C-C motif) ligand 4	NM_013652	D-047365-01	M-047365-00
1	O09	83	Cyclin-dependent kinase 20	NM_053180	D-053874-01	M-053874-00
1	O10	67	CD3 antigen epsilon polypeptide	NM_007648	D-056107-01	M-056107-00
1	O11	77	CD4 antigen	NM_013488	D-049309-01	M-049309-00
1	O12	77	CD7 antigen	NM_009854	D-043076-01	M-043076-00
1	O13	71	Glia maturation factor gamma	NM_022024	D-050175-01	M-050175-00
1	O14	73	Glucosamine (UDP-N-acetyl)-2-epimerase/N-acetylmannosamine kinase	NM_015828	D-062937-01	M-062937-00
1	O15	89	G protein-coupled receptor kinase 4	NM_019497	D-040342-01	M-040342-00
1	O16	69	G protein-coupled receptor kinase 5	NM_018869	D-040343-01	M-040343-00
1	O17	95	G protein-coupled receptor kinase 1	NM_011881	D-040341-01	M-040341-00
1	O18	79	Protein phosphatase 1 regulatory subunit 17	NM_011153	D-061575-01	M-061575-00
1	O19	97	Germ cell-specific gene 2	NM_010353	D-048815-01	M-048815-00
1	O20	90	Glycogen synthase kinase 3 beta	NM_019827	D-041080-01	M-041080-00
1	O21	89	General transcription factor II H polypeptide 1	NM_008186	D-062142-01	M-062142-00
1	O22	98	Guanylate cyclase 2c	XM_132928	D-057453-01	M-057453-00
1	P03	62	Cytidine and dcmp deaminase domain containing 1	XM_127813	D-047908-01	M-047908-00
1	P04	75	Cyclin-dependent kinase 1	NM_007659	D-058633-01	M-058633-00
1	P05	92	Cyclin-dependent kinase 11B	NM_007661	D-040105-01	M-040105-00
1	P06	86	Cyclin-dependent kinase 13	XM_127221	D-045210-01	M-045210-00
1	P07	77	CDC42 binding protein kinase beta	NM_183016	D-041756-01	M-041756-00
1	P08	83	Cell division cycle 7 (S. Cerevisiae)	NM_009863	D-043093-01	M-043093-00
1	P09	81	Cyclin-dependent kinase 10	NM_194444	D-064531-01	M-064531-00

1	P10	90	Cyclin-dependent kinase 4	NM_009870	D-040106-01	M-040106-00
1	P11	89	Cyclin-dependent kinase 5	NM_007668	D-040544-01	M-040544-00
1	P12	95	Cyclin-dependent kinase 5 regulatory subunit 1 (p35)	NM_009871	D-041971-01	M-041971-00
1	P13	77	Guanylate cyclase 2e	NM_008192	D-046044-01	M-046044-00
1	P14	71	Guanylate cyclase 2f	XM_142224	D-059040-01	M-059040-00
1	P15	79	Glycerol kinase	NM_008194	D-065587-01	M-065587-00
1	P16	76	Alpha-kinase 2	XM_128981	D-056166-01	M-056166-00
1	P17	72	Hemopoietic cell kinase	NM_010407	D-040986-01	M-040986-00
1	P18	78	Homeodomain interacting protein kinase 1	NM_010432	D-040113-01	M-040113-00
1	P19	83	Homeodomain interacting protein kinase 2	NM_010433	D-040114-01	M-040114-00
1	P20	76	Homeodomain interacting protein kinase 3	NM_010434	D-041001-01	M-041001-00
1	P21	84	Hexokinase 1	NM_010438	D-043574-01	M-043574-00
1	P22	76	Leucine-rich repeat kinase 2	NM_025730	D-049666-01	M-049666-00
2	A03	74	Hexokinase 2	NM_013820	D-051128-01	M-051128-00
2	A04	82	Hexokinase 3	NM_001033245	D-045823-01	M-045823-00
2	A05	66	Heat shock protein 8	NM_030704	D-055174-01	M-055174-00
2	A06	77	Hormonally upregulated Neu-associated kinase	NM_015755	D-046705-01	M-046705-00
2	A07	114	Intestinal cell kinase	NM_019987	D-049932-01	M-049932-00
2	A08	97	Insulin-like growth factor I receptor	NM_010513	D-056843-01	M-056843-00
2	A09	101	Inositol hexaphosphate kinase 1	NM_013785	D-041917-01	M-041917-00
2	A10	89	Inhibitor of kappa light polypeptide enhancer in B cells kinase complex-associated protein	NM_026079	D-050005-01	M-050005-00
2	A11	98	Inhibitor of kappab kinase beta	NM_010546	D-040630-01	M-040630-00
2	A12	80	Inhibitor of kappab kinase epsilon	NM_019777	D-040798-01	M-040798-00
2	A13	80	Pantothenate kinase 4	NM_172990	D-055475-01	M-055475-00
2	A14	84	3'-phosphoadenosine 5'-phosphosulfate synthase 1	NM_011863	D-045089-01	M-045089-00
2	A15	101	3'-phosphoadenosine 5'-phosphosulfate synthase 2	NM_011864	D-044941-01	M-044941-00
2	A16	84	PAS domain containing serine/threonine kinase	NM_080850	D-065533-01	M-065533-00
2	A17	102	PDZ binding kinase	NM_023209	D-055354-01	M-055354-00
2	A18	107	Phosphoenolpyruvate carboxykinase 1 cytosolic	NM_011044	D-048453-01	M-048453-00
2	A19	83	Cyclin-dependent kinase 16	NM_011049	D-040144-01	M-040144-00
2	A20	83	Cyclin-dependent kinase 17	NM_146239	D-051660-01	M-051660-00
2	A21	70	Cyclin-dependent kinase 18	NM_008795	D-040145-01	M-040145-00
2	A22	73	Platelet derived growth factor receptor alpha polypeptide	NM_011058	D-048730-01	M-048730-00
2	B03	75	Interleukin 2	NM_008366	D-061458-01	M-061458-00
2	B04	83	Integrin linked kinase	NM_010562	D-040115-01	M-040115-00
2	B05	90	Inositol polyphosphate multikinase	XM_125641	D-062885-01	M-062885-00
2	B06	91	Insulin receptor	NM_010568	D-043748-01	M-043748-00
2	B07	87	Insulin receptor-related receptor	NM_011832	D-047791-01	M-047791-00
2	B08	98	Interleukin-1 receptor-associated kinase 1	NM_008363	D-040116-01	M-040116-00
2	B09	75	Interleukin-1 receptor-associated kinase 2	NM_172161	D-060091-01	M-060091-00
2	B10	88	Interleukin-1 receptor-associated kinase 3	NM_028679	D-059683-01	M-059683-00
2	B11	85	Insulin receptor substrate 1	NM_010570	D-040503-01	M-040503-00
2	B12	86	Integrin beta 1 binding protein 1	NM_008403	D-060350-01	M-060350-00
2	B13	79	Platelet derived growth factor receptor beta polypeptide	NM_008809	D-048218-01	M-048218-00
2	B14	101	Pyruvate dehydrogenase kinase isoenzyme 2	NM_133667	D-051566-01	M-051566-00
2	B15	86	Pyruvate dehydrogenase kinase isoenzyme 3	NM_145630	D-052920-01	M-052920-00
2	B16	106	Pyruvate dehydrogenase kinase isoenzyme 4	NM_013743	D-043425-01	M-043425-00
2	B17	78	3-phosphoinositide dependent protein kinase 1	NM_011062	D-040658-01	M-040658-00
2	B18	75	Pyridoxal (pyridoxine vitamin B6) kinase	NM_172134	D-054035-01	M-054035-00
2	B19	97	6-phosphofructo-2-kinase/fructose-26-biphosphatase 2	NM_008825	D-041829-01	M-041829-00
2	B20	92	6-phosphofructo-2-kinase/fructose-26-biphosphatase 3	NM_133232	D-050027-01	M-050027-00
2	B21	82	Phosphofructokinase liver B-type	NM_008826	D-060388-01	M-060388-00
2	B22	81	Phosphofructokinase muscle	NM_021514	D-065512-01	M-065512-00

2	C03	97	IL2 inducible T cell kinase	NM_010583	D-040679-01	M-040679-00
2	C04	76	Inositol 134-triphosphate 5/6 kinase	NM_172584	D-055096-01	M-055096-00
2	C05	84	Inositol 145-trisphosphate 3-kinase A	NM_146125	D-062876-01	M-062876-00
2	C06	77	Inositol 145-trisphosphate 3-kinase B	XM_205854	D-041024-01	M-041024-00
2	C07	68	Inositol 145-trisphosphate 3-kinase C	NM_181593	D-041107-01	M-041107-00
2	C08	87	Janus kinase 1	NM_146145	D-040117-01	M-040117-00
2	C09	93	Janus kinase 2	NM_008413	D-040118-01	M-040118-00
2	C10	88	Janus kinase 3	NM_010589	D-040119-01	M-040119-00
2	C11	95	Kinase insert domain protein receptor	NM_010612	D-040634-01	M-040634-00
2	C12	80	Ketohexokinase	NM_008439	D-062217-01	M-062217-00
2	C13	64	Phosphofructokinase platelet	NM_019703	D-059341-01	M-059341-00
2	C14	77	Cyclin-dependent kinase 14	NM_011074	D-040146-01	M-040146-00
2	C15	81	Phosphoglycerate kinase 1	NM_008828	D-040302-01	M-040302-00
2	C16	91	Phosphoglycerate kinase 2	NM_031190	D-043374-01	M-043374-00
2	C17	79	Phosphorylase kinase alpha 1	NM_008832	D-048299-01	M-048299-00
2	C18	97	Phosphorylase kinase alpha 2	NM_172783	D-055256-01	M-055256-00
2	C19	96	Phosphorylase kinase gamma 1	NM_011079	D-043266-01	M-043266-00
2	C20	88	Phosphorylase kinase gamma 2 (testis)	NM_026888	D-059847-01	M-059847-00
2	C21	75	Phosphatidylinositol 4-kinase type 2 alpha	NM_145501	D-065311-01	M-065311-00
2	C22	74	Phosphatidylinositol 4-kinase type 2 beta	NM_025951	D-065308-01	M-065308-00
2	D03	84	Kinesin family member 13B	XM_283218	D-047951-01	M-047951-00
2	D04	64	U2AF homology motif (UHM) kinase 1	NM_010633	D-041701-01	M-041701-00
2	D05	86	Kit oncogene	NM_021099	D-042174-01	M-042174-00
2	D06	79	Large tumor suppressor 2	NM_015771	D-044602-01	M-044602-00
2	D07	62	Lymphocyte protein tyrosine kinase	NM_010693	D-043878-01	M-043878-00
2	D08	87	Lymphocyte cytosolic protein 2	NM_010696	D-059042-01	M-059042-00
2	D09	85	LIM-domain containing protein kinase	NM_010717	D-043923-01	M-043923-00
2	D10	62	LIM motif-containing protein kinase 2	NM_010718	D-043932-01	M-043932-00
2	D11	79	Myosin light polypeptide kinase 2 skeletal muscle	XM_130630	D-046735-01	M-046735-00
2	D12	83	Leukocyte tyrosine kinase	NM_008523	D-063855-01	M-063855-00
2	D13	70	Phosphatidylinositol 3-kinase C2 domain containing alpha polypeptide	NM_011083	D-049318-01	M-049318-00
2	D14	68	Phosphatidylinositol 3-kinase C2 domain containing gamma polypeptide	NM_011084	D-065486-01	M-065486-00
2	D15	91	Phosphatidylinositol 3-kinase catalytic alpha polypeptide	NM_008839	D-040730-01	M-040730-00
2	D16	88	Phosphatidylinositol 3-kinase catalytic beta polypeptide	NM_029094	D-040088-01	M-040088-00
2	D17	89	Phosphoinositide-3-kinase catalytic gamma polypeptide	NM_020272	D-040929-01	M-040929-00
2	D18	86	Phosphatidylinositol 3-kinase regulatory subunit polypeptide 1 (p85 alpha)	NM_011085	D-041079-01	M-041079-00
2	D19	78	Phosphatidylinositol 3-kinase regulatory subunit polypeptide 2 (p85 beta)	NM_008841	D-041085-01	M-041085-00
2	D20	85	Phosphatidylinositol 3 kinase regulatory subunit polypeptide 3 (p55)	NM_181585	D-041300-01	M-041300-00
2	D21	85	Phosphatidylinositol 3 kinase regulatory subunit polypeptide 4 p150	XM_135116	D-062225-01	M-062225-00
2	D22	60	Phosphatidylinositol 4-kinase catalytic beta polypeptide	NM_175356	D-056390-01	M-056390-00
2	E03	70	Yamaguchi sarcoma viral (v-yes-1) oncogene homolog	NM_010747	D-040987-01	M-040987-00
2	E04	82	Male germ cell-associated kinase	NM_008547	D-047244-01	M-047244-00
2	E05	73	Mucosa associated lymphoid tissue lymphoma translocation gene 1	NM_172833	D-051221-01	M-051221-00
2	E06	79	Mitogen-activated protein kinase kinase 1	NM_008927	D-040605-01	M-040605-00
2	E07	85	Late endosomal/lysosomal adaptor MAPK and MTOR activator 3	NM_019920	D-049984-01	M-049984-00
2	E08	71	Mitogen-activated protein kinase kinase 2	NM_023138	D-040606-01	M-040606-00
2	E09	74	Mitogen-activated protein kinase kinase 3	NM_008928	D-040121-01	M-040121-00
2	E10	58	Mitogen-activated protein kinase kinase 4	NM_009157	D-040122-01	M-040122-00
2	E11	73	Mitogen-activated protein kinase kinase 5	NM_011840	D-040607-01	M-040607-00
2	E12	82	Mitogen-activated protein kinase kinase 6	NM_011943	D-043421-01	M-043421-00
2	E13	82	Proviral integration site 1	NM_008842	D-040685-01	M-040685-00

2	E14	81	Proviral integration site 2	NM_138606	D-052724-01	M-052724-00
2	E15	75	Proviral integration site 3	NM_145478	D-052883-01	M-052883-00
2	E16	80	PTEN induced putative kinase 1	NM_026880	D-044666-01	M-044666-00
2	E17	69	Phosphatidylinositol-4-phosphate 5-kinase type 1 alpha	NM_008847	D-041183-01	M-041183-00
2	E18	87	Phosphatidylinositol-5-phosphate 4-kinase type II alpha	NM_008845	D-040163-01	M-040163-00
2	E19	77	Phosphatidylinositol-5-phosphate 4-kinase type II beta	NM_054051	D-054851-01	M-054851-00
2	E20	93	Phosphatidylinositol-5-phosphate 4-kinase type II gamma	NM_054097	D-040164-01	M-040164-00
2	E21	78	Phosphatidylinositol-4-phosphate 5-kinase-like 1	NM_198191	D-057347-01	M-057347-00
2	E22	97	Protein kinase inhibitor alpha	NM_008862	D-058432-01	M-058432-00
2	F03	83	Mitogen-activated protein kinase kinase 7	NM_011944	D-040610-01	M-040610-00
2	F04	85	Mitogen-activated protein kinase kinase kinase 1	NM_011945	D-041090-01	M-041090-00
2	F05	80	Mitogen-activated protein kinase kinase kinase 10	XM_194344	D-058190-01	M-058190-00
2	F06	82	Mitogen-activated protein kinase kinase kinase 11	NM_022012	D-040101-01	M-040101-00
2	F07	94	Mitogen-activated protein kinase kinase kinase 12	NM_009582	D-040608-01	M-040608-00
2	F08	72	Mitogen-activated protein kinase kinase kinase 14	NM_016896	D-040771-01	M-040771-00
2	F09	55	Mitogen-activated protein kinase kinase kinase 2	NM_011946	D-040920-01	M-040920-00
2	F10	81	Mitogen-activated protein kinase kinase kinase 3	NM_011947	D-040123-01	M-040123-00
2	F11	79	Mitogen-activated protein kinase kinase kinase 4	NM_011948	D-040609-01	M-040609-00
2	F12	106	Mitogen-activated protein kinase kinase kinase 5	NM_008580	D-041179-01	M-041179-00
2	F13	81	Protein kinase inhibitor beta camp dependent testis specific	NM_008863	D-058816-01	M-058816-00
2	F14	68	Pyruvate kinase liver and red blood cell	NM_013631	D-057382-01	M-057382-00
2	F15	64	Pyruvate kinase muscle	NM_011099	D-062711-01	M-062711-00
2	F16	78	Protein kinase N1	NM_177262	D-065212-01	M-065212-00
2	F17	82	Protein kinase N2	NM_178654	D-065210-01	M-065210-00
2	F18	69	Protein kinase N3	NM_153805	D-065208-01	M-065208-00
2	F19	25	Polo-like kinase 1 (Drosophila)	NM_011121	D-040566-01	M-040566-00
2	F20	50	Polo-like kinase 2 (Drosophila)	NM_152804	D-040151-01	M-040151-00
2	F21	79	Polo-like kinase 3 (Drosophila)	NM_013807	D-051404-01	M-051404-00
2	F22	85	Polo-like kinase 4 (Drosophila)	NM_011495	D-051400-01	M-051400-00
2	G03	94	Mitogen-activated protein kinase kinase kinase 6	NM_016693	D-043402-01	M-043402-00
2	G04	87	Mitogen-activated protein kinase kinase kinase 7	NM_172688	D-040718-01	M-040718-00
2	G05	88	Mitogen-activated protein kinase kinase kinase 8	NM_007746	D-040683-01	M-040683-00
2	G06	81	Mitogen-activated protein kinase kinase kinase 9	NM_177395	D-057199-01	M-057199-00
2	G07	85	Mitogen-activated protein kinase kinase kinase kinase 1	NM_008279	D-042597-01	M-042597-00
2	G08	62	Mitogen-activated protein kinase kinase kinase kinase 2	NM_009006	D-047980-01	M-047980-00
2	G09	105	Mitogen-activated protein kinase kinase kinase kinase 3	XM_128800	D-040611-01	M-040611-00
2	G10	78	Mitogen-activated protein kinase kinase kinase kinase 4	NM_008696	D-040100-01	M-040100-00
2	G11	83	Mitogen-activated protein kinase kinase kinase kinase 5	NM_024275	D-040124-01	M-040124-00
2	G12	90	Misshapen-like kinase 1 (zebrafish)	NM_016713	D-040612-01	M-040612-00
2	G13	66	Phosphomevalonate kinase	NM_026784	D-041051-01	M-041051-00
2	G14	83	Polynucleotide kinase 3'- phosphatase	NM_021549	D-048839-01	M-048839-00
2	G15	79	Protein phosphatase 1 regulatory (inhibitor) subunit 1B	NM_144828	D-040427-01	M-040427-00
2	G16	111	Protein phosphatase 2 (formerly 2A) catalytic subunit alpha isoform	NM_019411	D-040657-01	M-040657-00
2	G17	95	Protein phosphatase 2 (formerly 2A) catalytic subunit beta isoform	NM_017374	D-041905-01	M-041905-00

2	G18	82	Protein phosphatase 4 catalytic subunit	NM_019674	D-040058-01	M-040058-00
2	G19	87	Protein kinase AMP-activated alpha 2 catalytic subunit	XM_131633	D-040809-01	M-040809-00
2	G20	69	Protein kinase camp dependent catalytic alpha	NM_008854	D-047080-01	M-047080-00
2	G21	85	Protein kinase camp dependent catalytic beta	NM_011100	D-042579-01	M-042579-00
2	G22	91	Protein kinase AMP-activated gamma 1 non-catalytic subunit	NM_016781	D-048876-01	M-048876-00
2	H03	95	Mitogen-activated protein kinase 1	NM_011949	D-040613-01	M-040613-00
2	H04	96	Mitogen-activated protein kinase 10	NM_009158	D-045023-01	M-045023-00
2	H05	88	Mitogen-activated protein kinase 11	NM_011161	D-050928-01	M-050928-00
2	H06	77	Mitogen-activated protein kinase 12	NM_013871	D-062913-01	M-062913-00
2	H07	73	Mitogen-activated protein kinase 13	NM_011950	D-043774-01	M-043774-00
2	H08	71	Mitogen-activated protein kinase 14	NM_011951	D-040125-01	M-040125-00
2	H09	83	Mitogen-activated protein kinase 3	NM_011952	D-040126-01	M-040126-00
2	H10	83	Mitogen-activated protein kinase 4	NM_172632	D-055010-01	M-055010-00
2	H11	87	Mitogen-activated protein kinase 6	NM_015806	D-040133-01	M-040133-00
2	H12	92	Mitogen-activated protein kinase 7	NM_011841	D-040333-01	M-040333-00
2	H13	78	Protein kinase AMP-activated gamma 3 non-catatlytic subunit	NM_153744	D-057924-01	M-057924-00
2	H14	71	Protein kinase camp dependent regulatory type I alpha	NM_021880	D-040846-01	M-040846-00
2	H15	99	Protein kinase camp dependent regulatory type II alpha	NM_008924	D-042734-01	M-042734-00
2	H16	82	Protein kinase camp dependent regulatory type II beta	NM_011158	D-063792-01	M-063792-00
2	H17	87	Protein kinase C alpha	NM_011101	D-040348-01	M-040348-00
2	H18	105	Protein interacting with C kinase 1	NM_008837	D-048339-01	M-048339-00
2	H19	80	Protein kinase C beta	NM_008855	D-048412-01	M-048412-00
2	H20	88	Protein kinase C gamma	NM_011102	D-050293-01	M-050293-00
2	H21	86	Protein kinase C delta	NM_011103	D-040147-01	M-040147-00
2	H22	80	Protein kinase C epsilon	NM_011104	D-040148-01	M-040148-00
2	I03	95	Mitogen-activated protein kinase 8	NM_016700	D-040128-01	M-040128-00
2	I04	73	Mitogen-activated protein kinase 8 interacting protein 1	NM_011162	D-042191-01	M-042191-00
2	I05	84	Mitogen-activated protein kinase 8 interacting protein 2	NM_021921	D-061724-01	M-061724-00
2	I06	83	Mitogen-activated protein kinase 8 interacting protein 3	NM_013931	D-043334-01	M-043334-00
2	I07	79	Mitogen-activated protein kinase 9	NM_016961	D-040134-01	M-040134-00
2	I08	93	MAP kinase-activated protein kinase 2	NM_008551	D-040135-01	M-040135-00
2	I09	79	MAP kinase-activated protein kinase 5	NM_010765	D-040095-01	M-040095-00
2	I10	77	MAP/microtubule affinity-regulating kinase 1	NM_145515	D-053815-01	M-053815-00
2	I11	73	MAP/microtubule affinity-regulating kinase 2	NM_007928	D-040137-01	M-040137-00
2	I12	59	MAP/microtubule affinity-regulating kinase 3	NM_021516	D-040138-01	M-040138-00
2	I13	75	Protein kinase C eta	NM_008856	D-040158-01	M-040158-00
2	I14	71	Protein kinase C iota	NM_008857	D-040822-01	M-040822-00
2	I15	71	Protein kinase D1	NM_008858	D-048415-01	M-048415-00
2	I16	70	Protein kinase D3	NM_029239	D-040692-01	M-040692-00
2	I17	100	Protein kinase C theta	NM_008859	D-048426-01	M-048426-00
2	I18	68	Protein kinase C substrate 80K-H	NM_008925	D-048665-01	M-048665-00
2	I19	74	Protein kinase C zeta	NM_008860	D-040823-01	M-040823-00
2	I20	75	Protein kinase DNA activated catalytic polypeptide	NM_011159	D-040958-01	M-040958-00
2	I21	89	Protein kinase cgmp-dependent type I	NM_011160	D-048829-01	M-048829-00
2	I22	76	Protein kinase cgmp-dependent type II	NM_008926	D-062310-01	M-062310-00
2	J03	76	MAP/microtubule affinity-regulating kinase 4	NM_172279	D-054377-01	M-054377-00
2	J04	97	Microtubule associated serine/threonine kinase 2	NM_008641	D-040614-01	M-040614-00
2	J05	89	Microtubule associated serine/threonine kinase-like	NM_025979	D-063943-01	M-063943-00
2	J06	104	Megakaryocyte-associated tyrosine kinase	NM_010768	D-044002-01	M-044002-00
2	J07	89	MAP3K12 binding inhibitory protein 1	NM_145442	D-062812-01	M-062812-00
2	J08	69	Maternal embryonic leucine zipper kinase	NM_010790	D-059459-01	M-059459-00
2	J09	87	C-mer proto-oncogene tyrosine kinase	NM_008587	D-040357-01	M-040357-00
2	J10	71	Met proto-oncogene	NM_008591	D-040878-01	M-040878-00

2	J11	78	MAP kinase-interacting serine/threonine kinase 1	NM_021461	D-040139-01	M-040139-00
2	J12	86	MAP kinase-interacting serine/threonine kinase 2	NM_021462	D-041005-01	M-041005-00
2	J13	78	Eukaryotic translation initiation factor 2-alpha kinase 2	NM_011163	D-040807-01	M-040807-00
2	J14	81	Protein kinase interferon inducible double stranded RNA dependent activator	NM_011871	D-040713-01	M-040713-00
2	J15	89	WNK lysine deficient protein kinase 1	NM_198703	D-041543-01	M-041543-00
2	J16	80	WNK lysine deficient protein kinase 3 pseudogene	XM_205148	D-059069-01	M-059069-00
2	J17	76	WNK lysine deficient protein kinase 4	NM_175638	D-040229-01	M-040229-00
2	J18	88	Protein kinase X-linked	NM_016979	D-056757-01	M-056757-00
2	J19	68	PRP4 pre-mrna processing factor 4 homolog B (yeast)	NM_013830	D-051113-01	M-051113-00
2	J20	83	Phosphoribosyl pyrophosphate synthetase 1	NM_021463	D-063233-01	M-063233-00
2	J21	91	Phosphoribosyl pyrophosphate synthetase 2	NM_026662	D-057480-01	M-057480-00
2	J22	80	Phosphoribosyl pyrophosphate synthetase-associated protein 2	NM_144806	D-053452-01	M-053452-00
2	K03	64	Moloney sarcoma oncogene	NM_020021	D-049882-01	M-049882-00
2	K04	78	Membrane protein palmitoylated	NM_008621	D-062246-01	M-062246-00
2	K05	72	Membrane protein palmitoylated 2 (MAGUK p55 subfamily member 2)	NM_016695	D-040140-01	M-040140-00
2	K06	75	Membrane protein palmitoylated 3 (MAGUK p55 subfamily member 3)	NM_007863	D-044887-01	M-044887-00
2	K07	88	Myelin protein zero-like 1	XM_129565	D-044264-01	M-044264-00
2	K08	73	Mannose receptor C type 2	NM_008626	D-040940-01	M-040940-00
2	K09	92	Macrophage stimulating 1 receptor (c-met-related tyrosine kinase)	NM_009074	D-046427-01	M-046427-00
2	K10	75	Muscle skeletal receptor tyrosine kinase	NM_010944	D-046617-01	M-046617-00
2	K11	80	Mevalonate (diphospho) decarboxylase	NM_138656	D-053575-01	M-053575-00
2	K12	74	Mevalonate kinase	NM_023556	D-063629-01	M-063629-00
2	K13	73	PTK2 protein tyrosine kinase 2	NM_007982	D-041099-01	M-041099-00
2	K14	68	PTK2 protein tyrosine kinase 2 beta	NM_172498	D-040719-01	M-040719-00
2	K15	73	PTK6 protein tyrosine kinase 6	NM_009184	D-044700-01	M-044700-00
2	K16	80	PTK7 protein tyrosine kinase 7	NM_175168	D-040788-01	M-040788-00
2	K17	76	Twinfilin actin-binding protein homolog 1 (Drosophila)	NM_008971	D-060402-01	M-060402-00
2	K18	74	Twinfilin actin-binding protein homolog 2 (Drosophila)	NM_011876	D-062823-01	M-062823-00
2	K19	74	Protein tyrosine phosphatase non-receptor type 5	NM_013643	D-046680-01	M-046680-00
2	K20	82	Protein tyrosine phosphatase receptor type G	NM_008981	D-048271-01	M-048271-00
2	K21	82	Protein tyrosine phosphatase receptor type J	NM_008982	D-050291-01	M-050291-00
2	K22	92	Protein tyrosine phosphatase receptor type R	NM_011217	D-040062-01	M-040062-00
2	L03	85	Myosin light polypeptide kinase	NM_139300	D-041418-01	M-041418-00
2	L04	84	Myosin IIIA	NM_148413	D-054332-01	M-054332-00
2	L05	81	N-acetylglucosamine kinase	NM_019542	D-049645-01	M-049645-00
2	L06	85	NIMA (never in mitosis gene a)-related expressed kinase 11	NM_172461	D-056397-01	M-056397-00
2	L07	91	NIMA (never in mitosis gene a)-related expressed kinase 2	NM_010892	D-045662-01	M-045662-00
2	L08	89	NIMA (never in mitosis gene a)-related expressed kinase 3	NM_011848	D-045726-01	M-045726-00
2	L09	64	NIMA (never in mitosis gene a)-related expressed kinase 4	NM_011849	D-045512-01	M-045512-00
2	L10	86	NIMA (never in mitosis gene a)-related expressed kinase 6	NM_021606	D-059408-01	M-059408-00
2	L11	89	NIMA (never in mitosis gene a)-related expressed kinase 7	NM_021605	D-063266-01	M-063266-00
2	L12	82	NIMA (never in mitosis gene a)-related expressed kinase 8	NM_080849	D-044403-01	M-044403-00
2	L13	92	Protein tyrosine phosphatase receptor type T	NM_021464	D-048432-01	M-048432-00
2	L14	70	PX domain containing serine/threonine kinase	NM_145458	D-055422-01	M-055422-00
2	L15	77	Aldehyde dehydrogenase 18 family member A1	NM_019698	D-049794-01	M-049794-00
2	L16	81	RAS-related C3 botulinum substrate 1	NM_009007	D-041170-01	M-041170-00
2	L17	77	V-raf-leukemia viral oncogene 1	NM_029780	D-040149-01	M-040149-00

2	L18	95	Serine/threonine kinase 30	NM_011973	D-048657-01	M-048657-00
2	L19	81	Rap guanine nucleotide exchange factor (GEF) 3	NM_144850	D-057800-01	M-057800-00
2	L20	82	Rap guanine nucleotide exchange factor (GEF) 4	NM_019688	D-057784-01	M-057784-00
2	L21	91	RAS protein-specific guanine nucleotide-releasing factor 2	NM_009027	D-047527-01	M-047527-00
2	L22	62	Ribokinase	NM_153196	D-062934-01	M-062934-00
2	M03	77	NIMA (never in mitosis gene a)-related expressed kinase 9	NM_145138	D-040141-01	M-040141-00
2	M04	75	Nemo like kinase	NM_008702	D-042021-01	M-042021-00
2	M05	76	Non-metastatic cells 1 protein (NM23A) expressed in	NM_008704	D-040142-01	M-040142-00
2	M06	82	Non-metastatic cells 2 protein (NM23B) expressed in	NM_008705	D-040143-01	M-040143-00
2	M07	77	Non-metastatic cells 3 protein expressed in	NM_019730	D-049492-01	M-049492-00
2	M08	101	Non-metastatic cells 4 protein expressed in	NM_019731	D-049846-01	M-049846-00
2	M09	78	Non-metastatic cells 5 protein expressed in (nucleoside-diphosphate kinase)	NM_080637	D-049199-01	M-049199-00
2	M10	95	Non-metastatic cells 6 protein expressed in (nucleoside-diphosphate kinase)	NM_018757	D-045821-01	M-045821-00
2	M11	90	Non-metastatic cells 7 protein expressed in (nucleoside-diphosphate kinase)	NM_138314	D-053365-01	M-053365-00
2	M12	76	Natriuretic peptide receptor 1	NM_008727	D-042648-01	M-042648-00
2	M13	63	Ret proto-oncogene	NM_009050	D-047013-01	M-047013-00
2	M14	74	Riboflavin kinase	NM_019437	D-046077-01	M-046077-00
2	M15	82	RIO kinase 1 (yeast)	NM_024242	D-056893-01	M-056893-00
2	M16	88	RIO kinase 3 (yeast)	NM_024182	D-050639-01	M-050639-00
2	M17	79	Receptor (TNFRSF)-interacting serine-threonine kinase 1	NM_009068	D-040150-01	M-040150-00
2	M18	74	Receptor (TNFRSF)-interacting serine-threonine kinase 2	NM_138952	D-052248-01	M-052248-00
2	M19	100	Receptor-interacting serine-threonine kinase 3	NM_019955	D-049919-01	M-049919-00
2	M20	84	Ribonuclease L (2' 5'-oligoisoadenylate synthetase-dependent)	NM_011882	D-043480-01	M-043480-00
2	M21	96	Rho-associated coiled-coil containing protein kinase 1	NM_009071	D-046504-01	M-046504-00
2	M22	94	Rho-associated coiled-coil containing protein kinase 2	NM_009072	D-040429-01	M-040429-00
2	N03	80	Natriuretic peptide receptor 2	NM_173788	D-050423-01	M-050423-00
2	N04	76	Nuclear receptor binding protein 1	NM_147201	D-054243-01	M-054243-00
2	N05	75	Neuregulin 3	NM_008734	D-047955-01	M-047955-00
2	N06	83	Nik related kinase	NM_013724	D-046446-01	M-046446-00
2	N07	71	Neurotrophic tyrosine kinase receptor type 1	XM_283871	D-049564-01	M-049564-00
2	N08	77	Neurotrophic tyrosine kinase receptor type 2	NM_008745	D-048017-01	M-048017-00
2	N09	84	Neurotrophic tyrosine kinase receptor type 3	NM_008746	D-041561-01	M-041561-00
2	N10	88	Oxidative-stress responsive 1	XM_135264	D-062357-01	M-062357-00
2	N11	79	Protein kinase C and casein kinase substrate in neurons 1	NM_011861	D-062209-01	M-062209-00
2	N12	80	Phosphoprotein associated with glycosphingolipid microdomains 1	NM_053182	D-055487-01	M-055487-00
2	N13	85	Receptor tyrosine kinase-like orphan receptor 1	NM_013845	D-053825-01	M-053825-00
2	N14	81	Receptor tyrosine kinase-like orphan receptor 2	NM_013846	D-041074-01	M-041074-00
2	N15	72	Ros1 proto-oncogene	NM_011282	D-042212-01	M-042212-00
2	N16	89	Retinitis pigmentosa 2 homolog (human)	NM_133669	D-042964-01	M-042964-00
2	N17	79	Ribosomal protein S6 kinase polypeptide 1	NM_009097	D-045777-01	M-045777-00
2	N18	67	Ribosomal protein S6 kinase polypeptide 2	NM_011299	D-042672-01	M-042672-00
2	N19	85	Ribosomal protein S6 kinase polypeptide 3	NM_148945	D-054432-01	M-054432-00
2	N20	87	Ribosomal protein S6 kinase polypeptide 5	NM_153587	D-040751-01	M-040751-00
2	N21	85	Ribosomal protein S6 kinase polypeptide 6	NM_025949	D-046138-01	M-046138-00
2	N22	77	Ribosomal protein S6 kinase polypeptide 2	NM_021485	D-040894-01	M-040894-00
2	O03	81	P21 protein (Cdc42/Rac)-activated kinase 1	NM_011035	D-048101-01	M-048101-00
2	O04	83	P21 protein (Cdc42/Rac)-activated kinase 2	NM_177326	D-040615-01	M-040615-00
2	O05	79	P21 protein (Cdc42/Rac)-activated kinase 3	NM_008778	D-040616-01	M-040616-00
2	O06	60	P21 protein (Cdc42/Rac)-activated kinase 4	NM_027470	D-059484-01	M-059484-00

2	O07	69	P21 protein (Cdc42/Rac)-activated kinase 6	XM_111790	D-047118-01	M-047118-00
2	O08	86	P21 protein (Cdc42/Rac)-activated kinase 7	NM_172858	D-055362-01	M-055362-00
2	O09	99	Pantothenate kinase 1	NM_023792	D-042165-01	M-042165-00
2	O10	88	Pantothenate kinase 3	NM_145962	D-051419-01	M-051419-00
2	O11	94	Yeast Sps1/Ste20-related kinase 4 (S. Cerevisiae)	XM_136210	D-044094-01	M-044094-00
2	O12	90	Adenylate kinase domain containing 1	XM_137065	D-063402-01	M-063402-00
2	O13	68	Ribosomal protein S6 kinase-like 1	NM_146244	D-057689-01	M-057689-00
2	O14	73	Receptor-like tyrosine kinase	XM_135104	D-064592-01	M-064592-00
2	O15	84	Microtubule associated serine/threonine kinase 1	NM_019945	D-055903-01	M-055903-00
2	O16	77	SCY1-like 1 (S. Cerevisiae)	NM_023912	D-047892-01	M-047892-00
2	O17	83	Selenophosphate synthetase 1	NM_175400	D-057100-01	M-057100-00
2	O18	85	Selenophosphate synthetase 2	NM_009266	D-050751-01	M-050751-00
2	O19	85	Serum/glucocorticoid regulated kinase 1	NM_011361	D-040774-01	M-040774-00
2	O20	92	Serum/glucocorticoid regulated kinase 2	NM_013731	D-047584-01	M-047584-00
2	O21	105	Serum/glucocorticoid regulated kinase 3	NM_133220	D-050771-01	M-050771-00
2	O22	68	Src homology 2 domain-containing transforming protein C1	NM_011368	D-040793-01	M-040793-00
2	P03	80	Homeodomain interacting protein kinase 4	XM_133316	D-058179-01	M-058179-00
2	P04	91	A kinase (PRKA) anchor protein 5	XM_138063	D-043936-01	M-043936-00
2	P05	81	Dual specificity phosphatase 5	XM_140740	D-057231-01	M-057231-00
2	P06	97	Ataxia telangiectasia and Rad3 related	XM_147046	D-062167-01	M-062167-00
2	P07	68	Cyclin-dependent kinase 15	XM_194683	D-043393-01	M-043393-00
2	P08	90	Src family associated phosphoprotein 1	XM_203404	D-064190-01	M-064190-00
2	P09	94	Kinase suppressor of ras 2	XM_285897	D-054277-01	M-054277-00
2	P10	88	Cyclin-dependent kinase-like 4	XM_355031	D-055022-01	M-055022-00
2	P11	83	RIKEN cdna 2810408M09 gene	XM_355368	D-046643-01	M-046643-00
2	P12	94	NIMA (never in mitosis gene a)-related expressed kinase 1	XM_356077	D-061162-01	M-061162-00
2	P13	90	Salt inducible kinase 2	NM_178710	D-041008-01	M-041008-00
2	P14	76	MAD homolog 7 (Drosophila)	NM_008543	D-047242-01	M-047242-00
2	P15	88	Salt inducible kinase 1	NM_010831	D-044399-01	M-044399-00
2	P16	90	SNF related kinase	NM_133741	D-051065-01	M-051065-00
2	P17	86	Suppressor of cytokine signaling 1	NM_009896	D-043120-01	M-043120-00
2	P18	87	Suppressor of cytokine signaling 5	NM_019654	D-042294-01	M-042294-00
2	P19	87	Sperm autoantigenic protein 17	NM_011449	D-045569-01	M-045569-00
2	P20	87	Sphingosine kinase 1	NM_025367	D-040671-01	M-040671-00
2	P21	82	Sphingosine kinase 2	NM_020011	D-041258-01	M-041258-00
2	P22	85	Sequestosome 1	NM_011018	D-047628-01	M-047628-00
3	A03	83	Rous sarcoma oncogene	NM_009271	D-040877-01	M-040877-00
3	A04	86	Src-related kinase lacking C-terminal regulatory tyrosine and N-terminal myristylation sites	NM_011481	D-062777-01	M-062777-00
3	A05	78	Serine/arginine-rich protein specific kinase 1	NM_016795	D-061647-01	M-061647-00
3	A06	89	Serine/arginine-rich protein specific kinase 2	NM_009274	D-055142-01	M-055142-00
3	A07	79	Testis-specific serine kinase 6	NM_032004	D-046069-01	M-046069-00
3	A08	74	Serine/threonine kinase 10	NM_009288	D-044001-01	M-044001-00
3	A09	87	Serine/threonine kinase 11	NM_011492	D-044342-01	M-044342-00
3	A10	89	Serine/threonine kinase 16	NM_011494	D-040152-01	M-040152-00
3	A11	87	Serine/threonine kinase 17b (apoptosis-inducing)	NM_133810	D-040944-01	M-040944-00
3	A12	81	Serine/threonine kinase 19	NM_019442	D-063053-01	M-063053-00
3	B03	87	STE20-like kinase (yeast)	NM_009289	D-040579-01	M-040579-00
3	B04	86	Testis-specific serine kinase 1	NM_009435	D-042335-01	M-042335-00
3	B05	78	Testis-specific serine kinase 2	NM_009436	D-051637-01	M-051637-00
3	B06	90	Testis-specific serine kinase 3	NM_080442	D-049881-01	M-049881-00
3	B07	87	Testis-specific serine kinase substrate	NM_011651	D-043849-01	M-043849-00
3	B08	85	Serine/arginine-rich protein specific kinase 3	NM_019684	D-049832-01	M-049832-00
3	B09	73	Serine/threonine kinase 24 (STE20 homolog yeast)	NM_145465	D-062796-01	M-062796-00
3	B10	72	Serine/threonine kinase 25 (yeast)	NM_021537	D-065319-01	M-065319-00

3	B11	85	Serine/threonine kinase 3 (Ste20 yeast homolog)	NM_019635	D-040440-01	M-040440-00
3	B12	84	Serine threonine kinase 31	NM_029916	D-046426-01	M-046426-00
3	C03	67	Serine/threonine kinase 32B	NM_022416	D-049086-01	M-049086-00
3	C04	78	Serine/threonine kinase 32C	NM_021302	D-047534-01	M-047534-00
3	C05	77	Serine/threonine kinase 33	XM_358897	D-060060-01	M-060060-00
3	C06	66	Serine/threonine kinase 38	NM_134115	D-062731-01	M-062731-00
3	C07	59	Serine/threonine kinase 38 like	NM_172734	D-040297-01	M-040297-00
3	C08	85	Serine/threonine kinase 39 STE20/SPS1 homolog (yeast)	NM_016866	D-050614-01	M-050614-00
3	C09	66	Serine/threonine kinase 4	NM_021420	D-059385-01	M-059385-00
3	C10	52	Aurora kinase A	NM_011497	D-065109-01	M-065109-00
3	C11	69	Spleen tyrosine kinase	NM_011518	D-041084-01	M-041084-00
3	C12	80	TANK-binding kinase 1	NM_019786	D-063162-01	M-063162-00
3	D03	58	Tec protein tyrosine kinase	NM_013689	D-040988-01	M-040988-00
3	D04	70	Endothelial-specific receptor tyrosine kinase	NM_013690	D-045325-01	M-045325-00
3	D05	59	Testis specific protein kinase 1	NM_011571	D-059125-01	M-059125-00
3	D06	75	Testis-specific kinase 2	NM_146151	D-064149-01	M-064149-00
3	D07	66	Testis expressed gene 14	NM_031386	D-045638-01	M-045638-00
3	D08	86	Transforming growth factor beta receptor I	NM_009370	D-040617-01	M-040617-00
3	D09	71	Transforming growth factor beta receptor II	NM_009371	D-040618-01	M-040618-00
3	D10	69	Tyrosine kinase with immunoglobulin-like and EGF-like domains 1	NM_011587	D-046547-01	M-046547-00
3	D11	73	Tight junction protein 2	NM_011597	D-046662-01	M-046662-00
3	D12	72	Thymidine kinase 1	NM_009387	D-042809-01	M-042809-00
3	E03	66	Thymidine kinase 2 mitochondrial	NM_021028	D-047803-01	M-047803-00
3	E04	79	Tousled-like kinase 1	NM_172664	D-040153-01	M-040153-00
3	E05	88	Tousled-like kinase 2 (Arabidopsis)	NM_011903	D-040154-01	M-040154-00
3	E06	70	Toll-like receptor 1	NM_030682	D-050626-01	M-050626-00
3	E07	74	Toll-like receptor 3	NM_126166	D-059850-01	M-059850-00
3	E08	73	Toll-like receptor 4	NM_021297	D-047487-01	M-047487-00
3	E09	85	Toll-like receptor 6	NM_011604	D-046763-01	M-046763-00
3	E10	76	Tumor necrosis factor receptor superfamily member 10b	NM_020275	D-050949-01	M-050949-00
3	E11	78	TRAF2 and NCK interacting kinase	XM_130797	D-049830-01	M-049830-00
3	E12	85	Tyrosine kinase non-receptor 1	NM_031880	D-063130-01	M-063130-00
3	F03	74	Tyrosine kinase non-receptor 2	NM_016788	D-040775-01	M-040775-00
3	F04	83	Thiamine pyrophosphokinase	NM_013861	D-044055-01	M-044055-00
3	F05	64	Tribbles homolog 1 (Drosophila)	NM_144549	D-057134-01	M-057134-00
3	F06	83	Tribbles homolog 2 (Drosophila)	NM_144551	D-040469-01	M-040469-00
3	F07	62	Tribbles homolog 3 (Drosophila)	NM_144554	D-040943-01	M-040943-00
3	F08	70	Tripartite motif-containing 27	NM_009054	D-046932-01	M-046932-00
3	F09	66	Transformation related protein 53 regulating kinase	NM_023815	D-061023-01	M-061023-00
3	F10	52	Transient receptor potential cation channel subfamily M member 6	NM_153417	D-053986-01	M-053986-00
3	F11	73	Transient receptor potential cation channel subfamily M member 7	NM_021450	D-040716-01	M-040716-00
3	F12	66	Tau tubulin kinase 2	NM_080788	D-047640-01	M-047640-00
3	G03	88	Ttk protein kinase	NM_009445	D-047162-01	M-047162-00
3	G04	81	Titin	NM_011652	D-045920-01	M-045920-00
3	G05	75	TXK tyrosine kinase	NM_013698	D-045811-01	M-045811-00
3	G06	64	Thioredoxin domain containing 3 (spermatozoa)	NM_181591	D-061399-01	M-061399-00
3	G07	61	Tyrosine kinase 2	NM_018793	D-050349-01	M-050349-00
3	G08	89	TYRO3 protein tyrosine kinase 3	NM_019392	D-043798-01	M-043798-00
3	G09	89	UDP-glucose pyrophosphorylase 2	NM_139297	D-061150-01	M-061150-00
3	G10	96	Unc-51 like kinase 1 (C. Elegans)	NM_009469	D-040155-01	M-040155-00
3	G11	77	Unc-51 like kinase 2 (C. Elegans)	NM_013881	D-040619-01	M-040619-00
3	G12	79	Uridine-cytidine kinase 1	NM_011675	D-043765-01	M-043765-00
3	H03	85	Uridine-cytidine kinase 1-like 1	NM_026765	D-056528-01	M-056528-00
3	H04	96	Vaccinia related kinase 1	NM_011705	D-040620-01	M-040620-00
3	H05	79	Vaccinia related kinase 2	NM_027260	D-040621-01	M-040621-00

3	H06	70	Vaccinia related kinase 3	NM_133945	D-040622-01	M-040622-00
3	H07	18	WEE 1 homolog 1 (S. Pombe)	NM_009516	D-040623-01	M-040623-00
3	H08	82	Wnt inhibitory factor 1	NM_011915	D-046832-01	M-046832-00
3	H09	88	Yamaguchi sarcoma viral (v-yes) oncogene homolog 1	NM_009535	D-040156-01	M-040156-00
3	H10	81	Tyrosine 3-monooxygenase/tryptophan 5-monooxygenase activation protein eta polypeptide	NM_011738	D-043652-01	M-043652-00
3	H11	85	Zeta-chain (TCR) associated protein kinase	NM_009539	D-062434-01	M-062434-00

1.2 Image analysis using CellProfiler

Cells were analysed by automated immunofluorescence microscopy using an ArrayScan VTI HCS Reader with 20x magnification. Per well, 81 images were acquired and subjected to quantitative image analysis using CellProfiler. Extracted features included: number, intensity parameters and area shape characteristics of nuclei and AQP2 speckles per nucleus (nuclear outlines expanded by 9 pixels to include perinuclear region). Metadata were further processed in KNIME where an artificial neuronal network was utilized to define hits. The Multi-Layered Perceptron (MLP) consisted of 10 perceptrons and was trained with plate-specific data from mock transfected cells. After training MLP perfomance was tested on NT#2 transfected cells. From in total nine screening plates four could successfully be analysed using this machine learning approach. On the remaining five plates the MLP performance was poor, i.e. NT#2-transfected cells were misclassified and hence, plates were excluded. In addition, hits were defined using the standard Z-score if Z-factor was >0.4. Using this dual approach, hits were described relative to controls (MLP) as well as to other siRNAs on the plate (Z-score).

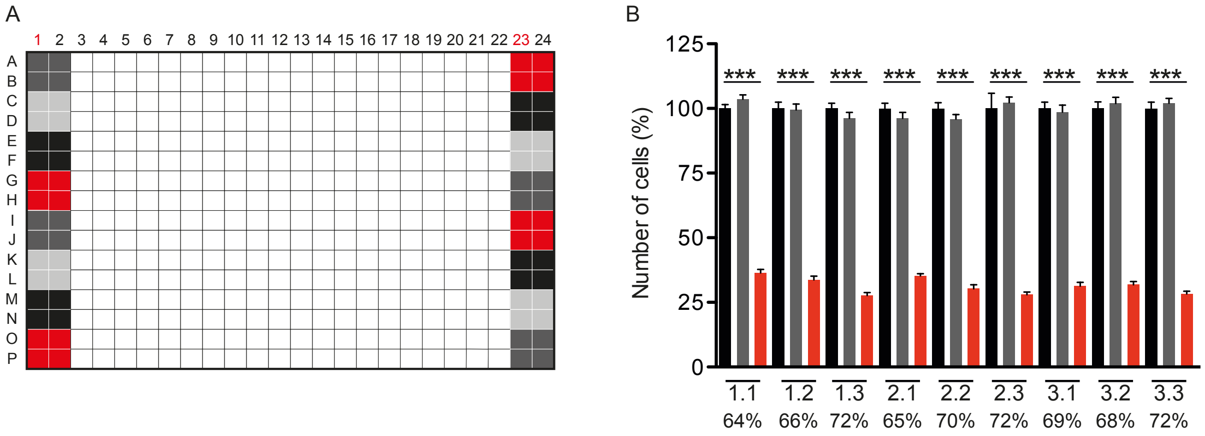

Figure S1. MCD4 cells were transfected with siRNAs of the Mouse Protein Kinases sub-library. A) Library siRNAs were located in the central 320 wells of columns 3-22 of 384-well microtitre plates. Controls were arranged on either side and included cells transfected with AQP2 siRNA, TOX siRNA, NT#2 siRNA and mock-transfected cells. Except for rows 1 and 23, cells were stimulated with 30 μM FSK. **B**) The experiment was performed three times independently, i.e. microtitre plates 1-3 were analysed in triplicate (1.1, 1.2, 1.3, 2.1, 2.2, 2.3, 3.1, 3.2, 3.3). The transfection of TOX siRNA reduced the cell number to 64-72% compared to NT#2 transfected cells. Colour code relates to well definition. Statistically significant differences were determined applying one-way ANOVA with posthoc Bonferroni. ***, p ≤ 0.001. Mean ± SEM are plotted.

1.4 Identification of siRNA affecting cell viability

Several siRNAs included in the Mouse Protein Kinases sub-library strongly reduced cell numbers. Out of 719 tested genes, the knockdown of 24 kinases decreased cell viability to less than 60 % compared to NT#2-transfected cells (Supplemental Table2). To identify targeted genes, siRNA sequences were re-annotated with RefSeq version 77 (www.ncbi.nlm.nih.gov/refseq/announcements/).

	Position	Viability (%)	Protein symbol	Gene name	Accession
1	3/H07	18	WEE1	*WEE 1 homolog 1*	NM_009516.3
2	1/D20	25	COPB2	*Coatomer protein complex subunit beta 2 (beta prime)*	NM_015827.2
3	2/F19	25	PLK1	*Polo-like kinase 1*	NM_011121.4
4	1/I04	33	AURKB	*Aurora kinase B*	NM_011496.2
5	1/A11	43	STK36	*Serine/threonine kinase 36*	NM_175031.3
6	1/J06	50	EVI5L	*Ecotropic viral integration site 5 like*	*
7	2/F20	50	PLK2	*Polo-like kinase 2*	NM_152804.2
8	3/C10	52	AURKA	*Aurora kinase A*	NM_011497.4
9	3/F10	52	TRPM6	*Transient receptor potential cation channel subfamily M member 6*	NM_153417.2
10	2/F09	55	MAP3K2	*Mitogen-activated protein kinase kinase kinase 2*	NM_011946.3
11	1/A22	55	CDKL3	*Cyclin-dependent kinase-like 3*	NM_153785.4
12	1/K06	56	PDIK1L	*PDLIM1 interacting kinase 1 like*	NM_146156.3
13	1/I12	57	THNSL1	*Threonine synthase-like 1*	NM_001001297.2
14	1/F10	57	XYLB	*Xylulokinase homolog*	NM_001033209.3
15	3/D03	58	TEC	*Tec protein tyrosine kinase*	NM_013689.5
16	1/C05	58	MAGI3	*Membrane associated guanylate kinase WW and PDZ domain containing 3*	NM_133853.3
17	2/E10	58	MAP2K4	*Mitogen-activated protein kinase kinase 4*	NM_009157.5
18	1/O04	58	CARD14	*Caspase recruitment domain family member 14*	NM_130886.4
19	1/G10	58	AKAP13	*A kinase (PRKA) anchor protein 13*	NM_029332.1
20	3/C07	59	STK38L	*Serine/threonine kinase 38 like*	NM_172734.3
21	2/I12	59	MARK3	*MAP/microtubule affinity-regulating kinase 3*	NM_021516.4
22	3/D05	59	TESK1	*Testis specific protein kinase 1*	NM_011571.3
23	1/H11	59	ARAF	*V-raf murine sarcoma 3611 viral oncogene homolog*	NM_009703.2
24	1/M10	59	C230081A13RIK	*RIKEN cDNA C230081A13 gene*	NM_172924.3

Table S2. MCD4 cell viability is decreased upon down regulation of the listed genes. Cells were transfected with a pool of four siRNAs targeting transcripts of the indicated genes. As a control, toxicity was induced with Tox siRNA. Three days after transfection, cells were analysed by automated immunofluorescence microscopy. During image analysis, numbers of nuclei were determined within the CellProfiler pipeline. NT#2-transfected cells were considered as 100 % viable. Mean viability calculated from three independent experiments is shown. Position, indicates the microtitre plate and well of detected candidates. Accession numbers refer to National Center for Biotechnology Information (NCBI, www.ncbi.nlm.nih.gov/) entries. *, NM_153536.2 permanently suppressed on NCBI because it is a nonsense-mediated mRNA decay (NMD) candidate.

1.7 Hits

	Microtitre plate/well	Viability (%)	Protein symbol	Gene name	Accession
**1**	**2.1/E22**	**97**	**PKIA**	***cAMP-dependent protein kinase inhibitor***	**NM_008862.3**
2	3.3/A09	87	STK11	*Serine/threonine kinase 11*	NM_011492.4
3	1.3/L20	79	FGFR1	*Fibroblast growth factor receptor 1*	NM_010206.3
4	1.3/K17	78	EPHB3	*Eph receptor B3*	NM_010143.1
5	1.3/L08	75	BMPR1B	*Bone morphogenetic protein receptor type 1B*	NM_007560.4
6	3.3/H06	70	VRK3	*Vaccinia related kinase 3*	NM_133945.1
7	3.3/F08	70	TRIM27	*Tripartite motif-containing 27*	NM_009054.3
**8**	**2.1/A21**	**70**	**CDK18**	***Cyclin-dependent kinase 18***	**NM_008795.2**
9	1.3/G06	69	AK3	*Adenylate kinase 3*	NM_021299.2
10	3.3/C11	69	SYK	*Spleen tyrosine kinase*	NM_011518.2
11	3.3/E03	66	TK2	*Thymidine kinase 2, mitochondrial*	NM_021028.3
12	1.3/I14	65	DUSP2	*Dual specificity phosphatase 2*	NM_010090.2
13	2.1/C13	64	PFKP	*Phosphofructokinase 1*	NM_019703.4
14	3.3/F10	52	TRPM6	*Transient receptor potential cation channel subfamily M member 6*	NM_153417.2

15	3.3/C10	52	AURKA	*Aurora kinase A*	NM_011497.4
16	1.3/J06	50	EVI5l	*Ecotropic viral integration site 5 like*	NM_001039578.4
17	1.3/I04	33	AURKB	*Aurora kinase B*	NM_011496.2
18	1.3/D20	25	COPB2	*Coatomer protein complex subunit beta 2 (beta prime)*	NM_015827.2
19	3.3/H07	18	WEE1	*WEE 1 homolog 1*	NM_009516.3

Table S3. Hit list. Upon the knock down of the expression of the indicated genes, the AQP2 redistribution in MCD4 cells was inhibited. Hits are listed according to MCD4 cell viability caused by siRNA-mediated silencing of gene expression. Viability of NT#2-transfected cells was considered 100 %. Two genes that were detected twice are highlighted. The down regulation of candidates 14-19 decreased MCD4 cell viability to less than 60 %. Accession numbers refer to National Center for Biotechnology Information (NCBI, www.ncbi.nlm.nih.gov/) entries, Release 77.

2. Supplementary Table S4, Figures S2-S4

Table S4. Proteins interacting with CDK18

Gene name	adj.P.Val.S12. over.K150	logFC.S12.over. K150	P.Value.S12.over .K150	LFQ.K150_1	LFQ.K150_2	LFQ.K150_3	LFQ.S12_1	LFQ.S12_2	LFQ.S12_3	Uniprot
Ccna2	2,23787E-05	-3,995449907	3,16084E-08	33,15240031	33,24487678	33,19932088	29,10026576	29,12728526	29,38269724	P51943
Fkbp5	3,4777E-05	1,529009648	9,82402E-08	31,69789042	31,70450166	31,75736215	33,24303308	33,25067615	33,25307394	Q64378
Atp1a1	3,99439E-05	-3,534435665	1,69254E-07	26,35984498	26,27148981	26,10465602	22,78624395	22,81462381	22,53181607	Q8VDN2
Cdk1	6,46719E-05	-2,482925954	4,00559E-07	32,80793203	32,75958601	32,70340877	30,4245233	30,27697382	30,12065183	P11440
Ppm1b	6,46719E-05	-1,199376028	5,10198E-07	35,69677696	35,63895467	35,61522117	34,45316901	34,43402747	34,46562823	Q99NF7
Dnaja2	6,46719E-05	1,160108246	5,48067E-07	33,2018639	33,15191666	33,12809386	34,31338187	34,33969925	34,30911804	Q9QYJ0
Cks2	0,000132075	-5,638164639	1,50118E-06	29,74167423	29,84814971	29,83826379	23,96985205	24,71259135	23,8311504	P56390
Myo1c	0,000132075	-2,679400397	2,61165E-06	29,03328167	29,02960065	29,00469256	26,57007689	26,05831434	26,40098247	Q9WTI7-2
Gcn1l1	0,000132075	-1,058177748	2,10945E-06	30,93901639	30,86548048	30,94363436	29,91570216	29,84098167	29,81691414	E9PVA8
Rpl23	0,000132075	0,97379689	2,19718E-06	31,52477292	31,55882884	31,61056476	32,50873773	32,57668657	32,5301329	P62830
Dnaja1	0,000132075	1,000279969	1,94607E-06	32,50979972	32,42274524	32,41588799	33,42629917	33,46063303	33,46234065	P63037
Tma7	0,000132075	1,378273172	2,37803E-06	29,10946145	29,01709414	28,88939613	30,35405388	30,39897033	30,39774702	Q8K003
Stip1	0,000132075	1,381786741	1,71447E-06	29,37123326	29,48563934	29,49937257	30,76817127	30,81573909	30,91769503	Q60864
Dctn3	0,000132075	4,952420479	2,51707E-06	24,53294494	24,39771492	24,42942387	29,92365718	29,24499584	29,04869216	Q9Z0Y1
Hsp90ab1	0,000146847	0,771944953	3,94081E-06	35,66857819	35,63636382	35,65122477	36,45607968	36,4038945	36,41202746	P11499
Ruvbl1	0,000146847	0,872034604	3,5724E-06	32,85855016	32,95821304	32,91117239	33,76435971	33,7916856	33,7879941	P60122
Hspd1	0,000146847	0,932196822	3,33194E-06	31,07214275	31,18565235	31,11966283	32,07676836	32,06268599	32,03459405	P63038
Ruvbl2	0,000146847	0,971237923	3,75613E-06	32,89258409	32,97281703	32,86538829	33,83158626	33,8993352	33,91358172	Q9WTM5
Ppp2r2a	0,000146847	1,072827477	3,35108E-06	30,16446912	30,08967329	30,09017826	31,17640807	31,12256608	31,26382895	Q6P1F6
Cdc37	0,000155727	0,915368549	4,39906E-06	33,69664743	33,71466912	33,69809748	34,54512785	34,67103276	34,63935906	Q61081
Bloc1s2	0,000169634	1,278591959	5,0315E-06	27,94949048	27,72832205	27,80955815	29,14401632	29,13374138	29,04538886	Q9CWG9
Shmt1	0,000170343	-5,307919674	6,97732E-06	28,72669911	28,79274855	28,8706188	23,49011996	22,89052147	24,08566599	G3UZ26

Cks1b	0,000170343	-4,906737674	6,08965E-06	28,76992161	28,59583806	28,77577217	23,21367687	24,2389654	23,96867654	P61025
Scyl2	0,000170343	-0,701635102	6,80124E-06	30,6551442	30,64787361	30,6842001	29,93817516	29,99158892	29,95254851	G5E8J9
Tubb4b	0,000170343	0,77969819	5,68994E-06	35,04653091	35,00110225	35,01625241	35,84887071	35,77369789	35,78041154	P68372
Rps3a	0,000170343	0,847230881	5,4511E-06	32,74737212	32,72417587	32,82767388	33,60917811	33,5901148	33,64162161	P97351
Rps13	0,000170343	0,870302617	6,12708E-06	30,10736759	30,00544235	30,10985994	30,91541724	30,98005544	30,93810504	P62301
Rpl7a	0,000170343	0,880607287	6,78148E-06	27,88867061	27,90305464	27,92846569	28,81222349	28,84149165	28,70829768	P12970
Capn2	0,000170343	1,13925729	6,3519E-06	29,02575185	29,1597379	28,94720663	30,1472229	30,20629614	30,1969492	O08529
Rps27	0,000178748	1,120735945	7,60462E-06	30,25979469	30,252933	30,17355172	31,4270239	31,390744	31,23071934	A0A0G2JDW7
Rcn2	0,000178748	2,051965986	7,82654E-06	27,29745354	26,8460135	27,16144485	29,22301558	29,07227055	29,16552372	Q8BP92
Cdk2	0,000213273	-3,285070028	9,64811E-06	30,59965577	30,52952799	30,5938809	27,70530122	27,27399887	26,88855449	P97377
Ttc1	0,000213273	1,706565805	9,94068E-06	27,43157386	27,77950286	27,40681562	29,24850624	29,24333959	29,24574393	Q91Z38
Tecr	0,000219991	-1,471071445	1,17665E-05	26,56098348	26,55971515	26,72663415	25,1217093	25,00298803	25,30942112	Q9CY27
Ubr1	0,000219991	-1,381583317	1,20882E-05	31,46810721	31,41613975	31,36671791	30,16290977	30,09017826	29,85312689	O70481
Bag2	0,000219991	0,816618101	1,15658E-05	28,5164935	28,50479917	28,51747002	29,41701552	29,29482727	29,2767742	Q91YN9
Ppp2r1a	0,000219991	0,877170675	1,12478E-05	32,09304691	32,02115146	31,93075823	32,86796727	32,88139528	32,92710609	Q76MZ3
Tubb6	0,000219991	0,959144452	1,21182E-05	30,11607204	30,05646532	30,19048787	31,16194933	31,00470595	31,07380329	Q922F4
Hist1h2ah	0,000219991	2,989795476	1,09712E-05	24,28369302	23,8193735	24,23247762	26,82602258	27,40543664	27,07347134	A0A0N4SV66
Rps14	0,000225361	0,925895571	1,27323E-05	31,8852194	31,71038493	31,83872213	32,73912475	32,77721543	32,69567301	P62264
Rps12	0,000225849	1,028811685	1,30788E-05	31,00202499	30,78175541	30,89792982	31,96133891	31,92705309	31,87975326	Q6ZWZ6
Rps16	0,000228057	1,00898121	1,35288E-05	31,6625481	31,54070113	31,5498164	32,69397137	32,5098469	32,57619099	P14131
Ap2b1	0,000231429	-1,798881824	1,45838E-05	25,67236124	26,07142685	25,96574026	24,21770843	23,97668213	24,11849231	Q9DBG3
Hsp90aa1	0,000231429	0,711379047	1,45764E-05	34,01496479	34,09057264	33,97168702	34,74745216	34,72212071	34,74178873	P07901
Ppp2r5c	0,000231429	0,891393777	1,47095E-05	28,3726453	28,23653586	28,26132007	29,14537756	29,142386	29,256919	Q60996-3
Rps8	0,000242221	0,775188728	1,57375E-05	30,30421601	30,18930997	30,20885597	30,96444082	31,00504072	31,05846659	P62242
Bzw1	0,00025853	-1,647011335	1,7228E-05	27,89006329	27,88547398	27,96944493	26,0185858	26,43386734	26,35149505	Q9CQC6
Cox6b1	0,00025853	-0,977224533	1,75274E-05	26,48905525	26,57321575	26,71384607	25,58835093	25,6510806	25,60501193	P56391
Rps2	0,000267866	0,714204481	1,85387E-05	31,03360988	31,07227055	30,93964698	31,71579443	31,74165815	31,73068826	P25444
Ubr2	0,000285018	-0,628256755	2,09335E-05	30,97295394	31,06136674	31,05736946	30,39386633	30,42031252	30,39274103	Q6WKZ8-2
Rps28	0,000285018	0,614455878	2,06428E-05	31,18352431	31,11303145	31,19489646	31,79683788	31,75998277	31,7779992	G3UYV7

Rpl3	0,000285018	0,652485786	2,04831E-05	28,69208894	28,65836573	28,6284318	29,26816439	29,30419425	29,36398519	P27659
Fv4	0,000303077	-1,661926407	2,2688E-05	25,43316694	25,19321221	25,47833958	23,52388424	23,70034882	23,89470645	P10404
Rps24	0,000348922	0,569608954	2,66127E-05	29,71726623	29,77033129	29,76281247	30,2831704	30,33383455	30,34223191	P62849-2
Ciao1	0,000355615	0,678886142	2,76255E-05	27,32156551	27,31388998	27,42478357	28,06496769	28,0429651	27,98896469	Q99KN2
Rpl35	0,000389383	0,62580781	3,24013E-05	29,55426104	29,50693763	29,59334667	30,14916282	30,14928398	30,23352196	Q6ZWV7
Rps11	0,000389383	0,652069659	3,30695E-05	31,94167702	31,90034334	31,86271235	32,60935427	32,56226798	32,48931944	A0A1B0GRR3
Rpl4	0,000389383	0,702442336	3,25586E-05	28,52813107	28,54697751	28,58652675	29,263023	29,17524241	29,33069693	Q9D8E6
Rpl24	0,000389383	0,705274047	3,35485E-05	29,76097419	29,61494922	29,65938694	30,38288273	30,34392671	30,42432307	Q8BP67
Usp9x	0,000389383	0,707690718	3,27968E-05	29,81831684	29,89379945	29,80961948	30,55508431	30,4740201	30,61570352	Q4FE56
Tuba4a	0,000389383	0,796848072	3,31539E-05	30,16362968	30,31646022	30,15653493	31,06233216	30,95905994	31,00577695	P68368
Rps26	0,000406861	0,981525796	3,5629E-05	28,66444826	28,67168661	28,89729515	29,72343147	29,78017586	29,67440008	P62855
Atl3	0,000419208	-1,300909269	3,96708E-05	25,04918051	25,10809082	25,17538995	24,02171579	23,77247683	23,63574084	E9PYT3
Atp5a1	0,000419208	-0,892193461	3,89875E-05	27,72090658	27,83478765	27,91382065	27,01998582	26,85269542	26,92025326	Q03265
Htra1	0,000419208	-0,595938221	3,76203E-05	29,02857355	28,96162871	28,99239958	28,4568153	28,35779138	28,38018049	Q9R118
Atp5b	0,000419208	-0,582618604	3,86855E-05	27,61946909	27,63270172	27,57080073	26,96829168	27,05185829	27,05496577	P56480
Rps9	0,000419208	0,718060591	3,95316E-05	29,72483047	29,65165889	29,54809903	30,39028276	30,34297363	30,34551377	Q6ZWN5
Rps4x	0,000428034	0,560488462	4,14442E-05	31,84029893	31,83439587	31,92386966	32,42759843	32,40400557	32,44842584	P62702
Rps18	0,000428034	0,762382304	4,17152E-05	32,32262416	32,20089983	32,33931432	32,96872082	33,10274537	33,07851903	P62270
Rab1	0,00045586	-0,758983658	4,50709E-05	29,04980888	29,18560509	29,12341741	28,27604203	28,43714197	28,36869641	Q5SW88
S100a11	0,000467445	-0,553032751	4,74599E-05	28,38199629	28,43061717	28,31803466	27,8298374	27,81775288	27,8239596	P50543
Rpl38	0,000467445	0,588374628	4,77242E-05	33,60476721	33,56001411	33,49702771	34,13093419	34,10984438	34,18615435	Q9JJI8
Mltk	0,000467445	0,998057234	4,81971E-05	25,9309598	26,01614313	26,01616439	26,8148228	27,05734364	27,08527259	Q9ESL4-2
Vat1	0,000476625	-0,654787326	5,11631E-05	27,85061064	27,71620341	27,85037219	27,10858938	27,16873072	27,17550417	Q62465
Hspa5	0,000476625	-0,619024165	5,10316E-05	31,66928068	31,62270139	31,63079397	31,05872462	31,06894401	30,93803491	P20029
Ywhae	0,000476625	0,45990854	5,04458E-05	30,72557821	30,75286423	30,74711198	31,19759375	31,18748232	31,22020398	P62259
Canx	0,000502903	-1,235323186	5,46942E-05	27,02030382	27,10429609	26,83725642	25,67957352	25,64463197	25,93168128	P35564
Tuba1b	0,000505815	0,550857379	5,57254E-05	35,3163186	35,37803805	35,28612789	35,88178271	35,83655592	35,91471806	P05213
Cdk15	0,000528674	-0,657109831	5,89904E-05	34,85340951	35,0046641	34,84338184	34,25166395	34,23763026	34,24083174	Q3V3A1-3
Gnai2	0,000543224	-2,113535262	6,21485E-05	26,61876926	26,46766811	26,6759988	24,73612681	24,62624603	24,05945754	P08752

Rps20	0,000543224	0,849816905	6,15904E-05	29,50144316	29,26291102	29,33853884	30,17247913	30,28350161	30,196363	P60867
Ppp2r5b	0,000563859	1,105884209	6,53057E-05	26,32089445	26,07443125	25,99323678	27,14812044	27,2429082	27,31518647	Q6PD28
Cct8	0,000578981	0,436503446	6,88849E-05	34,25032319	34,22618932	34,24104492	34,69830451	34,65741408	34,67134918	P42932
Eif4a1	0,000578981	0,58861957	6,95104E-05	29,44365715	29,3014058	29,3783624	29,99859951	29,93494593	29,95573862	P60843
Mapk6	0,000578981	0,784241208	6,84091E-05	27,62268393	27,56034216	27,74586263	28,37832105	28,38240865	28,52088264	Q61532
Ppp2cb	0,000596927	0,727287882	7,25081E-05	30,39907223	30,48682873	30,61990631	31,25163574	31,2301467	31,20588848	P62715
Myh9	0,000662049	-0,553834969	8,17021E-05	31,30437923	31,29455343	31,25889665	30,73682878	30,7991562	30,66033942	Q8VDD5
Rpl10	0,000662049	0,599345352	8,22886E-05	28,93606996	28,85141513	28,96628501	29,44754431	29,56723228	29,53702957	Q6ZWV3
Rplp0	0,000705015	0,52329046	8,86248E-05	28,60052445	28,59284787	28,50555651	29,11388843	29,11306872	29,04184306	P14869
Rpl7	0,000705307	0,678915642	8,96577E-05	26,97136498	26,98761012	26,91501826	27,74239723	27,53897287	27,62937018	P14148
Rpl27a	0,000731297	0,554678398	9,39944E-05	28,53155578	28,51765774	28,41856444	29,09906198	29,01438291	29,01836825	P14115
Alb	0,000738939	-0,432644273	9,60203E-05	30,08080761	30,01278568	30,06342553	29,63152268	29,61465091	29,61291241	P07724
Arl1	0,0007771	-1,430002845	0,000102077	26,29519524	26,41739788	26,47946562	24,6646517	25,10521573	25,13218277	P61211
Rpl37a	0,000796434	0,616295977	0,000105741	27,89937121	27,92263684	28,01786405	28,64924499	28,53029107	28,50922398	P61514
Dcaf8	0,000972487	0,52138	0,000130489	29,80780923	29,72671535	29,72897071	30,32087724	30,20384843	30,30290962	Q8N7N5
Rps10	0,001032595	0,729095728	0,000140013	31,19706641	31,09421166	31,13258974	31,73517503	31,89269264	31,98328733	P63325
Rps5	0,0010762	0,444943286	0,000148951	31,72744589	31,79436088	31,74358539	32,14907195	32,21699969	32,23415038	Q91V55
Rps3	0,0010762	0,613899287	0,000148966	31,81676922	31,61289484	31,72317918	32,33220789	32,36736903	32,29496417	P62908
Hmgb2	0,001157176	-0,462194154	0,000161809	28,86855978	28,88439716	28,94187288	28,45481686	28,37608656	28,47734393	P30681
Tpd52	0,001159328	-0,680951801	0,000163747	28,69584306	28,88901891	28,71538533	28,05382714	28,03406924	28,16949552	Q62393-2
Rpl17	0,001202924	0,472272929	0,000171604	30,0188988	30,04856225	29,97185829	30,41780025	30,5294349	30,50890298	Q9CPR4
Wdr77	0,001215147	-0,380437796	0,000175064	35,79592401	35,84333504	35,85498891	35,44699122	35,43988951	35,46605384	Q99J09
Pdia3	0,001252198	-1,033760116	0,000183939	26,69165642	26,35909094	26,4746468	25,55602044	25,31011487	25,5579785	P27773
Rpl13	0,001252198	0,861528212	0,000182606	28,81311084	28,58742125	28,66857925	29,46072579	29,48371889	29,70925129	P47963
Rsrc2	0,001334302	0,374163193	0,000197884	26,27675202	26,24192687	26,29913184	26,65948902	26,62268393	26,65812735	S4R2L4
Lima1	0,001353228	-1,06447481	0,000203411	26,39751039	26,74586263	26,32988473	25,41456594	25,36536263	25,49990475	Q9ERG0-2
Rps23	0,001353228	0,628637946	0,000204513	30,23443593	30,16674513	30,27175249	30,72947323	30,88692787	30,94244629	P62267
Try10	0,001361025	-0,361205381	0,000207614	29,00613823	29,02485407	28,98098152	28,66067944	28,62090268	28,64677556	Q792Z1
Twf1	0,001398065	0,684895626	0,000215239	26,97475994	26,91250784	26,92829626	27,62331209	27,48761473	27,75932409	Q91YR1

H2afv	0,001413002	0,763441583	0,00022153	28,47072738	28,4126818	28,26789659	29,21148089	29,00520139	29,22494825	Q3THW5
Wdr92	0,001413002	0,996370354	0,000220949	25,26366779	25,20947895	25,39969568	26,06585113	26,44600648	26,35009587	Q8BGF3
Cct4	0,001439543	0,381500396	0,000227724	33,78653432	33,78234143	33,747152	34,18408618	34,16690971	34,10953306	P80315
Rpl12	0,001491608	0,670166784	0,000238067	26,77064635	26,88901891	26,91524626	27,59733084	27,39452066	27,59356038	P35979
Por	0,001544569	-1,699494769	0,00024946	27,47258772	26,89017928	27,00656629	25,71332169	25,30872703	25,24880027	P37040
Nop16	0,001544569	0,636705438	0,000250883	28,10194434	28,09032971	28,28991208	28,77470448	28,8754899	28,74210807	Q9CPT5
Rps7	0,0015681	0,384491624	0,00025692	30,40182071	30,38030437	30,42101517	30,81665479	30,80708756	30,73287276	P62082
Gnb1	0,001568522	-0,62508518	0,000261702	26,3334891	26,17268417	26,31962031	25,56874406	25,6276737	25,75412028	H3BKR2
Ighg1	0,001568522	-0,516592702	0,000268876	32,96607525	33,14363932	33,04059932	32,50831271	32,58277726	32,50944581	P01868
Polr2e	0,001568522	0,607651497	0,000259854	26,56096891	26,55961305	26,62784065	27,31708589	27,0820267	27,17226451	Q80UW8
Apol9a	0,001568522	0,640849454	0,000274713	26,88087004	27,06896963	26,97235144	27,54554216	27,72767309	27,57152421	Q8VDU3
Rpl13a	0,001568522	0,783558232	0,000267815	26,61133014	26,747144	26,77555241	27,47142529	27,65594599	27,35732997	A0A1B0GSQ<br>6
Hist1h4a	0,001568522	0,951829644	0,000271243	27,36944545	27,51773282	27,73595777	28,55603506	28,35602883	28,56656108	P62806
Rps21	0,001568522	1,070474259	0,000274033	27,48531304	27,56041506	27,30338877	28,64457696	28,64134168	28,274621	Q9CQR2
Hist1h3b	0,001568522	1,08983221	0,000264974	27,57931479	27,97667293	28,03417422	29,01472874	28,93179397	28,91313585	P84228
Rpl5	0,001662099	0,822257482	0,00029345	27,76211547	27,75072579	27,4300588	28,47475509	28,46287204	28,47204537	P47962
A2m	0,001748301	-1,227659729	0,000311138	25,60243722	25,43010667	25,17653172	24,41496518	24,04813116	24,06300009	Q61838
Cct5	0,00175163	0,390089142	0,000319689	34,00946912	33,95280884	34,00921882	34,43564268	34,36821505	34,33790646	P80316
Rpl23a	0,00175163	0,477126775	0,000316979	30,50937502	30,50388984	30,53473125	31,09572128	30,92874805	30,9549071	P62751
Rps25	0,00175163	0,483225004	0,000321627	32,07915511	31,90540941	31,95178461	32,4589967	32,43718166	32,48984577	A0A1L1SQA<br>8
Kpna3	0,00175163	0,490935416	0,000321181	27,13965658	27,24517723	27,1669127	27,62142681	27,63733917	27,76578677	O35344
Tubg1	0,00176357	0,505458362	0,00032631	26,76572355	26,62672725	26,62742323	27,1493809	27,1365309	27,25033731	P83887
Kpna4	0,001825968	-0,523943168	0,000340613	26,51379336	26,63505692	26,64223727	26,16674035	26,03170524	26,02081246	O35343
Psmb7	0,001825968	-0,514405333	0,000343014	29,46690227	29,42151685	29,32643851	28,89951528	28,8084845	28,96364185	P70195
Ppp2r1b	0,001833914	0,568859785	0,000347097	28,10334584	27,90626995	27,95199298	28,57365637	28,4815842	28,61294755	Q7TNP2
Drg1	0,001841731	-0,456524465	0,000351177	30,15158406	30,15930483	30,19671475	29,75070982	29,77105583	29,6162646	P32233
Rpl10a	0,001844977	0,599522196	0,000354402	27,71711912	27,84451777	27,73569959	28,3619793	28,25295555	28,48096821	Q5XJF6
Cct7	0,001897024	0,342307474	0,000367079	33,70388314	33,67282487	33,75433854	34,05948231	34,05431569	34,04417097	P80313
Mars	0,001912531	-0,423321015	0,000372781	27,39009822	27,42045307	27,28056223	26,96466114	26,94044534	26,91604399	Q68FL6

Anxa2	0,001938058	-0,679781923	0,000380855	29,24322607	29,37542383	29,2069016	28,67114668	28,67276587	28,44229318	P07356
Ppp6r3	0,001938058	2,235834042	0,000383232	25,83557089	24,81733814	25,62775718	27,52984444	27,79693068	27,66139321	Q922D4-2
Psmb3	0,002005628	-0,544177394	0,000399426	29,27721776	29,29821863	29,14508597	28,78640579	28,68905859	28,6125258	Q9R1P1
Tcp1	0,002110104	0,310623485	0,000423213	34,17124168	34,18297701	34,15888367	34,50923342	34,47230204	34,46343734	P11983
Tubb2b	0,002115324	0,558837905	0,000427248	28,99967505	29,08458852	29,04210408	29,48531304	29,59270533	29,72486299	Q9CWF2
Ppp2r5e	0,002124796	1,022822281	0,000432162	26,31725844	26,10535562	26,56475272	27,48891739	27,32928598	27,23763026	Q3UZJ4
Cct2	0,002194018	0,297615128	0,00044934	34,1678818	34,13407506	34,16151393	34,45181892	34,45237138	34,45212587	P80314
Rps19	0,002197501	0,649967981	0,000453157	31,81608255	32,01051991	31,92514387	32,6342391	32,42449828	32,6429129	Q9CZX8
Nudt5	0,002278638	0,767464065	0,000473107	25,74473408	25,98754507	25,79968735	26,45464432	26,74534976	26,63436461	Q9JKX6
Ncl	0,002285479	0,338724614	0,000477756	31,74181886	31,70326436	31,72927062	32,10696218	32,06698929	32,01657621	P09405
Cdk4	0,00229552	-0,646173129	0,000483097	25,84840345	25,85884876	25,83942064	25,32144509	25,26399008	25,0227183	P30285
Cse1l	0,002409675	-0,631994638	0,000510524	29,28029672	29,28475951	29,02141625	28,51810816	28,60194156	28,57043886	Q9ERK4
Basp1	0,002440017	-0,996575225	0,000524269	28,74820027	29,14913859	28,65164178	27,87249876	27,87566565	27,81109056	Q91XV3
Eif2b1	0,002440017	-0,388306875	0,000527292	28,80522799	28,89489819	28,81659377	28,49830763	28,38808727	28,46540442	Q99LC8
Cct3	0,002440017	0,339445169	0,000525056	34,10016235	34,08496226	34,08123631	34,38258645	34,41727211	34,48483786	P80318
Prmt5	0,002476146	-0,320394316	0,000538597	37,01296879	36,96634604	37,03721113	36,69721726	36,70148388	36,65664187	A0A0R4J049
Ddb1	0,002480384	0,288483688	0,000543022	31,3075854	31,26930196	31,29603157	31,58481672	31,57655142	31,57700184	Q3U1J4
Slc25a3	0,002520491	-0,785406799	0,000555362	26,85614357	26,60399391	26,82735589	25,88632323	26,15516488	25,88978486	Q8VEM8
Rpl34	0,00268251	0,769644067	0,00059485	27,16115649	26,91809328	27,16557164	27,67969432	27,94519831	27,92886098	Q9D1R9
Thrap3	0,002757536	-0,354512931	0,000615382	31,31397645	31,28719496	31,37331969	31,02221032	30,96147692	30,92726507	Q569Z6
Rpl19	0,002787043	0,707525111	0,000625904	27,86135957	27,86153705	27,95704038	28,5734758	28,79324484	28,43579169	A2A547
Ywhaz	0,002791095	0,321065161	0,000630756	31,44302475	31,48654107	31,45419914	31,73299402	31,79536767	31,81859874	P63101
Pmvk	0,002870117	0,697805104	0,000652668	25,51647096	25,35682644	25,49411629	26,10377603	26,01801268	26,33904029	D3YXP6
Ahnak	0,002920027	-0,612522449	0,000672266	28,43463898	28,30190725	28,41623037	27,88296986	27,60858343	27,82365597	E9Q616
Uchl5	0,002920027	-0,393851608	0,000671954	30,13589517	30,2405901	30,28526678	29,86739661	29,80068426	29,81211636	Q9WUP7-2
Ypel5	0,002967458	-0,314578925	0,000687377	28,95969565	29,04257379	28,98060027	28,68802482	28,69806435	28,65304377	P62700
Cct6a	0,00303903	0,280881636	0,000708249	33,94270845	33,930159	33,93850821	34,20069525	34,24898118	34,20434413	P80317
Rplp1	0,003160851	0,8192301	0,000741103	25,5798902	25,74208879	25,39473873	26,41105784	26,53394824	26,22940194	P47955
Rdx	0,003313797	-0,876047109	0,000781644	25,44426953	25,61742464	25,39910891	24,82565874	24,62950915	24,37749387	Q7TSG6

Prpf38a	0,003315037	0,939777928	0,000786619	24,86702832	24,81151934			25,70493199	25,85317152	Q4FK66-2
U2af2	0,003344771	0,330485879	0,000803123	28,47099883	28,38619688	28,49739419	28,77200033	28,80857656	28,76547065	Q80XR5
Tuba1a	0,003344771	0,813891524	0,000801801	30,34021675	30,27353209	30,56819319	31,41089413	31,1112289	31,10149357	P68369
Ppp2ca	0,003475919	1,098559957	0,000839523	26,97814694	27,12326939	27,0204098	27,8979875	28,01987981	28,49963869	P63330
Rpl8	0,003761856	0,84196307	0,000913897	26,93111766	26,95593258	27,32044691	28,0326513	27,91404884	27,78668621	P62918
Ate1	0,00390611	-0,507999668	0,000954459	29,6412383	29,54885236	29,70581205	29,0240086	29,09893653	29,24895857	Q4FCQ7
Ppib	0,003994905	-0,363284979	0,000981799	28,56616184	28,67165287	28,58305085	28,30843194	28,18508518	28,2374935	P24369
Psmd8	0,004016484	-0,391606108	0,000995914	29,7926865	29,68470173	29,77575647	29,4402151	29,35070712	29,28740415	Q9CPS5
Hspb1	0,004016484	0,37888732	0,001004121	31,83035888	31,92262976	31,94987309	32,24830264	32,23509249	32,35612855	P14602
Eny2	0,004016484	0,80310128	0,001002891	25,94156509	26,33306251	26,23642637	27,12129431	26,92897389	26,87008962	A0A2I3BRK6
Ddhd2	0,004080367	0,764907926	0,001025855	30,31980117	29,97514274	30,30105686	30,95220133	30,84393401	31,09458921	Q80Y98
Rps27l	0,004242919	0,714836418	0,001072715	31,09068305	31,12804775	31,46387579	31,8652961	31,97148147	31,99033827	Q6ZWY3
Sf3b3	0,004307657	0,286357537	0,001095167	28,99746934	28,95324261	29,00477291	29,26154425	29,31615808	29,23685514	Q921M3-2
Rpl36a	0,004317703	0,453501164	0,001109918	29,88227026	29,74024321	29,91698361	30,20583023	30,34074733	30,35342301	P83882
Ptges3	0,004317703	0,867468588	0,001105834	26,1976617	25,67554124	26,04618546	26,84349978	26,81433388	26,86396051	Q9R0Q7
Rps15	0,004365964	0,333426752	0,001134657	29,383851	29,39931675	29,36133097	29,79660584	29,69060786	29,65756528	P62843
Ascc1	0,004365964	1,020334041	0,001129683	24,1017893	24,67550611	24,17299428	25,40973146	25,3481719	25,25338846	Q9D8Z1
Asf1a	0,004593832	3,983329118	0,001200366	24,48096913	22,658943	25,06405186	28,15590796	28,02991654	27,96812685	Q9CQE6
Gvin1	0,00470685	-0,33538014	0,001236545	30,84759729	30,88968624	30,90863379	30,51831455	30,49266554	30,6287968	L7N451
Rab6a	0,004861976	-0,86270056	0,001284166	29,29749727	28,93668781	29,17859412	28,12504465	28,18791881	28,51171405	P35279-2
Rpl6	0,005002324	0,561196914	0,001328301	27,90615524	27,74284692	27,87689535	28,54399479	28,41651227	28,24898118	P47911
Ehd2	0,005080331	-0,434765123	0,001387578	27,48008226	27,45677614	27,27657455	27,01062661	26,99356072	26,90495024	Q8BH64
Psmb2	0,005080331	-0,356801565	0,001388948	28,1350634	28,0517546	28,06316834	27,68257696	27,81945934	27,67754535	Q9R1P3
Psmb5	0,005080331	-0,334443821	0,00138693	29,9648539	29,94307539	29,92719441	29,69872676	29,59004785	29,54301763	O55234
Bclaf1	0,005080331	-0,301783874	0,00141168	29,86584916	29,83793309	29,85086395	29,50820407	29,51641836	29,62467215	Q8K019-2
Psmd3	0,005080331	-0,300117147	0,001413595	31,23500687	31,12040464	31,18316933	30,89366928	30,90167446	30,84288565	P14685
Rps15a	0,005080331	0,382953522	0,001406717	28,89891012	28,83517932	28,77052034	29,1336679	29,29743167	29,22237078	P62245
Ywhag	0,005080331	0,418626062	0,001408551	29,80667283	29,69738506	29,62672725	30,05012037	30,1323446	30,20419835	P61982
Tubb5	0,005080331	0,672150965	0,001361671	33,15344263	33,16520322	33,28540459	34,08694024	33,84408371	33,68947939	P99024

Ascc3	0,005080331	1,25596358	0,001396379	25,16714247	24,57961063	25,35930882	26,22324579	26,37985836	26,27084851	E9PZJ8
Ldha	0,005123392	-0,283658858	0,001432813	29,95712344	29,96182187	29,88473194	29,61785898	29,69110727	29,64373442	Q564E2
Dctn5	0,005302994	0,372007862	0,001490531	24,83691947	24,77525098	24,78196797	25,19169952	25,06554301	25,25291947	Q9QZB9
Myl12a	0,005456215	0,290050902	0,001541304	29,52017078	29,58686673	29,50699436	29,78183356	29,82791611	29,87443491	Q6ZWQ9
Copb1	0,005536259	-0,476653795	0,001571734	27,80593522	27,63006489	27,88279499	27,34358791	27,21835733	27,32688848	Q9JIF7
Pfdn5	0,005567068	0,584808684	0,001588344	24,51859051	24,65627688	24,82138201	25,12541915	25,28355018	25,34170612	H7BWX1
Q9DAM7	0,005605384	-1,971446957	0,001623028	25,56185754	25,62385627	25,52686335	24,36038149	23,2202166	23,2176382	Q9DAM7
Hdac6	0,005605384	-0,255840484	0,00162094	34,90728222	34,96705502	34,90046032	34,69628469	34,65442945	34,65656196	Q9Z2V5
Cdc16	0,005605384	0,562457042	0,001610694	25,69381107	25,88764846	25,58677727	26,38376862	26,28139382	26,19044549	Q8R349
Psmd14	0,005616545	-0,324356835	0,001634192	30,86702832	30,77940892	30,77478301	30,52495969	30,40882089	30,51436917	O35593
Rab11b	0,005759228	-0,540118509	0,001683842	27,64085921	27,35749777	27,59227761	26,92637455	26,95670813	27,08719639	P46638
Rps6	0,005896123	0,555182077	0,001732194	29,0012063	29,23582861	29,33039775	29,74059708	29,6825937	29,80978812	P62754
Rpl31	0,006119507	0,368127183	0,001806465	31,4364372	31,32849439	31,40654195	31,65565603	31,8248701	31,79532896	P62900
Rab14	0,006191927	-0,542552387	0,001836589	27,01116002	27,02009183	27,104696	26,64306348	26,55313697	26,31209024	Q91V41
Cox5b	0,006214969	-0,607372796	0,001852201	27,55778866	27,47111515	27,45254076	26,76787145	27,11326748	26,77818724	P19536
Tpm3	0,006253294	-0,298882546	0,001872455	29,03603628	28,98019167	28,92442196	28,71200991	28,70714593	28,62484643	P21107-2
Rps17	0,00635633	0,561222592	0,001912286	30,33500673	30,4569915	30,46343734	30,78698214	31,02887649	31,12324472	P63276
Rab10	0,006417947	-0,828975087	0,001960158	27,67700761	27,96719248	27,46216998	26,73608684	26,85839825	27,02495972	P61027
Hsph1	0,006417947	-0,244511302	0,001959993	32,32528078	32,37458979	32,37448615	32,08077585	32,14391296	32,11613402	Q61699
Mat2a	0,006417947	0,352000959	0,001944432	29,20857694	29,14935667	29,15711344	29,61952157	29,42720378	29,52432458	Q3THS6
Rpl26	0,006417947	0,43204276	0,001967083	30,4237222	30,54333113	30,41043929	30,79102571	30,86489039	31,01770479	P61255
Hspa4	0,006556439	-0,231324538	0,00201879	31,19518989	31,19642163	31,1825775	30,97117309	30,9847478	30,92429452	Q3U2G2
Q8VDP2	0,006571626	-0,442547816	0,002032749	27,52223047	27,51915859	27,55610817	27,12040464	26,93618232	27,21326682	Q8VDP2
Psma4	0,006928284	-0,259083887	0,002152857	30,26863291	30,21326682	30,18754131	30,00704772	29,94921215	29,93592951	Q9R1P0
Khdrbs1	0,007336364	0,369302103	0,002290023	26,80156332	27,00849103	26,97048756	27,33116694	27,29202064	27,26526064	Q60749
Rab5c	0,007697413	-0,451502118	0,002417336	26,69895853	26,81677684	26,76458513	26,34470564	26,15261428	26,42849423	P35278
Ppp6c	0,007697413	0,601788622	0,002424468	28,01073331	28,24744269	28,39950012	28,76948028	28,76809238	28,92546932	A0A0N4SVL9
Hnrnpf	0,007834603	0,411426122	0,002478745	28,91219372	28,73940646	28,99385761	29,25162445	29,3375184	29,29059331	Q9Z2X1
Rab7a	0,007935436	-0,6742265	0,002521855	27,98109043	28,15088232	28,06891839	27,60229562	27,43284847	27,14306755	P51150

Mthfd1l	0,00811107	-0,21187232	0,002589127	32,12577138	32,11604104	32,15945522	31,92323213	31,92878334	31,91363521	Q3V3R1
Hspa9	0,008218174	-0,45721961	0,002634923	28,02189275	28,15745079	28,16082	27,81249893	27,60583147	27,55017431	P38647
Actr10	0,00822647	-1,662183078	0,002649202	25,92768611	25,45920196		24,16323137		23,89929053	Q9QZB7
Hspa8	0,008246961	0,232282459	0,002667449	36,84898727	36,80171652	36,8544549	37,10065191	37,03157379	37,06978036	P63017
Flii	0,008399129	-0,209376784	0,00272853	34,29426583	34,28167901	34,28257679	34,0939599	34,08551637	34,05091502	Q9JJ28
Gid8	0,008776023	-0,238303628	0,002863363	30,36191657	30,40649127	30,3843246	30,09156601	30,168659	30,17759655	Q9D7M1
Rpl28	0,008935084	0,449556403	0,002927881	28,3681552	28,2910546	28,30351942	28,92773131	28,61775391	28,7659132	P41105
Rpl32	0,009262011	0,889364542	0,003048091	27,23671831	26,7933999	27,43944248	27,93954891	28,04635202	28,1517534	P62911
Jak1	0,009305168	-0,33685722	0,003075437	26,24575753	26,30806734	26,31860363	25,9652999	25,84579821	26,05075872	P52332
Krt8	0,009335261	0,254211875	0,003098568	32,38969318	32,40225286	32,44312358	32,641234	32,73366079	32,62281046	P11679
Eef2	0,009496984	0,346284634	0,003165661	29,07640523	28,98744748	28,99229152	29,25682904	29,37268681	29,46548227	P58252
Prps2	0,009657631	-0,697918599	0,003235873	28,21155051	28,24431541	28,18106132	27,65642344	27,20256463	27,68418337	Q9CS42
Psma5	0,009657631	-0,345740055	0,003246492	29,54170755	29,58050132	29,38337725	29,13975415	29,11453393	29,21407788	Q9Z2U1
Morf4l2	0,009723825	0,609507928	0,003282478	27,06146975	26,96939003	27,38046952	27,64127276	27,77492436	27,82365597	A2AEB5
Vars	0,009855426	0,245798024	0,003340822	30,40649127	30,39427532	30,3545794	30,69569375	30,60681996	30,59022636	Q9Z1Q9
Rhoa	0,00989165	-0,455939944	0,003367073	27,77178	27,81115182	27,77077236	27,5202832	27,25790817	27,20769297	Q9QUI0
Rpl18a	0,00994761	0,719360258	0,003406935	27,24843837	27,45677614	27,11951443	27,78849203	27,95454662	28,23977106	A0A1D5RLW<br>5
Rpl36	0,00994761	1,42640869	0,003414222	24,98923789	25,68268411	26,08561704	26,91684128	27,06620024	27,05372358	Q6ZWZ4
Shcbp1	0,010324901	1,341469392	0,003572882	23,76144472	24,37345038	24,7905939	25,70120809	25,50896718	25,73972191	Q9Z179
**Stub1**	0,010324901	1,791093373	0,003561865	24,58198828	23,32592366	24,46207727	26,046914	25,72650423	25,9698511	Q9WUD1
Zw10	0,010437184	-0,633367599	0,003641221	26,47029296	26,78593831	26,53399279	25,95568875	25,77868857	26,15574394	O54692
Rpl18	0,010437184	0,666792341	0,003637014	26,6707416	26,45935832	26,86632094	27,46318396	27,3959101	27,13770382	A0A1B0GSF<br>7
Ivns1abp	0,010601182	-0,309632241	0,003728382	30,85971679	31,03524979	30,98236953	30,70619093	30,60920013	30,63304832	Q920Q8
Cct6b	0,010601182	0,458377267	0,003716852	28,55811722		28,49663256	29,00554943		28,96595488	Q61390
Trappc5	0,010804426	-0,487051598	0,003815122	25,80781537	25,76405356	25,97167195	25,33684621	25,21963177	25,5259081	Q9CQA1
Gid4	0,010934229	-0,366708932	0,0038764	27,07653265	27,22976864	26,9699389	26,70635563	26,7573542	26,71240357	Q9CPY6
Luc7l3	0,011049548	-0,615761513	0,00393289	24,06693926	24,13296719		23,56627799	23,40210543		Q5SUF2
Cryab	0,011573899	0,457900564	0,004135871	30,33106013	30,08588038	30,39570586	30,78308339	30,7599431	30,64332157	P23927
Riok1	0,011593494	0,30729897	0,004159248	28,82832479	28,65454682	28,76840793	28,99158892	29,10319575	29,07839178	Q922Q2

Pfdn2	0,011714971	0,500107865	0,004219375	26,97869248	27,14559621	27,14004681	27,39884805	27,61757878	27,74823227	O70591
Hist1h2bp	0,012446622	0,614205018	0,004500473	28,94993568	28,89585167	28,93649125	29,24773694	29,67695718	29,70019953	Q8CGP2
Rbm3	0,012921376	-0,31469204	0,004690387	30,00718142	29,99765776	30,12102253	29,81421162	29,73563504	29,63193893	O89086
Cap1	0,013012261	-1,502374921	0,004760135	25,33360852			23,94377965	23,71868755		P40124
Rpap3	0,013012261	1,568368138	0,004742834	23,49597256	24,37060595	24,69822778	25,98598296	25,5112918	25,77263595	Q9D706
Lrrfip2	0,013349712	-0,18397686	0,004947748	34,32576328	34,32623222	34,30810098	34,11186626	34,14201162	34,15428802	A0A0G2JEP 4
Rpsa	0,013349712	0,495417972	0,004936474	29,2471031	29,18487243	29,15015602	29,90813269	29,64567668	29,5145761	P14206
Sf3b2	0,013349712	0,514881104	0,004959003	24,89994992	25,14148976	25,14117789	25,40769099	25,7370932	25,5824767	Q3UJB0
Rad51	0,013349712	2,290515655	0,004930995	24,71816493	23,97567631	22,83000819	25,84783023	26,22460792	26,32295825	D6RCK1
Trip13	0,013726257	-0,260918582	0,005118265	28,59992175	28,43256974	28,52697526	28,28568569	28,24916207	28,24186324	Q3UA06
Stk38	0,013743189	0,178893776	0,00514399	35,2341004	35,21048985	35,20354217	35,39624194	35,38586786	35,40270394	Q91VJ4
Mob1a	0,01416327	-0,256214607	0,005321229	29,0585053	29,06686743	28,94976875	28,75897479	28,71904674	28,82847612	Q921Y0
Tceb1	0,014490878	0,361964743	0,00546478	27,79606427	28,05356823	27,97415819	28,28581795	28,25309085	28,37077612	A0A087WQE 6
Rpl14	0,014539728	0,282078759	0,005503739	27,40632907	27,32911486	27,27479871	27,65690074	27,52917423	27,67040395	Q9CR57
Ppp2r5a	0,014542534	1,803247136	0,005525341	24,48193842	25,97217611	25,62240467	27,30243031	27,00281639	27,18101391	Q6PD03
Psmd4	0,014681842	-0,22550992	0,005599007	30,02128386	29,89427664	29,99401952	29,73864974	29,7539482	29,74045232	O35226
A0A075B5 P4	0,015811113	-0,334828574	0,006051994	30,98942495	30,89749712	30,98223351	30,74815228	30,49809835	30,61841922	A0A075B5P4
Psmb4	0,016265626	-0,285783507	0,006248941	29,13423116	29,08914288	28,99320978	28,79989728	28,8607086	28,69862742	P99026
Psmb1	0,016471811	-0,379767022	0,006393606	29,7006791	29,40764642	29,54408694	29,23331624	29,18023148	29,09956368	O09061
Edf1	0,016471811	0,21731292	0,006395409	29,52622908	29,54734532	29,47726672	29,6720071	29,77944024	29,75133254	Q9JMG1
Cbx3	0,016471811	2,231648626	0,006397949	23,87287732	25,88636975	24,78198906	27,14666469	27,17131029	26,91820704	Q9DCC5
Xpo5	0,016818238	-0,571791266	0,006556262	26,04282946	26,0454149	25,91777469	25,16115649	25,65436244	25,47512632	Q924C1
Prpsap1	0,016910843	-0,458780116	0,006616248	26,55759879	26,6869903	26,55682444	26,05442762	26,01143731	26,35920826	Q9D0M1
Ubap2l	0,016959565	-0,226719537	0,006707173	27,28586204	27,15923264	27,18593585	26,99894915	27,00678028	26,94514249	A0A0H2UH1 7
Cnn2	0,016959565	0,362329098	0,006700518	26,93898835	27,00238721	26,80747147	27,14248339	27,34494263	27,34840831	Q08093
Rpl21	0,016959565	0,620489676	0,006667558	28,056517	27,88180367	27,55471847	28,56409127	28,47908009	28,3113368	Q9CQM8
G3bp1	0,017009597	0,543576054	0,006750984	27,23343054	27,22527011	27,03296652	27,45301197	27,83068376	27,83869959	P97855
D8Ertd738 e	0,017378784	0,342470322	0,006922058	28,01600494	28,18593585	28,13980293	28,32997025	28,48366124	28,5555232	Q8R1F0

Igkv1-115	0,017612954	-0,198503901	0,007040206	31,4893673	31,46684395	31,40034532	31,28537703	31,22549997	31,25016787	P01631
Erh	0,017625048	-0,271463197	0,007119466	33,00833407	33,07381925	32,99054115	32,69380525	32,86350642	32,70099321	P84089
Hnrnpu	0,017625048	0,284422481	0,007079419	28,46353479	28,4170759	28,27892418	28,61034474	28,67296814	28,72948943	Q8VEK3-2
Lsm4	0,017625048	0,358758277	0,007119723	27,50582149	27,77108732	27,77058335	28,02152215	28,03301905	28,06922578	Q9QXA5
Rab1b	0,017640406	-1,591026826	0,007150843	27,31734471	27,32620275	27,27444328	26,36051491	25,88108016	24,90331518	Q9D1G1
Asna1	0,017893482	0,409783862	0,007278705	29,01573916	29,1859831	29,35844131	29,52395085	29,65061507	29,61494922	O54984
Snrpb	0,018291253	-0,260790212	0,007466345	32,53436021	32,34931562	32,44620866	32,22719403	32,16976429	32,15055553	P27048
Psmd6	0,018781871	-0,24283873	0,007719667	30,82449079	30,83447122	30,9333286	30,60884776	30,69436583	30,56056083	Q99JI4
Psmd2	0,018781871	-0,176238389	0,007719384	31,34715191	31,29854641	31,36264824	31,14449047	31,17860599	31,15653493	Q8VDM4
Itga3	0,018870275	-0,293100469	0,007782656	27,47838588	27,66037342	27,45795043	27,2828612	27,18574686	27,24880027	Q62470
Anapc4	0,018996953	0,59738704	0,007861733	26,02074889	26,43610952	26,44736301	26,91968515	27,03674376	26,73995362	Q91W96
Hspa4l	0,019113168	-0,245084091	0,007936824	31,16907732	31,20623791	31,03492195	30,86621775	30,89655915	30,912208	P48722
Map2k1	0,01911844	0,321443256	0,007966017	26,85875393	26,87396578	26,93111766	27,34916796	27,08233131	27,19666786	P31938
Rpl9	0,019970696	-0,284036522	0,008361199	28,78453496	28,64357962	28,77824992	28,50301785	28,34878818	28,50244889	P51410
Polr2b	0,019970696	1,625699445	0,008377538	25,005158	23,97316323	25,50303302	26,56133317	26,33111567	26,46600375	Q8CFI7
Ddx1	0,020268217	-0,38359883	0,008530972	28,79869284	28,95762164	28,79624997	28,51754511	28,29740981	28,58681305	Q91VR5
Zwilch	0,020463848	-0,326973246	0,008642218	27,62644876	27,64753056	27,57535262	27,43888752	27,27106231	27,15846238	H3BJ52
Hmga1	0,022068363	0,448613089	0,009351001	26,54780862	26,78194296	26,93303306	27,3087791	27,18697488	27,11286993	P17095
Zranb2	0,022162181	-0,303809246	0,009422057	27,48631089	27,52155671	27,37252077	27,06978916	27,11684047	27,28233099	D3Z4U0
Pin4	0,022484105	-0,306473185	0,009622435	27,47908009	27,42566411	27,47514178	27,0006692	27,20134981	27,25844742	Q9CWW6
Ppp6r1	0,022484105	1,551757891	0,009614505	24,79590935	23,3203732	24,25701291	25,42306896	25,79287884	25,81262133	Q7TSI3
Ipo9	0,023803479	-0,524411949	0,010220703	26,86147789	26,47254124	26,73802153	25,96382373	26,27366102	26,26132007	E9QKZ2
Rplp2	0,02396626	0,555345512	0,010324448	26,87290967	27,34384201	27,31103358	27,74912788	27,61939912	27,82529481	P99027
Ranbp1	0,02509549	0,289892655	0,010846356	26,47968148	26,28144688	26,31589473	26,57301344	26,73531224	26,63837537	P34022
Actr1b	0,025297349	-0,509397029	0,010969331	27,62219518	27,78861649	27,68465157	26,90357187	27,34756379	27,31613649	Q8R5C5
Nudt21	0,025605608	-0,323749932	0,011167391	32,06229999	32,13016708	32,03262503	31,60005472	31,75907006	31,89471753	Q9CQF3
Ywhah	0,025605608	0,279536592	0,011175329	27,97951041	27,80581225	28,01797022	28,25507375	28,24322607	28,14360283	P68510
D10Wsu52e	0,025879776	-0,210724535	0,01133154	29,72526941	29,77890785	29,76531256	29,6277537	29,53652944	29,47303307	Q99LF4
Eef1d	0,025948572	0,200237506	0,011398314	31,275365	31,2215298	31,26561834	31,53422104	31,41367063	31,41533397	Q80T06

Psmb6	0,026135299	-0,335911042	0,011564133	28,77545822	28,56790317	28,86761753	28,41888608	28,33445273	28,44990699	Q60692
Mtap	0,026135299	-0,241421794	0,011521866	29,26789659	29,31194305	29,36571721	28,96873112	29,14481863	29,10774172	Q9CQ65
Snrpd2	0,026135299	-0,174477834	0,011627993	32,30399836	32,3135981	32,39728801	32,16635607	32,14761716	32,17747775	P62317
Abce1	0,026135299	0,242880287	0,011610388	28,33253337	28,1637976	28,2284389	28,41864486	28,48922372	28,54554216	P61222
Map3k7	0,026407898	0,201929563	0,011786576	28,38775868	28,50589718	28,49174853	28,61925918	28,70470778	28,66722613	Q62073
Calu	0,026772187	-0,654794106	0,011986982	27,92150229	28,03988392	27,75066191	26,92331715	27,26597595	27,55837271	O35887
Atp5o	0,02761529	-1,878854774	0,012403478	26,45932705	26,35306119	26,36862981	25,39559176	23,42281754	24,72604444	Q9DB20
Rap1b	0,027836105	-0,438228163	0,012541974	27,31708589	27,15218874	26,90472061	26,74021104	26,57272439	26,74637531	Q99JI6
Mta2	0,028699805	0,209336494	0,012980551	29,48241153	29,475625	29,53146282	29,73444031	29,61412431	29,76894421	Q9R190
Ccnd1	0,028699805	0,50904243	0,0130122	26,59988629	26,28580913	26,68538701	26,85638107	27,03590523	27,20592342	P25322
Surf4	0,03044302	-1,283645927	0,013845554	25,67758567	25,32943997	26,45092176	24,51247111	24,95391771	24,1406208	Q64310
Farsa	0,030638895	-0,314969889	0,013977914	25,64872402	25,73619009	25,7651418	25,37268681	25,26902992	25,56342952	E9PWY9
Ccdc124	0,031026935	-0,242273616	0,014218284	28,54078425	28,49579429	28,59423694	28,21066837	28,28011969	28,41320657	Q9D8X2
Eif4b	0,031026935	-0,207128343	0,01424259	36,96182618	36,93703961	36,89430646	36,63328114	36,74585061	36,79265548	Q8BGD9
Calm1	0,031125291	-0,287613945	0,014331702	31,16104834	30,90906316	31,14588769	30,7561296	30,77839093	30,81863683	P0DP28
Mdh2	0,031901728	-0,564662566	0,014773637	26,13965658	26,67424853	26,3010176	25,93642947	25,63178632	25,85271923	P08249
Ipo11	0,031901728	0,460890962	0,014779332	25,56459284	25,56082588	25,21763649	25,83035737	26,09008738	25,80528335	Q8K2V6
Eif3k	0,032320671	-0,258663206	0,015019069	29,586133	29,71842627	29,6208852	29,41284329	29,47291691	29,26369465	Q9DBZ5
Ankfy1	0,033562544	0,182209081	0,015643559	31,50706054	31,46577417	31,48072752	31,74350514	31,64761633	31,609068	Q810B6
Ybx3	0,034109259	0,17161056	0,01594656	29,96719248	30,04557113	29,92209804	30,16170912	30,13809459	30,14988962	Q9JKB3-2
Gm17669	0,034295859	0,522811016	0,016082239	28,32212449	28,45614946	28,60027631	28,69421637	29,04163421	29,21113272	F6QL70
Tnpo3	0,034711115	-0,58257947	0,01632599	26,14384607	25,85450375	26,1176729	25,78989776	25,34523034	25,23315622	Q6P2B1
Maged1	0,034806822	1,432671425	0,016420168	23,55863966	25,10790906	24,1313516	25,97692416	25,50291166	25,61607877	Q9QYH6
Hmgb1	0,034951902	-0,426308462	0,016537976	27,27976557	27,34299482	27,33355734	26,67707484	26,85210008	27,14821744	A0A0J9YUD8
Ilk	0,035053573	0,905397024	0,016635594	25,61093606	26,45417364	26,42569612	27,2761308	27,04708047	26,88378563	O55222
Klhl22	0,035533081	0,233913599	0,016913345	28,81900245	28,72256862	28,61104867	28,93755796	28,90962116	29,00718142	Q99JN2
Setx	0,038047462	-0,312958492	0,01819925	28,60158741	28,41300476	28,61929417	28,30417248	28,30977671	28,08106167	A2AKX3
Alyref	0,038047462	0,212550564	0,018229625	28,51171405	28,55629093	28,61030954	28,87302706	28,74364958	28,69928957	O08583
Upf1	0,038047462	0,363010242	0,01827138	27,61862926	27,91130814	27,57549689	28,07244945	28,15735441	27,96466114	Q9EPU0-2

Cfl1	0,03931021	-0,218946973	0,018933307	28,86097494	28,86614404	29,00477291	28,74310382	28,72361048	28,60833667	P18760
Dhx35	0,039589578	-0,226288813	0,019123779	26,78219299	26,77391891	26,88390214	26,51975849	26,54229816	26,69909096	A2ACQ1
Capzb	0,039721572	-0,239665656	0,019243643	28,58684883	28,55493799	28,66302338	28,40653182	28,44525669	28,23402472	P47757-2
Ywhab	0,040790878	0,222107047	0,019819296	27,99625745	28,07239834	28,05935659	28,31717217	28,33458059	28,14258076	Q9CQV8-2
Calr	0,040961661	0,148897525	0,019960132	26,57974637	26,57960252	26,57333133	26,67801571	26,77693316	26,72442392	P14211
Frg1	0,041074411	-0,150431541	0,020073088	30,27918994	30,30791105	30,24773694	30,08017226	30,14418654	30,15918451	P97376
Hnrnpa2b1	0,041306709	1,144579711	0,020244955	25,20362909	23,96365015	24,92818328	26,08768199	25,61955305	25,82196662	O88569-3
Eif2b2	0,042329409	-0,580623759	0,020805981	27,4104595	27,73395568	27,79810569	27,29815307	26,74278269	27,15971384	Q99LD9
Nap1l1	0,042575921	0,600427274	0,020987283	26,65321465	26,53338392	25,9940573	26,87502111	27,00142108	27,1054955	Q3TF41
Prpf31	0,042623045	-0,153483305	0,021070715	31,72736473	31,6724034	31,75142832	31,52673279	31,60659937	31,55741437	Q8CCF0
Rpl15	0,042980021	0,588253923	0,021307892	26,18328766	26,83364215	26,64828516	27,17693112	27,26391847	26,98912715	Q9CZM2
Rps29	0,043316179	0,187347618	0,021535727	32,33639082	32,21228135	32,34337082	32,48934337	32,53315367	32,4315888	P62274
Prkci	0,043755487	-1,571262604	0,021865262	25,77389377	25,55818292	25,35618839	23,24324155	23,67106168	25,06017403	Q62074
Eif1ax	0,043755487	-0,274013785	0,021877744	28,32992749	28,47981251	28,30412896	28,06707266	27,99091303	28,23384192	Q8BMJ3
Vbp1	0,043900105	0,327439956	0,022012058	27,26552892	27,33790115	26,99668846	27,44379546	27,55983182	27,57881112	P61759
Dync1li2	0,044223681	-0,883431321	0,022236766	24,99576163	25,30938642	24,77660692	24,29824049	24,52318815	23,61003238	A0A1D5RMC1
Cad	0,044330017	0,26019829	0,022352848	29,17949608	29,20153677	29,01855397	29,30704248	29,3579801	29,51515911	E9QAI5
Abcd3	0,04591298	0,444217968	0,023215886	27,48784469			27,92116175		27,94296357	P55096
Asf1b	0,046859011	0,787444909	0,023760431	24,69639412	25,21793543	24,43623244	25,48096051	25,89873718	25,33319904	Q9DAP7
Isyna1	0,046971724	-0,229182723	0,023950272	26,32049856	26,11729639	26,21887463	26,03584232	26,03288247	25,90039661	Q9JHU9
Mcm5	0,046971724	-0,205205392	0,023948412	25,55660522	25,53287881	25,54904338	25,41802553	25,2172667	25,38761901	P49718
Fhl2	0,047370168	-0,324287203	0,02422034	27,28489186	26,98424529	27,33381322	26,89689112	26,81237652	26,92082113	O70433
Nsdhl	0,047372637	-1,224675625	0,024288513	25,49774441	25,57265212	25,88075329	24,33469992	23,72358164	25,21884139	Q9R1J0
Vim	0,048537116	0,349177267	0,02495411	27,7128627	27,54568944	27,65116274	28,2041517	27,94145316	27,81164182	P20152
Rsu1	0,049214351	0,500234088	0,025371805	26,80168665	26,87970215	27,29955111	27,45309049	27,33756093	27,69099076	Q9D031
Gsn	0,050802466	0,137921822	0,026262292	31,27080619	31,24269245	31,17599187	31,36285722	31,36635315	31,3740456	P13020-2
Coprs	0,053861868	-0,179523613	0,027919923	28,94271194	28,86050141	28,88296986	28,62867514	28,79584759	28,72308964	Q9CQ13
H2-Ke2	0,054842668	0,403977832	0,028505793	26,91718284	26,78992263	26,77366744	27,18536879	27,50430669	27,00303093	G3UYF9
Pla2g4a	0,054861633	0,494672707	0,028593139	25,72489225			26,27591774	26,16321217		Q9DBX5

Tceb2	0,058517984	0,228355445	0,030581432	27,89180224	27,77850059	27,67640241	28,02385002	28,09113721	27,91678435	P62869
Ccdc97	0,058946925	0,376396789	0,030888855	25,22898012	25,55168573	25,49649542	26,00330979	25,64380666	25,75923519	Q9DBT3
Psma7	0,059543763	-0,203142677	0,031360238	29,71657958	29,50349182	29,60084342	29,464392	29,38699854	29,36009624	Q9Z2U0
Nisch	0,059543763	1,012397172	0,031369808	24,86322573	23,52528088	24,33029113	25,0841982	25,29165187	25,3801392	Q80TM9
Hypk	0,062225207	-0,260170028	0,032870378	26,47037054	26,74239723	26,66207267	26,45304338	26,26647646	26,37481052	Q9CR41
Ddx3x	0,06262172	0,186261699	0,033168284	28,80900609	28,69348542	28,60102059	28,91795106	28,8650822	28,87926394	Q62167
Ftl1	0,063780062	0,757774501	0,033871898	26,30772001	26,13029844	25,41593231	26,86679256	26,81836256	26,44211914	Q9CPX4
Rbm10	0,064273177	-0,219684419	0,034224559	27,92716615	28,17402817	28,13413322	27,89891012	27,85364746	27,8237167	Q99KG3-3
Mms19	0,064566618	0,321764171	0,034472008	26,69975289	26,86502318	27,03412173	27,23196689	27,02970595	27,30251747	Q9D071-2
Zwint	0,064601218	1,628189235	0,034581726	24,42997141	23,86833324	26,06591274	26,34062001	26,53088636	26,37727872	Q9CQU5
Aldoa	0,066265373	0,166786526	0,035566161	25,88969204	25,862164	25,76207744	26,04051114	26,04347625	25,93030566	P05064
Pitpnb	0,067038861	0,397012054	0,036075997	26,12931604	26,59818316	26,44878126	26,95460209	26,68257696	26,73013757	P53811
Rnh1	0,067413807	0,167431831	0,036372986	28,00955922	28,07525789	28,18319299	28,23867828	28,29819678	28,23343054	Q91VI7
Flna	0,070534853	0,149150615	0,038156566	31,4256241	31,37098393	31,38118153	31,48409358	31,51709451	31,62405331	B7FAU9
Serbp1	0,070604488	-0,467804067	0,038293959	28,1517534	28,38492152	28,1330064	27,48592719	27,68759108	28,09275085	Q9CY58-2
Zmynd19	0,074725327	-0,966066352	0,040634535	25,38386748	25,32467582	25,84438607	25,13136878	24,67673195	23,84662958	Q9CQG3-2
Actr1a	0,07499997	-0,235375505	0,040889814	30,7599431	30,83605267	30,65010145	30,61227971	30,56274563	30,36494536	P61164
Ahsa1	0,078381951	0,202735748	0,042844371	26,38231794	26,49539785	26,40798266	26,68311263	26,49249747	26,7182956	Q8BK64
Sugt1	0,07863506	1,843839226	0,04309379	26,45688578	24,01682223	26,36519574	27,53155578	27,34688782	27,49197784	Q9CX34
Bag3	0,079180894	1,069802229	0,043504757	25,14478101	24,37460385	25,92522029	26,23138102	25,99677465	26,42585616	Q9JLV1
Rbms2	0,081117728	1,72306322	0,044683494	25,67458908	23,14629572	24,04693501	26,32541375	25,85771035	25,85388537	E9Q7G6
Actr2	0,081321442	0,352131513	0,044910571	25,79605189	25,90508801	25,5039429	26,14483808	25,90302016	26,21361911	P61161
Rac1	0,082415206	-0,574750206	0,04586383	25,25648715			24,79615094	24,56732296		P63001
Psmd5	0,082415206	0,148501497	0,04585346	28,04520657	28,07831542	28,16216549	28,27559811	28,28356784	28,17202602	Q8BJY1
Txndc9	0,082415206	1,001643204	0,045737839	25,50960154	24,17467144	24,25810269	25,47339313	25,78366731	25,69024485	A0A0A6YWP9
Spata5	0,083614663	0,261152504	0,046649423	28,29421379	27,95941929	28,15363892	28,30051148	28,40932684	28,48089119	A0A0G2JFY0
Hspe1	0,084763757	-1,978726105	0,047410237	26,40773961	26,58003401	26,74791227	24,79803264	26,01407957	22,98739536	Q64433
Eif1	0,085236273	-0,484008351	0,047938543	28,42306095	28,0104132	27,81207044	27,8154948	27,5856317	27,39239303	P48024
Tnrc6b	0,085236273	-0,286075391	0,04780813	26,64801081	26,34118014	26,39232751	26,12089897	26,07218364	26,33020967	Q8BKI2

Psmd7	0,085236273	-0,147575345	0,048035696	30,57596567	30,45689361	30,43181294	30,38946241	30,31893973	30,31354405	P26516
Rpl27	0,085925143	0,428988589	0,048545279	26,76660837	27,3243153	27,25628918	27,44047255	27,70081136	27,49289471	P61358
Capza1	0,086641635	-0,20554391	0,049072452	28,13462286	27,90339948	27,88384389	27,74374587	27,81543372	27,74605491	Q5RKN9
C0HKD9	0,086943929	0,16027564	0,04948927	29,43533469	29,40472636	29,3983792	29,48901312	29,68460142	29,54565262	C0HKD9
Btf3	0,086943929	1,415608569	0,049457665	25,39014743	23,41282085	23,873754	25,47233982	25,35157929	26,09962889	Q64152-2
Cdc42	0,087977636	-0,268413519	0,050201928	26,7568454	26,71515618	27,0471845	26,62268393	26,46620606	26,62505553	P60766
Csnk2a1	0,089026615	0,304528333	0,050926241	26,61498432	26,24776863	26,62380046	26,71056558	26,89977455	26,78979828	Q60737
Txn	0,089770698	-0,233290272	0,051478677	28,81592228	28,89414651	28,67791493	28,53255931	28,44600648	28,70954711	P10639
S100a10	0,089793343	-0,301659701	0,05161849	28,25300065	28,12386138	28,03107419	27,76439531	28,06512182	27,67343999	P08207
Clns1a	0,093550014	-0,149617525	0,053910178	31,18789522	31,25535519	31,2931286	31,1823999	31,07928229	31,02584423	Q923F1
Fasn	0,093926233	-0,11447608	0,054259646	29,27874699	29,28890063	29,19340493	29,12831822	29,13997365	29,14933244	P19096
Eif3h	0,094463432	-0,1517122	0,0547034	30,87312976	30,88066573	30,87004551	30,62278865	30,81993131	30,72598444	Q91WK2
Tubb3	0,095640068	2,079512598	0,05551987	24,49121303	23,28679588	26,62128706	26,73285659	26,9560434	26,94893377	Q9ERD7
Snrpd1	0,095871945	-0,239366158	0,055789889	31,85385563	31,9122794	31,60071938	31,49214024	31,51488644	31,64172926	P62315
Znf706	0,09675114	-0,171285619	0,056438165	30,94489124	31,05594841	30,95629271	30,69453189	30,87078045	30,87796316	Q9D115
Ranbp10	0,097585662	0,101722102	0,057062802	29,80079225	29,80856122	29,75907006	29,90798949	29,89951528	29,86608507	Q6VN19
Sept2	0,098884309	-0,212442347	0,057961848	27,61925918	27,66050944	27,78631231	27,32979921	27,49906838	27,59988629	E9Q3V6
Gtl3	0,099668489	0,832528993	0,058562276	25,17056153	26,18359056	24,89453591	26,36646363	26,20071395	26,17909739	Q8BTU1
Gstp1	0,100413712	-0,680968244	0,059141974	25,67438324	25,69330594	25,44493298	25,4885497	24,40244735	24,87872039	P19157
Polr1c	0,100679757	1,116264452	0,059440873	23,80242224	25,44097939	25,369306	25,9678521	26,04155589	25,95209299	G3UXX5
Psma6	0,103405576	-0,170712556	0,061196238	30,10462103	30,25788569	30,1061198	30,00584386	30,0614955	29,88914949	Q9QUM9
Kars	0,103646338	-0,126409105	0,061635018	29,4231812	29,51793926	29,49575617	29,28636891	29,39145096	29,37982945	Q99MN1
Eif3i	0,103646338	-0,098864282	0,06177491	31,79087039	31,74370575	31,80720275	31,6910823	31,67942587	31,67467788	Q9QZD9
Tbc1d15	0,103646338	0,167633058	0,061777902	26,95537836	26,96212536	26,99776542	27,2030316	27,00131369	27,21382302	Q9CXF4
Trim21	0,104268704	-0,101160508	0,062296132	34,14140266	34,15526865	34,09474649	34,04457813	34,00261187	34,04074627	Q3U7K7
Eif3a	0,105989093	-0,159069234	0,063473694	28,68849177	28,72481421	28,5831226	28,5924202	28,50956379	28,4172369	P23116
P01837	0,106047049	-0,2208986	0,063658186	31,51559151	31,2525947	31,40897269	31,03721522	31,24161325	31,23563463	P01837
Eef1g	0,106573148	0,149166183	0,064275048	32,89926316	33,02358237	32,88723664	33,06963234	33,02311966	33,16482873	Q9D8N0
Eprs	0,106573148	0,337779354	0,064229449	24,78266794	24,90113353	24,38482274	25,09156097	24,92420106	25,06620024	Q8CGC7

Ube2z	0,106771365	0,422857481	0,064545402	26,85887247	26,21983484	26,60611396	27,01318515	26,8075943	27,13261426	Q3UE37
Actc1	0,108691262	0,115453292	0,065859536	32,23054757	32,2255287	32,13879647	32,28410863	32,298164	32,35895999	P68033
Psme1	0,109940759	-1,248009084	0,066794291	26,08681183	25,93642947	25,75712526	25,14573224	25,43147822	23,45912885	G3X9K9
Dynlrb1	0,109940759	-0,466909207	0,066927214	26,17786264	26,38366976			25,62005668	26,00765729	P62627
Cltb	0,110074334	-0,390136459	0,067474945	25,86180916	25,55083484	25,55528914	25,18591611	25,02732315	25,5842845	Q6IRU5-2
Wbp5	0,110074334	-0,139888256	0,067192484	27,30938642	27,20936739	27,21502739	27,10229485	27,02749299	27,1843286	Q9DD24
Srsf3	0,110074334	0,332067512	0,06745135	26,18587915	26,11000436	26,19572964	26,59818316	26,71148487	26,17814767	P84104-2
Prmt1	0,110165763	-0,154236643	0,067686592	31,17664584	31,03577416	31,02221032	31,00898516	30,88278042	30,88015482	Q9JIF0-2
Ywhaq	0,113274337	0,151128499	0,069756513	29,16767846	29,22131083	29,25538896	29,46265756	29,25941309	29,3756931	P68254-2
Pabpc1	0,114095413	0,098243061	0,070423299	29,16394153	29,09577157	29,089446	29,21066837	29,21925787	29,21396204	P29341
Zyx	0,115703794	-0,219438166	0,071579466	29,23774421	29,09194426	29,1212696	28,79652848	28,87949767	29,11661742	Q7TQE2
Sept7	0,115949938	0,14161686	0,071895513	27,4143362	27,56056083	27,58799343	27,66112134	27,62184597	27,70477373	O55131
Dimt1	0,117087798	0,639732565	0,072766428	24,56220702			25,37537411		25,02850505	Q9D0D4
Ddx41	0,117995925	-0,191807693	0,073497462	27,60491298	27,50316954	27,34654971	27,22278532	27,39214733	27,2642765	Q91VN6
Sf1	0,122698623	-0,264779657	0,076599987	26,45298056	26,01531381	26,10387606	25,92687217	25,89629064	25,95466864	D3YVH4
Dync1li1	0,122834228	-0,761204374	0,07685814	24,72030647		24,48873355	23,48468328		24,201948	Q8R1Q8
Ewsr1	0,124135211	-0,222029244	0,077847505	28,33756093	28,31941358	28,05491403	27,97907424	27,92897389	28,13775267	Q5SUT0
Tmod3	0,124453397	0,096624689	0,078222827	37,37503018	37,36772702	37,41447104	37,50845144	37,43937869	37,49927217	Q9JHJ0
Mapkapk5	0,128181766	0,335828178	0,080747271	27,10749231	26,59006571	26,58060913	27,02696559	27,10659409	27,15209201	O54992-2
Eif2s1	0,131946452	0,174147502	0,083305175	28,55603506	28,37832105	28,47456171	28,51386112	28,74143314	28,67606608	Q6ZWX6
Pcbp2	0,132867398	-0,212590391	0,084074286	28,56037861	28,72393591	28,45560089	28,32770236	28,51397403	28,26046785	A0A2R8VI25
Copz1	0,134642438	-0,143177107	0,085415427	27,02273946	26,99410047	27,00463899	26,74188313	26,96488144	26,88518303	P61924
Lims1	0,134642438	0,275739327	0,085577821	26,08933991	25,79953914	25,60842833	26,17224544	26,01990101	26,13237892	A0A0R4J005
Ap2m1	0,136866091	-0,736375816	0,087184473	25,19700547	25,62257223				24,67341303	Q3TWV4
Akap8	0,139801319	-1,534324375	0,089646609	26,62002871	26,49200841	26,54234244	26,12630573	25,57686673	23,34823397	Q9DBR0
Psmc3	0,139801319	0,109668491	0,08950777	29,03745089	29,07357349	29,01600494	29,22584468	29,13809459	29,09209553	O88685
Psmc2	0,139801319	0,130115725	0,089382464	30,0150479	29,96444082	29,9549071	30,01318515	30,20431498	30,10724286	P46471
Psmc4	0,140087869	0,169801232	0,090028221	29,91826392	29,98847719	29,80083852	30,06663652	30,18824904	29,96209777	P54775
Cdc23	0,143005784	0,312400816	0,092105421	26,33844533	26,18593585	26,07932299	26,73699002	26,59077959	26,21313701	G3X8W7

Fus	0,14422918	-0,142991261	0,093300797	29,56982346	29,51265614	29,40574094	29,42444321	29,25871698	29,37608656	Q8CFQ9
Phf5a	0,14422918	2,229760414	0,093185258	23,87622483	27,51968352	23,65414283	27,13936383	27,28832863	27,31163996	P83870
Psmd10	0,144262467	0,23116479	0,093526091	27,25727878	27,57260876	27,22425831	27,48385341	27,57866718	27,68511962	Q9Z2X2
Trmt112	0,145373516	-0,50843189	0,094451719	26,21152266	25,37228827	25,83185577	25,55109896	25,03684176	25,30243031	Q8VCR4
Rpl35a	0,148238651	0,66938493	0,096522625		25,19535418	25,60557718			26,06985061	O55142
Prdx1	0,149892434	-0,12757324	0,097811164	27,85239778	27,90936365	27,80562777	27,82868796	27,69703701	27,65894451	P35700
Hspbp1	0,150475524	0,128560834	0,098404191	26,56104177	26,68016398	26,74675971	26,80020595	26,74136885	26,83207315	Q99P31
Serf2	0,155405781	0,697088274	0,101847857	28,70259577	29,11951443	28,72126522	28,81802727	29,71612163	30,10049135	P84102
Got2	0,161797457	-0,358704586	0,106265279	25,37868487	25,63682771	25,64471447	25,58014906	25,01337686	24,99058737	P05202
Psmd13	0,162204124	0,089531008	0,106761471	30,09484086	30,15677601	30,17819042	30,24943337	30,24920729	30,19975965	Q9WVJ2
Hist1h1b	0,162513202	1,9053984	0,107194443	24,26120658	27,36419398	23,77680156	27,06722656	26,81017131	27,24099945	P43276
Slc25a5	0,163280793	-2,095782652	0,107931372	27,6836481	27,72760818	27,66315914	23,10150692	26,84397892	26,84158162	P51881
Bud31	0,165034533	-0,310375242	0,109323723	26,68859182	27,12109665	26,67141667	26,29519524	26,64443944	26,61034474	Q6PGH1
Bola2	0,166138402	-1,164043992	0,110289617	26,46486715	26,07412496	26,6836481	23,99222201	25,43195636	26,30632986	Q8BGS2-2
Xpo1	0,168374611	-0,366806941	0,112963192	26,10992468	26,8003294	26,19985324	26,08492806	25,91517787	26,00958057	Q6P5F9
Anxa1	0,168374611	-0,113449271	0,112501854	30,96671132	30,83590213	30,80385868	30,73481175	30,79529025	30,7360223	P10107
Cdk18	0,168374611	-0,097670858	0,112067184	39,78510657	39,70842244	39,80941884	39,71971655	39,63304426	39,65717448	Q04899
Ran	0,168374611	0,166387741	0,112949811	27,93393355	27,65955707	27,82055529	28,06573816	27,9281268	27,91934419	P62827
Snrpf	0,168374611	1,10059756	0,112814954	25,73972191	25,629259	23,75623539	26,45414225	26,10383605	25,86903067	P62307
Wdr26	0,17727776	-0,084600231	0,119186743	30,86141873	30,82737103	30,80385868	30,75214645	30,70330562	30,78339568	E0CYH4
Fam32a	0,181239673	-0,161081907	0,12210639	28,00308456	28,15214037	28,20932091	27,94832115	28,08582974	27,84714922	Q9CR80
Rpl30	0,184364612	0,156467905	0,124472153	26,38871958	26,420694	26,30059876	26,66776753	26,53017198	26,38147655	A0A2I3BQF4
Capza2	0,186600426	-0,210699201	0,126772323	27,0910363	27,15981007	27,00367437	27,09194426	26,67397907	26,85649981	P47754
Usp15	0,186600426	-0,078516777	0,126658689	31,63646646	31,56873682	31,59805889	31,53839015	31,53231778	31,49700391	Q8R5H1-5
Tab1	0,186600426	0,082353863	0,126686662	30,78089547	30,77533263	30,7018195	30,85971679	30,82820371	30,81718869	Q8CF89
Plaa	0,18797876	0,091382947	0,127974241	26,7242938	26,74483671	26,7518752	26,81689888	26,77479872	26,90345695	P27612
Syncrip	0,189433842	0,311291964	0,129232409	25,11616625	25,27166791	25,14639279	25,23432628	25,36663037	25,86714618	G3UZI2
Nampt	0,192930837	-0,59638163	0,131890572	25,37388178	25,41622232	24,45320727	24,90655668	24,37756006	24,17004974	Q99KQ4
Unc45a	0,193007851	-0,435827846	0,13221583	24,65102584	25,09099593		24,26456287		24,60580322	Q99KD5

Luc7l	0,197900448	-0,1914019	0,135846918	24,77213251		24,94619166		24,61099237	24,72452801	A0A0R4J047
Mfge8	0,199456193	-0,345826982	0,137685785	25,55405972	25,19151893	24,92777652	25,15286567	24,74888484	24,73412371	P21956-2
Hsbp1	0,199456193	-0,301406024	0,137425896		25,22615256	25,02943215			24,82638633	Q9CQZ1
Ndrg1	0,199456193	0,883332533	0,137759998	23,22977678	23,97313246	25,17661596	25,23761203	24,97189117	24,8200196	Q62433
Ppp1cc	0,207680708	1,433543856	0,143733823	23,78321	26,65485407	23,79062312	26,06484438	26,36962849	26,09484589	P63087
Psme3	0,215713601	0,318798194	0,149597992	25,39568134	26,05913995	25,6802713	25,85433729	26,20489796	26,03225193	A2A4J1
Isg15	0,217472326	0,11860551	0,151124836	29,09780699	29,31926283	29,28328081	29,36206294	29,32757389	29,36653033	Q64339
Sept9	0,217665125	0,155140915	0,151566252	24,74103446	24,99278313	24,97171579	24,98546188	25,17058465	25,0149096	A2A6U3
Tpm4	0,219023784	-0,156471496	0,15282168	28,31592063	28,31570474	28,37906512	28,16920876	28,01616439	28,35590285	Q6IRU2
Sub1	0,219755453	1,215990483	0,153642583	23,84353139	26,2985901	26,36912924	26,52061293	26,94748534	26,69112391	P11031
Nup93	0,223689932	-0,112068991	0,156709331	27,58598979	27,68772455	27,75569995	27,65027267	27,53371066	27,50922398	Q8BJ71
Nup155	0,231650387	-0,963322804	0,162613337	25,98832549	26,1239403	26,13844618	23,76347833	25,89515829	25,70210693	A0A2R8VHH1
Ide	0,232701351	-0,726337972	0,163679764		25,66795697	24,7069879	24,27113357	24,65113536		F6RPJ9
Luc7l2	0,233539909	0,107396082	0,164599456	29,43471851	29,31194305	29,31615808	29,51037523	29,36252284	29,51210981	Q7TNC4-2
Tpd52l2	0,234903712	-0,090786938	0,166224237	27,95742791	28,02923203	27,93421484	27,96002722	27,83056288	27,85792387	Q3TAI4
Eif3g	0,234903712	-0,06819534	0,165909212	31,34509073	31,3841187	31,41225781	31,32329548	31,31646022	31,29712552	Q9Z1D1
Psma2	0,235571586	-0,12239238	0,167029571	29,57513619	29,43747934	29,40570037	29,31352242	29,46326193	29,27435441	P49722
Eif3e	0,237010958	-0,117308872	0,168610559	30,5261731	30,44618401	30,33383455	30,4019224	30,31710746	30,23523518	P60229
Actl6a	0,237010958	0,302772547	0,168719665	25,51860345	25,74355329	25,51105045	26,09041048	25,48535911	26,10575524	Q9Z2N8
Arcn1	0,249114921	-0,105431205	0,177687903	28,28171217	28,27870268	28,12799857	28,20092905	28,05822142	28,11296933	Q5XJY5
Hsp90b1	0,249288766	-0,47366894	0,178516108	25,31765523	24,83504074	24,9464148	25,07450808	23,98172196	24,62187391	P08113
Fam96b	0,249288766	0,12249137	0,178190285	26,9104506	27,02422001	27,04343453	27,2071344	27,15942514	26,97901971	Q9D187
Hdac1	0,258007826	0,135549623	0,18512426	27,01977378	27,16968665	27,27479871	27,40673454	27,22499424	27,23917924	O09106
Arglu1	0,264482477	0,251982884	0,190143475	29,51615532	29,50829854	29,44559217	29,36861316	29,86565746	29,99172406	Q3UL36
Paics	0,26887002	-0,154716666	0,193677557	28,14642192	28,25939064	28,06840592	27,8186673	28,03438415	28,15701704	Q9DCL9
Anapc1	0,274414768	0,495663705	0,198059247	27,51675647	26,74226872	27,9636143	28,10414609	27,98538587	27,62009865	P53995
Hist1h1c	0,275021384	0,070720827	0,198954591	28,82189365	28,92546932	28,90727326	28,96928024	28,93255445	28,96496404	P15864
Kpna2	0,275021384	0,117182284	0,199273969	28,52121972	28,33108149	28,28515652	28,44876551	28,57401745	28,46622163	P52293
Vcp	0,280465265	-0,06931646	0,203614613	32,8811035	32,97656027	32,87609763	32,83409443	32,83823373	32,85348387	Q01853

Akap12	0,282768546	0,116756733	0,20568616	29,06643123	29,09065781	28,879965	29,09413613	29,06442812	29,22875999	Q9WTQ5-2
Pdcl3	0,285681283	0,443345721	0,208208393	24,1769685	25,06812911	25,21345224	25,42422294	25,3832083	24,98115578	Q8BVF2
Lrrfip1_1	0,286316114	-0,062535641	0,209075468	32,61894425	32,56912176	32,55691213	32,50156654	32,50649317	32,54931151	Q3UZ39
Rangap1	0,28854229	0,123190484	0,211108625	27,56216334	27,47776852	27,38590899	27,54000822	27,74829626	27,50710782	P46061
Fbxo21	0,289454741	0,222719958	0,212185044	26,26225245	26,72194965	26,53375521	26,61343945	26,60625519	26,96642254	Q8VDH1-2
Pdcd6ip	0,291644582	-0,227141511	0,214202235	26,53609956	26,80156332	26,65266776	26,28954276	26,76875495	26,25060838	Q9WU78
Pfdn1	0,295375802	0,147871261	0,217359877	26,12571473	26,21012954	26,3076158	26,54456972	26,18878667	26,35371746	Q9CWM4
Eif2b3	0,297380861	0,143371555	0,219255381	28,65935291	28,65519539	28,3248303	28,71056558	28,65775266	28,70117503	B1AUN2
Rbbp4	0,299205524	0,100198987	0,221023289	30,34318548	30,42151685	30,47276201	30,60408231	30,53704809	30,3969309	Q60972
Cnn3	0,306557525	-0,625550451	0,227320198	25,31028826	25,25263079	25,10405608	24,09339824	24,05656276	25,64036278	A0A0G2JDV 8
Mkln1	0,306557525	0,060494063	0,227135444	31,07348411	31,00269569	30,99590716	31,08550054	31,09490376	31,07316485	O89050
Otud4	0,306981605	-0,597398871	0,228068254	25,23176553	25,25360486	24,66432618	24,97747049	24,85305251	23,52697696	A0A0R4J260
Capg	0,309769378	0,1102746	0,230576924	25,1615794	25,19479081	24,95544487	25,13805551	25,2619477	25,24263567	Q99LB4
Ppil3	0,311203167	0,08860582	0,232083718	28,24929773	28,45273712	28,29311763	28,40344697	28,42786313	28,42965984	Q9D6L8
Ralb	0,313445081	1,041415225	0,234198373	22,90541247	25,81650832	23,46938816	25,09663635	25,15518419	25,06373409	Q9JIW9
Sh3glb1	0,313547115	-0,46179916	0,235160336	25,31748272	24,95500136	25,43571222	25,49223905	24,54804484	24,28251494	Q9JK48-3
Sap30bp	0,313547115	-0,08244906	0,235053533	29,35287604	29,29574701	29,26740551	29,31807777	29,20401174	29,14659186	Q02614
Hnrnpk	0,314124668	0,065559637	0,23603718	31,10293304	31,20349841	31,11551404	31,21396204	31,2298603	31,17480207	P61979-3
Ltbp1	0,318882708	-0,052708596	0,24006283	31,92415291	31,90091911	31,90605486	31,85199587	31,84494421	31,87606103	Q8CG19
Impdh2	0,325370632	0,21037895	0,245866226	26,69644016	26,27033169	26,59063684	26,50150011	26,95781534	26,7292301	P24547
Eif6	0,325370632	0,21829473	0,245799521	24,61756477	24,99683928		25,00705842	25,04393509		O55135
Gtf2i	0,325929475	0,193309108	0,246748868	25,62522279	25,30879646	25,70086427	25,9042842	25,51029599	25,80023064	G3UYJ6
Eif2b5	0,326262307	0,127404615	0,247461665	28,62637913	28,68160555	28,78428533	28,89663133	28,94148114	28,63637138	Q8CHW4
Rars	0,327237322	-0,112832312	0,248663389	26,01578169	26,08117344	25,79071817	25,80114391	25,8601284	25,88790407	Q9D0I9
Gsto1	0,329866932	-0,452278733	0,251127509	24,94851054	25,49689156	25,64457696	24,96714849	25,4724018	24,29359257	O09131
S100a6	0,334983923	0,090331524	0,255496213	31,42917096	31,62204678	31,61894425	31,6772849	31,67328834	31,59058331	P14069
Snrpb2	0,339392168	0,209196656	0,259337801	26,65921679	26,77102433			27,104696	26,74393844	Q9CQI7
Lgals9	0,344716314	-0,727019232	0,264379885	26,2139157	26,10101259	26,19676164	25,98244025	26,23288184	24,11531014	O08573-3
Plin3	0,344716314	-0,448110267	0,263963409	24,81548259	25,18835281	24,9598725	24,61110497	25,18714483	23,82112729	Q9DBG5

Rcc2	0,352440201	-0,110166149	0,271299307	26,13021987	26,12334833	25,9131701	26,00367437	26,01288689	25,8196786	Q8BK67
Adrm1	0,352440201	-0,085381373	0,270960736	29,04572734	29,17283675	28,96198742	28,95393638	28,93837149	29,03209951	Q9JKV1
Aimp1	0,352440231	-0,123865575	0,271797128	26,65635525	26,5496163	26,43288032	26,27664554	26,58218954	26,40842006	P31230
Eif4e	0,354656935	-0,085310658	0,274007547	27,60759615	27,73208025	27,76103762	27,60165825	27,70681669	27,5363071	P63073
Api5	0,354944825	0,090719063	0,27523264	25,94073656	25,75320226	25,80943549	26,01928598	25,90836751	25,847878	O35841
Cul1	0,354944825	0,564895102	0,275095253	25,03284044	23,23199297	24,23439153	24,91145672	24,75067469	24,53177884	Q3TPM3
Trp53	0,359971067	0,135234027	0,279638541	25,94590154	26,02985337			26,12311148		I7HIK9
Hist1h1e	0,364805479	0,059259027	0,283909349	29,99940624	29,98318538	30,01345141	30,02181334	30,02260719	30,12939957	P43274
Ctps1	0,364814463	-0,660084456	0,284431615	25,70128742	26,11860386	25,72163681	25,70638198	23,94874898	25,90614377	P70698
Gmds	0,365887627	-0,260646695	0,285934722	25,35312851		25,6635935			25,24771431	Q8K0C9
Eif3c	0,365887627	0,061054497	0,2863019	29,99658072	29,98644417	30,11048235	30,08423369	30,10024069	30,09219637	Q8R1B4
Vps16	0,372199211	0,600089217	0,291766331	23,53045261	25,42863799	24,83195673	25,5351059	24,92465414	25,13155494	Q920Q4
Acap2	0,384275083	-0,08485398	0,301775347	27,03401675	27,02189275	26,84744795	26,86029419	26,96697254	26,82152877	Q6ZQK5-2
Eef1b	0,385488802	0,055985132	0,304345813	31,45351247	31,42161717	31,51248661	31,5260798	31,47358473	31,55590711	O70251
Cript	0,385488802	0,07430292	0,304053337	29,77413891	29,72496054	29,90153061	29,93817516	29,85329052	29,83207315	O70333
Ipo7	0,385488802	0,154877055	0,304361921	27,21872685	27,02485407	27,10958599	27,35380157	26,99528721	27,4687093	Q9EPL8
Rps27a	0,385629384	-0,058672159	0,305017592	32,03786977	31,97298816	31,98311741	31,90501482	31,9037586	32,00918544	P62983
Rpl39	0,389339763	-0,135156391	0,30850227	29,70740927	29,49054406	29,27564251	29,39870537	29,40758565	29,26183564	P62892
Hnrnph1	0,412146894	-0,119738371	0,32715615	28,40539606	28,5673955	28,15561849	28,2233839	28,24495048	28,30086055	O35737
Mtco2	0,415480781	0,804185904	0,330389378	22,16868757	24,35330282	24,98671768	24,81279268	24,71040792	24,39806518	P00405
Eif3l	0,420349782	-0,056783623	0,334854911	30,86430006	30,82797666	30,87276293	30,87217581	30,72939219	30,79312079	Q8QZY1
Dctn2	0,422095165	0,054839079	0,336841481	27,8603534	27,95182629	27,90155938	28,00329907	27,89948646	27,97547078	Q99KJ8
Spin1	0,424044494	0,047970355	0,338996022	29,58582866	29,57183158	29,65104296	29,67402959	29,65563897	29,6229457	Q61142-2
Eif4h	0,425724124	0,662747851	0,340940083	26,05007365	24,12035881	26,43459921	26,40590726	25,95677459	26,23059338	Q9WUK2-2
Psat1	0,43268686	-0,123352036	0,347831301	26,39674313	26,42447925	26,4054691	26,12547827	26,18224597	26,54891114	Q99K85
Dynll2	0,43268686	-0,102617399	0,348245608	27,07275609	26,99020976	27,32001645	26,95071447	27,09808318	27,02633246	Q9D0M5
Ykt6	0,43268686	0,223742718	0,348349591	25,49078503	25,42380633	25,87290967	25,74005659	26,2081955	25,5104771	Q9CQW1
Tpi1	0,435158819	0,058540915	0,350954358	29,53413754	29,38841578	29,47865589	29,50182276	29,49825056	29,57675864	P17751
Fbln2	0,438432704	0,065503286	0,354213992	28,29355619	28,14807194	28,20499121	28,25403757	28,22306162	28,36603	P37889-2

Pkm	0,441672717	-0,063871717	0,357582523	28,83514919	28,69155659	28,86735242	28,7067179	28,78122387	28,71450127	P52480
Eif3f	0,441672717	0,0729555	0,358079293	29,82546162	29,72178672	29,64461134	29,86019057	29,70528474	29,84525087	Q9DCH4
Mif	0,445492632	-0,098575734	0,361805456	27,25060838	27,08598165	27,14947781	27,12070126	27,19215884	26,87748055	P34884
Psma3	0,481711264	-0,061325775	0,391900689	29,58509441	29,64765064	29,6965894	29,53664059	29,52035815	29,68835837	O70435
Kpnb1	0,484245826	-0,050645746	0,395054323	29,83293329	29,7545058	29,73267871	29,75074176	29,76200455	29,65543426	P70168
Psmd1	0,484245826	-0,039291707	0,395330632	31,59156446	31,58055523	31,5571861	31,50189867	31,54696831	31,56256368	Q3TXS7
Spg20	0,486890782	-0,613884708	0,398177631	26,38126199	26,57830727	26,62811887	24,38736452	26,67101167	26,68765782	Q8R1X6
Naa10	0,489286121	0,115404095	0,400827613	26,82140712	26,65499061	26,4723863	26,95814734	26,63339483	26,70345415	Q3V4D5
Otub1	0,491606587	0,106204078	0,40342292	26,16367286	25,81362464	25,77974079	26,03328167	25,95020274	26,09216612	D3YWF6
Hist1h1a	0,494330248	0,34526619	0,406356221	25,82262315	25,62975926	24,43106536	25,7134528	25,66947161	25,53632193	P43275
Eif2s3x	0,495750632	0,060889723	0,408224038	28,97781951	28,95737256	29,04113808	29,10966071	29,11684047	28,93249813	Q9Z0N1
Arhgap1	0,504686488	0,111072679	0,416295069	26,62839703	26,87384847	27,05693038	26,89735285	26,89896777	27,09607329	Q5FWK3
Srsf2	0,507101487	0,263433326	0,419003347	26,22066527			26,70754092		26,26065628	Q62093
Sec13	0,511579018	0,166592398	0,423425572	26,57715496	27,17255066	27,0213633	27,24390701	27,19197066	26,83496843	Q9D1M0
Lrrfip1	0,513833348	-0,046839764	0,426017197	29,62100752	29,60351647	29,59797012	29,64867945	29,52501572	29,50827965	G5E8E1
Wrnip1	0,514995609	0,076524286	0,427708218	26,20390909	26,36674708	26,06878516	26,25618118	26,36546275	26,24737025	Q91XU0-2
Flnc	0,51947453	-0,452001044	0,432783233	25,13535702	25,50551865	25,47360989	23,71995841	25,50439762	25,53412641	Q8VHX6-2
Vps33a	0,51947453	-0,114565813	0,432895442	25,20134981	25,64138302	25,33278943	25,4092459	25,28132306	25,14125587	Q9D2N9
Eef1a1	0,521759841	-0,036405132	0,435536817	34,38477489	34,32743737	34,32971101	34,29214135	34,33511325	34,30545327	P10126
Ambra1	0,523333444	0,418292292	0,437589546	23,93876407				23,972461	24,74165173	A2AH22-5
C1qbp	0,527048426	0,047933963	0,441440277	26,97891064	26,97048756	26,98771853	26,93325824	27,10349592	27,04416446	Q8R5L1
Aimp2	0,536697617	-0,404787777	0,450280205	25,60022667	25,86027051	25,77062115	24,21853689	25,77243459	26,02578352	Q8R3V2
Eif2s2	0,547960646	0,058678398	0,46050365	29,04559716	28,95704038	28,79597141	29,00471934	28,99614968	28,97377513	Q99L45
Wdr83	0,553423153	0,353622416	0,465875988	23,83268732	25,45063841	24,12428341	24,90903168	25,00436039	24,55508431	Q9DAJ4-2
Ybx1	0,555741946	0,068366104	0,468612912	27,87472804	28,05971759	27,96592737	27,88500843	28,11262141	28,10784147	P62960
Myl6	0,556380405	0,04612658	0,469937122	30,22882878	30,33841132	30,4089221	30,38226434	30,36202112	30,37025648	Q60605-2
Snrnp27	0,562177602	0,203041268	0,47562766	25,38238392	25,78021655		25,7843415			Q8K194
Phgdh	0,564480501	-0,048355383	0,478373306	31,7182466	31,57303331	31,56005968	31,62448914	31,56042417	31,52136014	Q61753
Dars	0,566739719	0,103845973	0,481088377	26,48753806	26,96960961	26,5504092	26,78081727	26,81945934	26,71881818	Q922B2

Actg1	0,572513895	-0,032150762	0,486798538	35,50554113	35,51793926	35,53133616	35,4645332	35,46581065	35,52802042	P63260
Dctn4	0,578675061	-0,032901892	0,492854607	26,63422611	26,58089661	26,64691286	26,57974637	26,56341497	26,62016858	Q8CBY8-2
Cops5	0,580150034	0,241280937	0,494930255	23,84241822	24,97041052	24,04722596	24,42950022	24,81601997	24,33837732	O35864
Ptma	0,586190142	0,342988814	0,500911068	24,78091737			24,7533298		25,49448257	P26350
Armc8	0,589025803	-0,051013744	0,504166153	28,71486151	28,73473101	28,74284692	28,78275541	28,70948137	28,54716143	G3X920
Fau	0,589394804	-0,065395592	0,50614695	30,9439836	30,84968642	31,04817245	31,03452846	30,83996119	30,77116605	P62862
Rab2a	0,589394804	0,285029092	0,50573047	24,2299078	25,72390338	25,42854215	25,43828475	25,35608761	25,44306824	P53994
Rpl22	0,590195448	0,049761397	0,507668119	29,90569631	30,12028103	30,01464894	30,05724033	30,11669178	30,01597837	P67984
Hnrnpab	0,607542231	-0,065443172	0,523447403	28,51031863	28,48895568	28,73502166	28,38784084	28,6125961	28,53752952	Q99020
Hnrnpa3	0,630008894	0,140514358	0,543694116	26,22705284	26,52523607	25,70458906	26,1928361	26,4012755	26,28430944	A2AL12
Gapdh	0,643485907	0,054804045	0,55623358	29,203195	29,06249944	29,15206782	29,35483158	29,08593102	29,1414118	P16858
Cand1	0,645546597	0,27504371	0,558926644	24,98090673	23,27738959	23,86185471	24,4724018	24,23286355	24,24001682	Q6ZQ38
Snrpd3	0,665251669	-0,031976807	0,576927295	32,12490912	32,09619271	32,23000351	32,14053446	32,12857019	32,08607026	P62320
Lrrc40	0,670741243	0,112338319	0,582635402	26,32632277	25,891246	25,96069012	26,24535859	26,42886158	25,84105368	Q9CRC8
Svil	0,676376793	-0,284849876	0,588486024	24,57155315	24,69932929	24,65463559	25,41541545	24,17835666	23,47719629	F6R6A4
Mcm7	0,68147363	0,153365405	0,593883093	25,42553607	26,33812225	26,2769472	26,29123029	25,98945202	26,22001942	Q61881
Ranbp9	0,684896565	-0,040013199	0,597833443	29,64337318	29,68446766	29,70839636	29,75690901	29,52262334	29,63666525	P69566
Dync1i2	0,689326069	-0,143219816	0,602673498	25,53255188	25,2834442	25,33439305	25,51511774	25,53501688	24,67059507	A2BFF8
Hdac2	0,694387271	-0,066768215	0,608079249	28,55859167	28,43932357	28,85566845	28,65942097	28,46688283	28,52697526	P70288
Fbxo30	0,6957069	0,289055393	0,610217493	23,98146067	23,10389803	25,11546382	24,50621507	24,58537382	23,9763998	Q8BJL1
Eif4e2	0,704648666	-0,059541808	0,619055749	27,76616603	27,96724746	28,14496446	27,86549523	27,82000742	28,01424987	Q0P688
Psmc6	0,706071365	-0,053624344	0,62130291	29,01440951	29,0868675	29,0271502	29,08382806	28,77247235	29,11125378	P62334
Gdi2	0,710517251	0,177725196	0,626218594	23,04710752	23,16983369	24,18046982	23,68688347	23,3457041	23,89799904	Q61598-2
Ube2n	0,710593175	-0,040523932	0,627289173	26,70490562	26,75021465	26,85780525	26,76141813	26,82650756	26,60342804	P61089
1810009A 15Rik	0,712322205	0,057494386	0,629821611	26,4986272	26,16246325	26,15390946	26,42265202	26,25596517	26,30886588	Q9D937
Dynll1	0,714465725	-0,042428611	0,632726002	28,78986046	28,8038125	28,89937121	28,67882168	28,74291115	28,94402551	P63168
Dhx15	0,714593014	0,255483521	0,633848041	24,42605021	26,13854383	26,17816667	25,7776105	25,77788636	25,95371441	O35286
Prune2	0,727474356	-0,057122859	0,64630137	26,75544528	27,14850839	26,96455099	27,02379715	26,85412324	26,81921568	Q52KR3-6
Prkag1	0,74685027	-0,035581698	0,669844522	26,14720834	26,05773613	25,98104687	26,10045125	26,08123441	25,89756059	O54950

Mob2	0,74685027	-0,033999713	0,667692333	28,65608242	28,47514178	28,5927766	28,62676205	28,44359788	28,55164173	Q8VI63	
Psmd11	0,74685027	-0,021498993	0,665241226	30,99145377	30,96320086	30,93768422	30,98427246	30,95185407	30,89171534	Q8BG32	
Pcbp1	0,74685027	0,03210322	0,665927647	29,65999932	29,54798876	29,4826231	29,68595505	29,55360208	29,54736371	P60335	
Rab8a	0,74685027	0,061768243	0,669793654	26,73943865	26,89203394	27,25682904	27,01094668	27,01818252	27,04447717	P55258	
Rqcd1	0,74685027	0,110023666	0,669533004	24,33973694		24,91729668	24,80469273	24,67238822		Q9JKY0	
Skp1	0,750314341	0,028609017	0,67403572	30,05878912	30,20396508	30,05568988	30,13748397	30,19413327	30,07265389	Q9WTX5	
Nudc	0,750314341	0,076347155	0,675070953	25,89001688				26,09006718	25,8426609	O35685	
Cacybp	0,752461365	-0,052989717	0,678065468	27,15961762	27,49220711	27,60300349	27,3672805	27,34045023	27,38812834	Q9CXW3	
Rnf219	0,758746172	-0,042593796	0,684800571	25,64748939	25,77602955	25,47370278	25,47286657	25,68181405	25,61475971	Q8K2Y0	
Ppia	0,760832399	0,041200446	0,687758101	26,99765776	27,13320243	26,96245637	27,2315092	26,91900314	27,06640556	P17742	
Tnpo1	0,764898809	0,019481431	0,692514317	29,20163024	29,2065989	29,22400524	29,25795311	29,26205974	29,17066583	Q8BFY9-2	
Mycbp	0,774924921	-0,03920966	0,702686157	28,85816108	28,79597141	28,84834375	28,59701109	28,94173298	28,8461032	Q9EQS3	
Q9CQE8	0,789710296	-0,042167938	0,717208644	26,55569872	26,14127536	26,35962719	26,31503095	26,33819027	26,27687623	Q9CQE8	
Ddx20	0,82272633	-0,146552043	0,748355588	25,24319949	23,94782856	25,50479576	24,68875187	24,99152946	24,57588635	Q9JJY4	
Gm16494	0,823890651	-0,17830341	0,750578347	25,79629949	26,2405992	25,97158425	24,66224178	26,16620401	26,64512693	E9Q2Z4	
Psmc1	0,827724327	0,029729418	0,755239993	29,08674098	28,81634962	28,85825002	29,04244333	28,90701533	28,90107021	P62192	
Srsf11	0,828401996	0,034674227	0,757028378		24,75062358			24,73907806	24,83151756	F6TN80	
Naca	0,828815976	0,04108718	0,758577334	25,84119768	25,8792581	25,71140609	26,11127865	25,7648888	25,67895597	Q60817	
Ddx6	0,829037779	-0,073003838	0,759951297	25,0091534	25,01746056	25,46814995	25,44988338	25,10113285	24,72473616	P54823	
Cttn	0,832574938	-0,60280091	0,764369646	29,77539543	29,81801202	26,65717341	30,57731705	24,33038954	29,53447153	Q921L6	
Dnm1l	0,833187113	0,03758577	0,766108489	26,54250481	26,74995901	26,5731146	26,81872825	26,43310325	26,72650423	Q8K1M6-3	
Nt5c2	0,843769936	0,03616285	0,777031071	28,51604257	28,25759351	28,34049268	28,4582243	28,56946133	28,19493168	Q3V1L4	
Tab3	0,845349557	-0,042098227	0,779679746	27,00227989	27,03506621	27,45520893	27,11177612	27,2459932	27,00849103	Q571K4	
Ablim1	0,845405313	-0,014207597	0,780925247	28,24022614	28,14228861	28,15281733	28,18966344	28,13824109	28,16480475	E9Q9C7	
Arpc1b	0,84558141	-0,039367793	0,783341666	25,90579958	25,96505765	25,94561136	25,66844399	26,18546332	25,84445791	Q9WV32	
Hspa1b	0,84558141	-0,01145004	0,784670885	31,96026893	31,93480536	31,93068775	31,94304045	31,92145973	31,92691175	P17879	
Dda1	0,84558141	0,088918851	0,783920796	26,79203483	26,86973672	27,31311152	26,49625159	27,18082427	27,56456377	D3YXY5	
Cdc26	0,847453263	0,055978562	0,787604869	27,91250784	27,40430002	27,30608645	27,6719565	27,35093891	27,76793458	Q99JP4	
Ehd1	0,850327553	-0,029255436	0,793879255	25,70680352	25,52572892	25,70522211	25,50130268	25,81452947	25,5341561	Q9WVK4	

Prkaa1	0,850327553	-0,024853791	0,792886677	25,23377793	25,22765883	25,16104113	25,104696	25,36646363	25,07675689	Q3TUQ7
Eif2b4	0,850327553	-0,022425131	0,792571705	28,13079921	28,02511818	28,25844742	28,08035018	28,05398246	28,21275678	Q61749
Stk38l	0,853252377	0,012952559	0,797815076	33,01921372	32,96523246	32,99853226	32,97362119	32,98199544	33,06621949	Q7TSE6
Tyms	0,854681654	-0,013566944	0,801565845	32,76370698	32,86273082	32,89395853	32,82494595	32,83580803	32,81894152	P07607
Morc3	0,854681654	0,015687955	0,800654709	30,21535147	30,22951654	30,13785037	30,1279248	30,2780823	30,22377515	F7BJB9
Ddx19a	0,856018449	-0,070034974	0,804082433		25,21619374		25,35507951	24,93723803		Q61655
Ppil1	0,856018449	0,024090404	0,805237694	26,61231487	26,59960257	26,51338678	26,76862877	26,58477193	26,44417473	Q9D0W5
Slc4a1ap	0,862059738	0,043025597	0,812138199	24,81894761	25,01758799	24,70956682	24,93260513	24,5712638	25,17131029	E9Q585
Eif3b	0,862980203	0,012907625	0,814224259	31,70976893	31,75585114	31,66046693	31,78007415	31,70820722	31,67652851	Q8JZQ9
Arl6ip4	0,8674438	-0,044968199	0,819660879	26,93427109	26,91854828	27,01116002	26,66546517	26,73013757	27,33347204	D3YWC2
Psmd12	0,868209657	-0,012623531	0,821610833	30,1779529	30,13307991	30,24377085	30,2291727	30,14126561	30,14649476	Q9D8W5
Eif3d	0,872125077	-0,012815825	0,826547919	29,9643031	30,05167682	30,03315037	30,06817525	29,93452419	30,00798336	O70194
Arpc3	0,896675634	-0,03459608	0,851081958	25,83829084	25,73941289	26,05593807	25,97572222	25,49255859	26,06157275	Q9JM76
H1f0	0,901331037	0,018638371	0,856773712	25,96939003		25,84981167		25,84933446	26,00714398	P10922
Synj1	0,902582289	-0,014255266	0,860512776	27,55464529	27,3478172	27,6007371	27,49686109	27,46785464	27,49571806	Q8CHC4
Scin	0,902582289	0,0113818	0,859579201	26,45309049	26,59917689	26,50945053	26,53335421	26,59348915	26,46901996	Q60604-2
Camsap3	0,902780293	-0,023365341	0,863251777	27,27967703	27,11465803	27,44387448	27,07989261	27,45442469	27,23379622	Q80VC9-2
Ppm1a	0,902780293	0,012749198	0,862629778	29,42958003	29,4274236	29,2227623	29,37380713	29,37471933	29,36948706	P49443
Lgals1	0,918257687	0,007580229	0,880645437	30,63088074	30,63512613	30,57875714	30,56619814	30,65429073	30,64701583	P16045
Bub3	0,918257687	0,026861246	0,879911662	26,54829383	25,92750527	26,02392402	26,20071395	26,19865486	26,18093806	A0A140LHA2
Psmc5	0,92567128	-0,006915397	0,890370256	30,0126525	30,07940946	30,06136674	30,0768639	29,9922645	30,06355411	P62196
Psma1	0,92567128	0,006186961	0,889713739	30,43021836	30,40760591	30,40100689	30,44756401	30,38360384	30,42622418	Q9R1P4
Eif3m	0,944150821	0,006890534	0,909478616	29,46919467	29,48878334	29,48245	29,40797051	29,47365246	29,57947664	Q99JX4
Atxn10	0,954116998	-0,009050057	0,920426426	29,18605396	28,99658072	29,20829785	29,24156779	29,04869216	29,07352241	P28658
Copg1	0,954741333	-0,009562526	0,923725724	28,03364926	27,70418007	27,78250548	27,81298848	27,80322743	27,87543131	Q9QZE5
Sgta	0,954741333	0,02337269	0,92242921	26,8766612	26,5903513	27,36527918	27,20694817	26,76040322	26,93505837	A0A1W2P6P1
Ago2	0,959914134	-0,014568054	0,930925676	26,71620341	26,37719596	26,69258783	26,87197027	26,44335284	26,42695994	Q8CJG0
Sept11	0,959914134	-0,010947936	0,931442105	27,45920196	27,92428036	27,7128627	27,67397907	27,68719059	27,70233156	Q8C1B7-3
Rbm39	0,960211945	0,006170377	0,933087314	27,83098592	27,83273716	27,98706794	27,93106128	27,93803491	27,80020595	Q8VH51-2

Rab18	0,962294523	-0,026893636	0,936470234	25,81479836		25,31447786	25,84328412		25,23220484	P35293
Eno1	0,967547098	-0,032679002	0,945681627	25,00611682	25,0209396	24,85190951	25,64146572	23,91059316	25,22887006	P17182
Sult1d1	0,967547098	0,013679892	0,944613678	25,86980731	25,66150195	26,03565357	26,197268	25,85562093	25,55511357	Q3UZZ6
Lars	0,967547098	0,028930756	0,94356149	24,10621467	25,02233733		24,73990213	24,44651139		Q8BMJ2
G3bp2	0,971671289	0,003901656	0,951085033	26,61076714	26,63117572	26,57260876	26,57208828	26,54281474	26,71135357	P97379-2
Mob4	0,97686368	-0,01542897	0,957547166		25,44928519	25,88220028			25,65031377	E0CYJ0
Pdap1	0,978243649	0,012749387	0,960281548	26,76547065	27,11436017	26,78880315	27,05631026	26,40548533	27,24508653	Q3UHX2
Copa	0,978634311	-0,003038745	0,962047289	28,17474255	28,22788831	28,1574026	28,25069873	28,0838027	28,2164158	Q8CIE6
Snrpa1	0,979792565	0,006982536	0,964569799	25,96181635	26,3035978	25,98979847	26,14256129	25,84680262	26,28679632	P57784
Rbbp7	0,983106271	0,002458149	0,969220589	28,91142244	28,91419144	29,0339905	28,93086396	28,90733057	29,0287843	Q60973
Arf1	0,98420462	-0,003074262	0,972088257	28,71253477	28,79519734	28,84950747	28,78606299	28,91507526	28,64687854	P84078
Sf3b5	0,98420462	0,005265812	0,973083664	26,38852253	26,18010341	26,12143265	25,9967531	26,20411438	26,50498854	Q923D4
Copb2	0,990443361	0,003107076	0,980650842	27,81769189	28,1856996	28,19704297	27,96223571	28,12094839	28,1265716	O55029
U2af1	0,991948528	-0,002012451	0,984943241	27,74829626	27,43428106	27,42085459	27,55786168	27,5597589	27,47977397	Q9D883
Rpl11	0,991948528	0,00163911	0,984102191	29,4475246	29,28865866	29,30384599	29,25824523	29,31977964	29,46692172	Q9CXW4
Prdx2	0,992142355	-0,002161196	0,986537031		24,79094184	24,63627457		24,59951745	24,82337657	D3Z4A4
P4hb	0,994433436	-0,008611548	0,990436224	24,02538224	23,82502786	24,54910215	22,7527274	24,25400152	25,36694869	P09103
Ctgf	0,994433436	0,000837554	0,993028869	29,29351234	29,30223418	29,11988542	29,09431235	29,26845444	29,35537782	P29268
Cope	0,994433436	0,000903853	0,99214699	28,22885171	28,26785196	28,04536282	28,06038779	28,25916612	28,22522414	O89079
Oxsr1	0,997999656	-0,000238173	0,997999656	25,1414118	25,10641438	25,27852546	25,01678075	25,27760354	25,23125283	Q6P9R2

Table S4. Mass-spectrometry analysis of the interactomes of CDK18. Shown are the interacting proteins of the kinase dead and phospho-mimic versions as ratio S12:K150. Protein in grey letters are protein kinases, on blue background protein phoasphatases are indicated, STUB1 is highlighted in red letters. A yellow background indicates binding of the respective protein to constitutively active CDK18, S12D, a red background indicates binding to the kinase dead version, K150R. The green background indicated the protein whose binding is significantly different from noise.

Figure S2

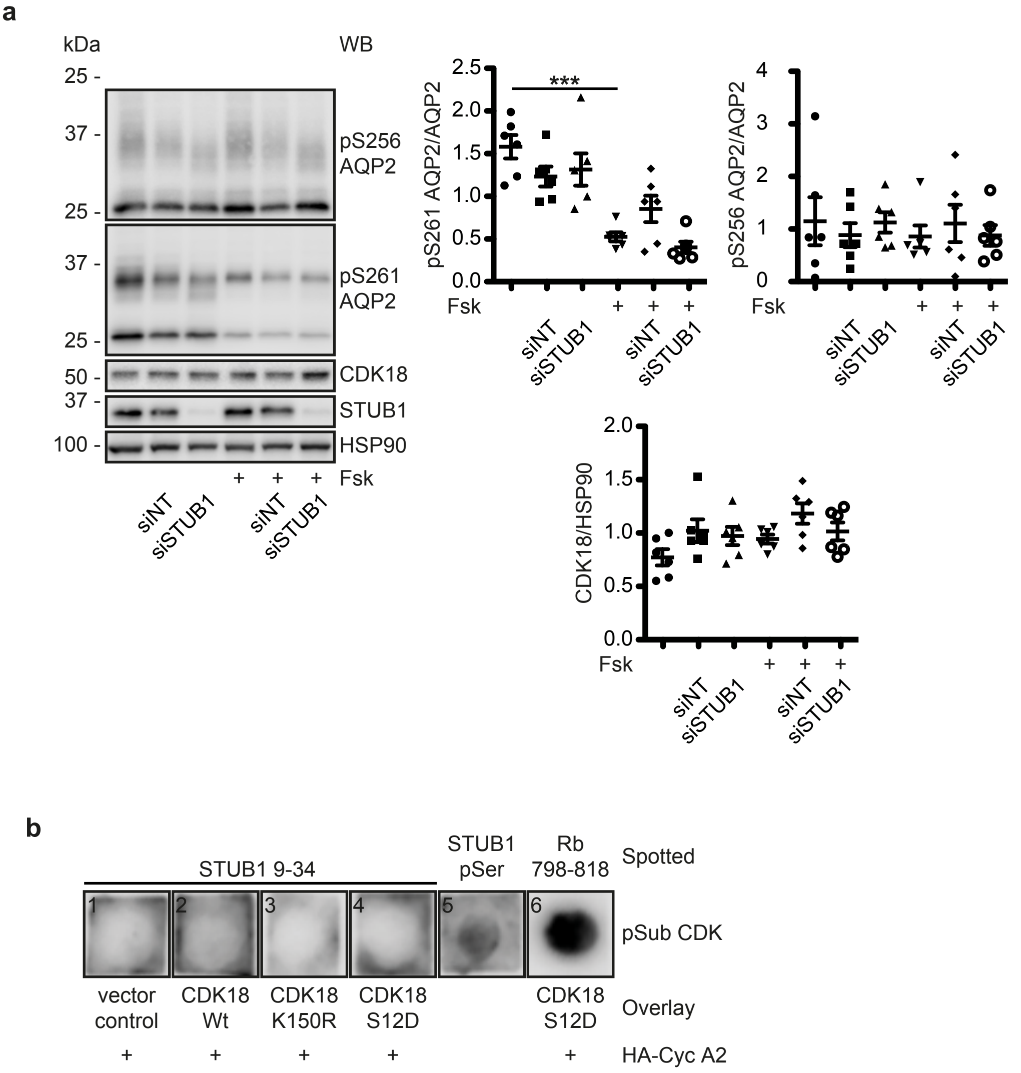

Figure S2. STUB1 knockdown does not influence AQP2 phosphorylation or the abundance of CDK18. A. MCD4 cells were left untreated or transfected with control non-targeting (NT) or STUB1 siRNA, and stimulated with Forskolin (Fsk). The cells were lysed and the indicated proteins detected by by Western blot (WB) analysis. Right panels. The signals were densitometrically evaluated. Shown are representative results from n = 6 (in A) independent experiments. Statistically significant differences are indicated, ***p <0.001. **B.** CDK18 does not phosphorylate STUB1. HEK293 cells, transfected with plasmids encoding the co-activator Cyclin A2, E.v.: empty vector as negative control, CDK18 Wild type (Wt), kinase dead (K150R) or constitutively active (S12A), were lysed and the different CDK18 forms were immuno-purified with anti-FLAG beads. The CDK18 precipitates were incubated on peptide-spotted murine STUB1 fragment 9-34 (spots 1-4), or as a positive control, on Rb fragment 792-818 (spot 6). Spot 5 contained pre-phosphorylated STUB1. The phosphorylated STUB1 and Rb peptides were detected using an anti-phospho-substrate CDK/MAPK (pSub CDK) antibody, following a standard Western blotting procedure (n = 4).

Figure S3

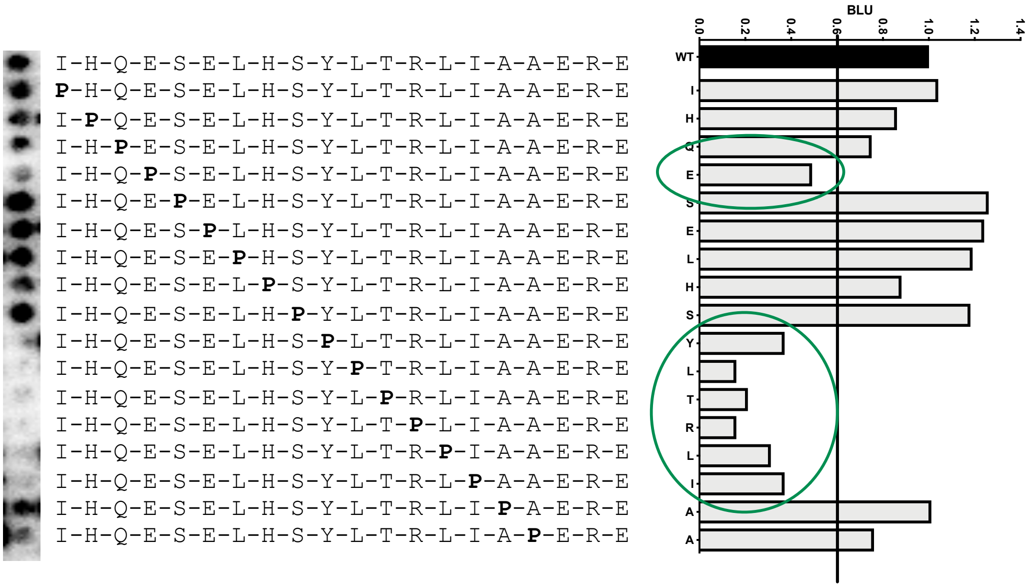

Figure S3. Mapping the STUB1 amino acids interacting with RII subunits of PKA.The indicated mouse STUB1 peptides were spot-synthesised as wildtype sequence (top) or with individual proline substitutions at the indicated positions. The spots were overlaid with P^{32}-labeled recombinant human RIIα subunits of PKA. No or a lower signal than wild type demonstrates that the substituted amino acid is essential for binding PKA. The semi-quantitative densitometric analysis is shown on the right.
